# The kynurenic acid analog SZR104 induces cytomorphological changes associated with the anti-inflammatory phenotype in cultured microglia

**DOI:** 10.1038/s41598-023-38107-8

**Published:** 2023-07-13

**Authors:** Melinda Szabo, Noémi Lajkó, Karolina Dulka, Gábor Barczánfalvi, Bálint Lőrinczi, István Szatmári, András Mihály, László Vécsei, Karoly Gulya

**Affiliations:** 1 Department of Cell Biology and Molecular Medicine, University of Szeged, Somogyi utca 4., 6720 Szeged, Hungary; 2 ELKH–SZTE Stereochemistry Research Group, Institute of Pharmaceutical Chemistry, University of Szeged, 6720 Szeged, Hungary; 3 Institute of Pharmaceutical Chemistry and Interdisciplinary Excellence Center, University of Szeged, 6720 Szeged, Hungary; 4 Department of Anatomy, University of Szeged, 6724 Szeged, Hungary; 5 Department of Neurology, University of Szeged, 6725 Szeged, Hungary; 6 ELKH–SZTE Neuroscience Research Group, Department of Neurology, Interdisciplinary Excellence Center, University of Szeged, 6725 Szeged, Hungary

**Keywords:** Microglia, Cellular imaging

## Abstract

We previously showed the anti-inflammatory effects of kynurenic acid (KYNA) and its brain-penetrable analog *N*-(2-(dimethylamino)ethyl)-3-(morpholinomethyl)-4-hydroxyquinoline-2-carboxamide (SZR104) both in vivo and in vitro. Here, we identified the cytomorphological effects of KYNA and SZR104 in secondary microglial cultures established from newborn rat forebrains. We quantitatively analyzed selected morphological aspects of microglia in control (unchallenged), lipopolysaccharide (LPS)-treated (challenged), KYNA- or SZR104-treated, and LPS + KYNA or LPS + SZR104-treated cultures. Multicolor immunofluorescence labeling followed by morphometric analysis (area, perimeter, transformation index, lacunarity, density, span ratio, maximum span across the convex hull, hull circularity, hull area, hull perimeter, max/min radii, mean radius, diameter of bounding circle, fractal dimension, roughness, circularity) on binary (digital) silhouettes of the microglia revealed their morphological plasticity under experimental conditions. SZR104 and, to a lesser degree, KYNA inhibited proinflammatory phenotypic changes. For example, SZR104 treatment resulted in hypertrophied microglia characterized by a swollen cell body, enlarged perimeter, increased transformation index/decreased circularity, increased convex hull values (area, perimeter, mean radius, maximum span, diameter of the bounding circle and hull circularity), altered box-counting parameters (such as fractal dimension), and increased roughness/decreased density. Taken together, analysis of cytomorphological features could contribute to the characterization of the anti-inflammatory activity of SZR104 on cultured microglia.

## Introduction

Microglial cells are macrophage-like cells of mesodermal origin that migrate early during the ontogenetic development to the central nervous system (CNS), where they play important physiological and pathophysiological roles^1,2^. Under physiological conditions in the CNS, most microglia display a ramified morphology^3,4^. However, inflammatory signals or injury elicit a variety of morphological and functional changes in these cells, reshaping them into an activated, amoeboid form both in vivo^5,6^ and in vitro^7–10^. Activated microglia not only exhibits phagocytosis, antigen presentation^11,12^, and altered gene expression patterns^7,8,13^ but dynamically remodeled membrane structures such lamellipodia, pseudopodia, filopodia, and podosomes^10,14,15^.

Kynurenic acid (KYNA), an endogenous antagonist for several neurotransmitter receptors such as ionotropic glutamate receptors^16^, influences important (patho)physiological processes including neuroinflammation^17^, neurodegeneration and -protection^18–21^ as well as some psychiatric diseases^22^. Pharmacological experiments demonstrated, for example, that KYNA exerts a neuroprotective role in various inflammatory and/or neurodegenerative CNS disorders^23^. KYNA is not a viable therapeutic drug as it cannot traverse the blood–brain barrier (BBB); however, brain-penetrable analogs could have the potential to ameliorate neurodegenerative or -inflammatory disorders^24^. One such analog, *N*-(2-(dimethylamino)ethyl)-3-(morpholinomethyl)-4-hydroxyquinoline-2-carboxamide (SZR104), has been recently shown to cross the BBB^25^ and successfully applied to pentylenetetrazol-induced epileptiform seizures, significantly decreasing the seizure-evoked field potentials^24^. Both KYNA and SZR104 exhibit anti-inflammatory properties under in vitro and in vivo conditions as they not only show potent immunosuppressive capacities in an animal model of epilepsy and inhibition of phagocytosis in microglial cultures^26^ but decreased the levels of the inflammatory marker proteins C–X–C motif chemokine ligand 10 (CXCL10) and C–C motif chemokine receptor 1 (CCR1) after LPS-treatment in microglial cultures^27^.

This study was, in part, motivated by the apparent similarities in cytomorphological features observed by treatment with SZR104 and with some other well-established, structurally unrelated anti-inflammatory drugs such as rosuvastatin (RST) and aspirin (ASP). Previously, we demonstrated that the proinflammatory LPS and the anti-inflammatory drugs RST and ASP dynamically and conversely altered not only phagocytosis and the expression of some inflammatory genes but the morphology of microglia^7,8^. Further, we showed the involvement of the actin cytoskeleton in such morphological changes^14^. Thus, the present study aimed to quantitatively analyze the effects of LPS, KYNA, and SZR104 on selected cytomorphological features (fractal dimension, lacunarity, area, perimeter, transformation index, circularity, hull area, hull perimeter, hull circularity, mean radius, maximum span across the convex hull, diameter of the bounding circle, max/min radii, span ratio, density, roughness) of binary (digital) silhouettes of control (unchallenged) and LPS-challenged microglial cells with or without KYNA or SZR104 treatment. We used these parameters as most have already been successfully used to characterize microglia in vivo^28–30^. Our study demonstrates that certain morphometric features are characteristic to the anti-inflammatory form, developed as a response to SZR104 treatment, of microglia under in vitro conditions.

## Results

### Image analysis pipeline

**Figure 1:**
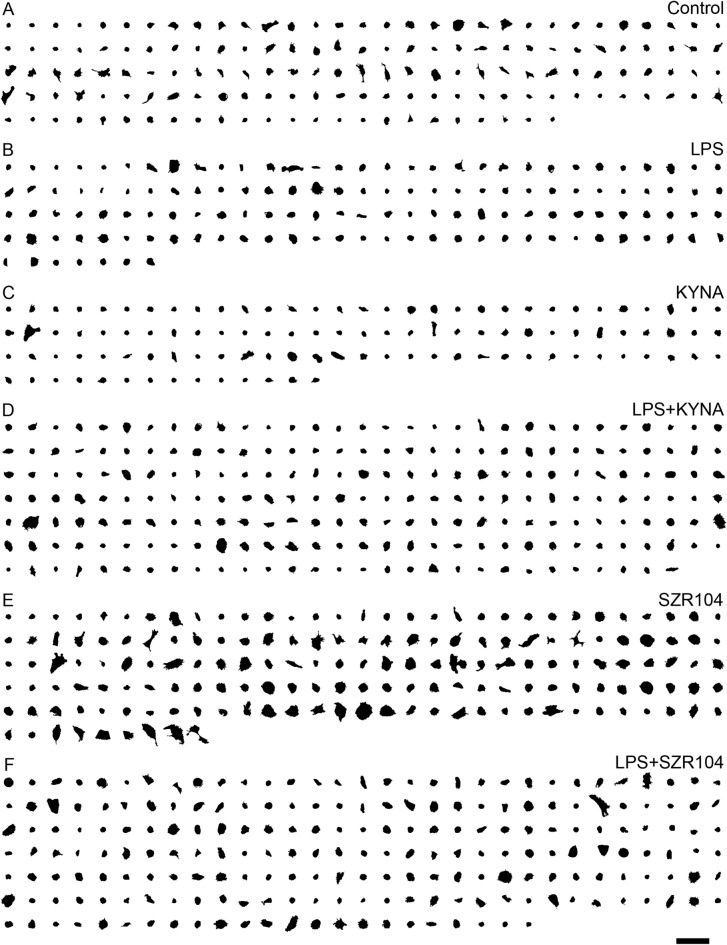
Morphological heterogeneity of microglial cells in control and treated cultures. CD11b/c-positive microglial cells from cultures under different treatments were photographed and the digital images (silhouettes) processed for analysis. Scale bar: 100 μm.

The complete set of CD11b/c-immunopositive cells (a total of 974 microglia) from the control and treated cultures analyzed for this study is shown in Supplementary Fig. S1. The image processing and measurements pipeline is depicted in Supplementary Figs. S2–S4. At the end of the process, CD11b/c-positive microglial images were converted into binary replicas using thresholding procedures implemented by ImageJ and FracLac (Fig. 1). The quantitative cytomorphological parameter values of individual microglial cells from control and experimentally treated cultures are shown in Supplementary Tables S1 and S2.

### Different culturing environments affect microglia morphology

**Figure 2:**
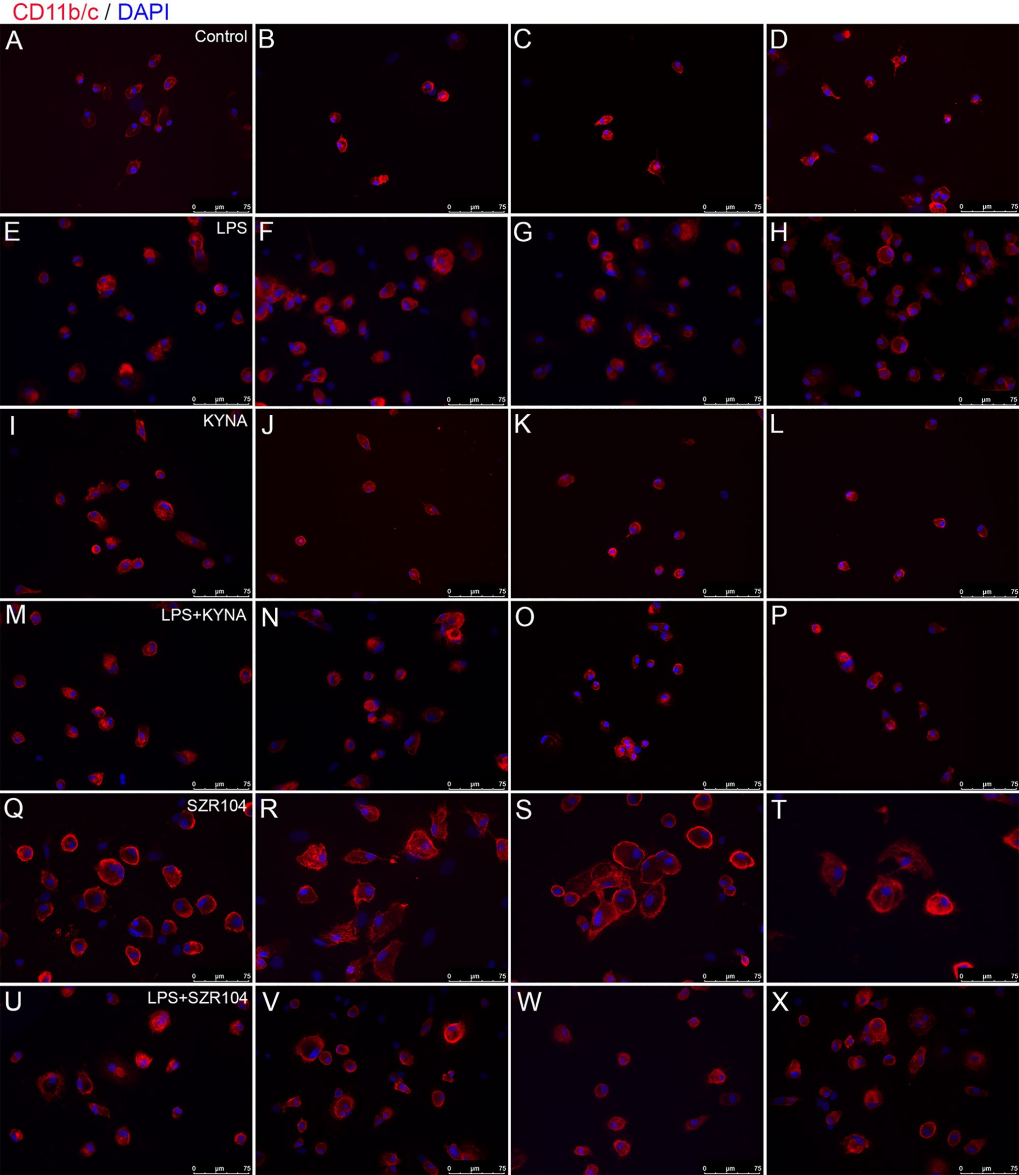
Immunocytochemical localization of microglial cells in secondary cultures. Secondary, microglia-enriched cultures were prepared and maintained as described in the Materials and Methods. A total of 974 cells were counted under a fluorescence microscope using a 20× or 40× objective. Representative immunocytochemical pictures from unstimulated/control (**A**–**D**), LPS- (**E**–**H**), KYNA- (**I**–**L**), LPS + KYNA- (**M**–**P**), SZR104- (**Q**–**T**) and LPS + SZR104-treated cultures (**U**–**X**). Note the varying levels of morphological heterogeneity of the microglial cells in control (unchallenged) and treated cultures. SZR104-treated cultures included mostly hypertrophied microglia (**Q**–**T**). Scale bar: 75 μm.

Fluorescent immunohistochemistry analysis showed a mixed population of ameboid and slightly ramified CD11b/c-labeled microglial cells in control (unstimulated) secondary, microglia-enriched cultures (DIV7) (Figs. 1A, 2A–D). Due to the relatively short culturing time, these microglia had only a few short branching processes. As expected, the LPS immunochallenge activated microglia, becoming more reactive and showing a more ameboid body without ramifications (Figs. 1B, 2E–H) compared to control cells. Under the microscope, KYNA-treated cells had relatively small cell bodies, occasionally slightly swollen and barely ramified, sometimes with short filopodia-like processes (Figs. 1C, 2I–L), which did not look significantly different from the controls. Unlike KYNA, its analog SZR104 produced the most profound effects on microglial morphology. SZR104-treated microglia became enlarged and rarely ramified but often displayed filopodia-like processes (Figs. 1E, 2Q–T). Such hypertrophied, swollen cell forms accounted for almost half of the examined microglia in the SZR104-treated group. When SZR104 was added to LPS-challenged cultures (Figs. 1F, 2U–X), the microglial morphology was less hypertrophied and often displayed an intermediate phenotype between the control and LPS-challenged cells, with a less pronounced ameboid shape.

### SZR104 elicits cytomorphological changes associated with the anti-inflammatory phenotype

**Figure 3:**
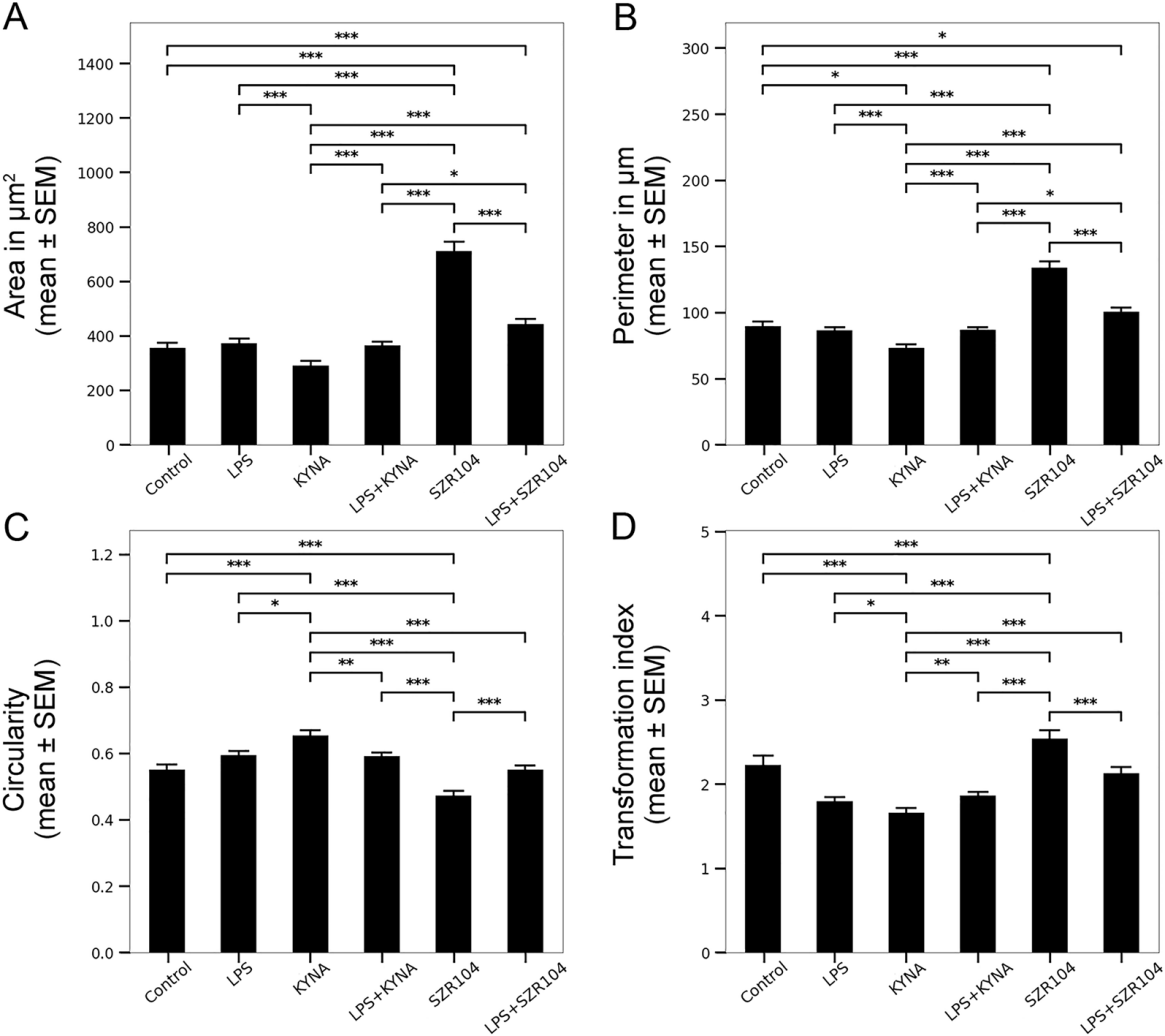
Morphological heterogeneity of microglial cells under various conditions as evidenced by the area, perimeter, circularity, and transformation index values. CD11b/c-positive microglial cells from the control and differently treated cultures were photographed, digitized, and quantitatively analyzed according to their morphological characteristics. Area (**A**; μm^2^), perimeter (**B**; μm), circularity (**C**) and transformation index (**D**) values.

Quantitative analysis on the binary silhouettes of the microglia revealed that several parameters changed significantly with the different treatments (Supplementary Tables S1, S2). According to the cell area and perimeter values, as well as their derived parameters, the circularity and transformation index, microglia treated with SZR104 showed the most obvious changes (Fig. 3). For example, while control microglia had area values of 356.08 ± 18.10 μm^2^, perimeter values of 89.72 ± 3.43 μm, circularity values of 0.55 ± 0.02, and transformation index values of 2.23 ± 0.11, SZR104-treated cells had area values of 710.79 ± 34.92 μm^2^, perimeter values of 134.19 ± 4.73 μm, circularity values of 0.47 ± 0.01 and transformation index values of 2.54 ± 0.10, respectively (Fig. 3A–D, Supplementary Tables S1, S2). The significantly increased area, perimeter, and transformation index values, together with the significantly decreased circularity value, strongly indicate SZR1040’s inhibitory effects on the development of a proinflammatory morphology in microglia.

**Figure 4:**
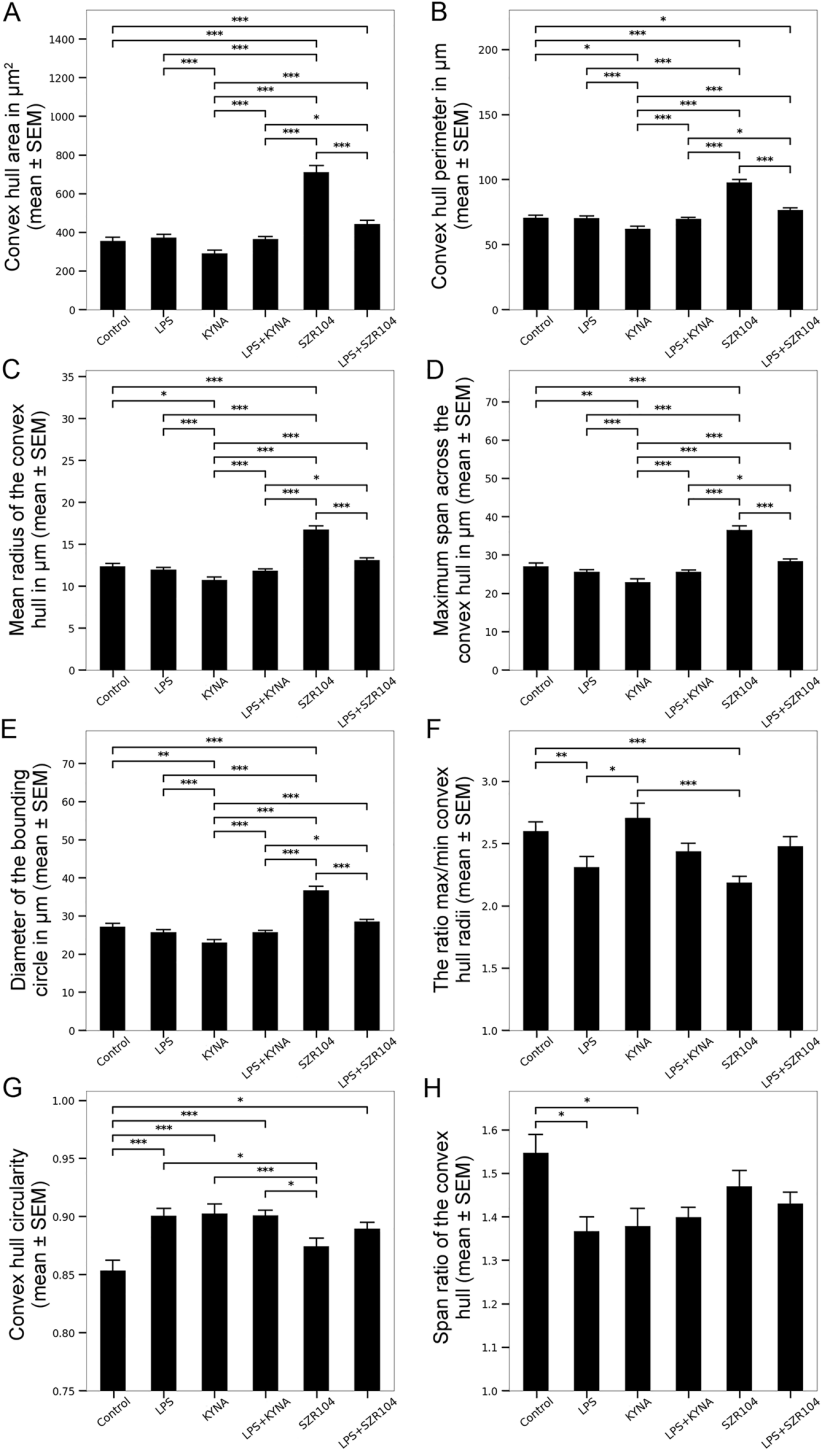
Morphological heterogeneity of microglial cells under various conditions as evidenced by convex hull parameters (different aspects of the convex polygons, with all interior angles < 180°, containing the whole cell shape). Various convex hull measurements (**A**–**H**) were performed in each group in terms of area (μm^2^) and distance (μm). Convex hull area (**A**), convex hull perimeter (**B**), mean radius of the convex hull (**C**), maximum span across the convex hull (**D**), diameter of the bounding circle (**E**), the ration max/min convex hull radii (**F**), convex hull circularity (**G**) and span ratio of the convex hull (**H**) were quantitatively analyzed.

When convex hull parameters were examined using FracLac, a more differentiated pictures emerged. The area of the convex hull (Fig. 4A), the perimeter (Fig. 4B), the mean radius (Fig. 4C), the maximum span across it (Fig. 4D), and the diameter of the bounding circle (Fig. 4E) consistently showed the largest significant differences between controls and SZR104-treated cells. While the maximum/minimum ratio of the convex hull, also showed significantly different values between control (2.60 ± 0.08) and SZR104-treated cells (2.19 ± 0.05; Fig. 4F), other dimensionless hull values, such as the convex hull circularity and the span ratio of the convex hull, showed no significant differences between these two groups (Fig. 4G, H). Most convex hull parameter values for SZR104-treated microglia indicated an increased cell size, a measure often indicative of hypertrophied and swollen cell bodies.

**Figure 5:**
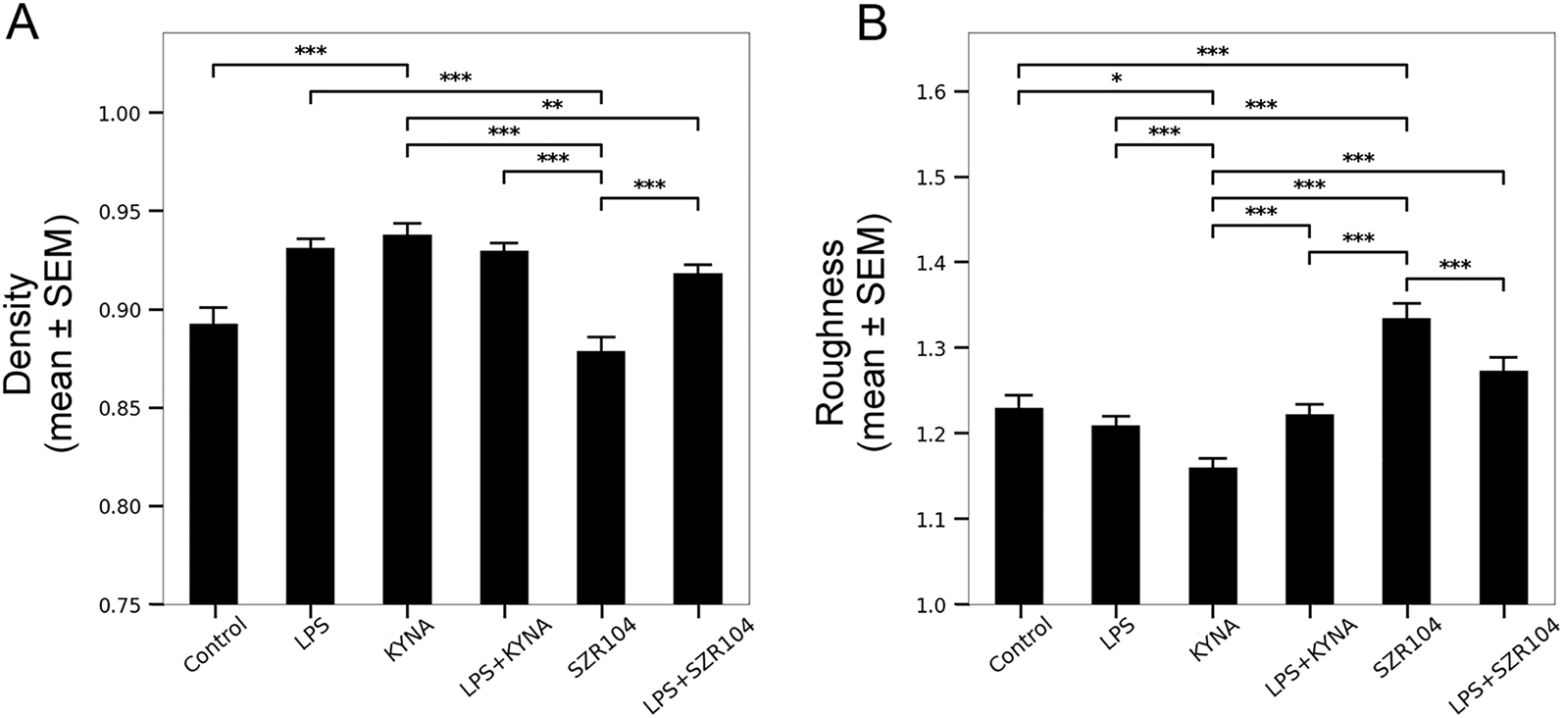
Morphological heterogeneity of microglial cells under various conditions as evidenced by density (**A**) and roughness (**B**) values. Density and roughness are two additional, dimensionless parameters that can be derived from the area, the perimeter, and the convex hull measurements.

Density and roughness, dimensionless parameters defined as the ratios of cell areas or perimeters to convex hull areas or perimeters, showed the largest differences between control and SZR104-treated microglia (Fig. 5A, B). A decreased density is indicative of a less compact cell, an obvious characteristic of SZR104-treated microglia (Fig. 5A). In these cells, the density was the smallest (0.878695 ± 0.007411) while the roughness was the largest (1.334733 ± 0.017267), indicating that this analog affected cell morphology the most. Interestingly, the roughness value was significantly higher in microglia treated with SZR104 than in any other group (Fig. 5B). Taken together, both density and roughness values indicated that SZR104 inhibited the proinflammatory morphology of microglia.

**Figure 6:**
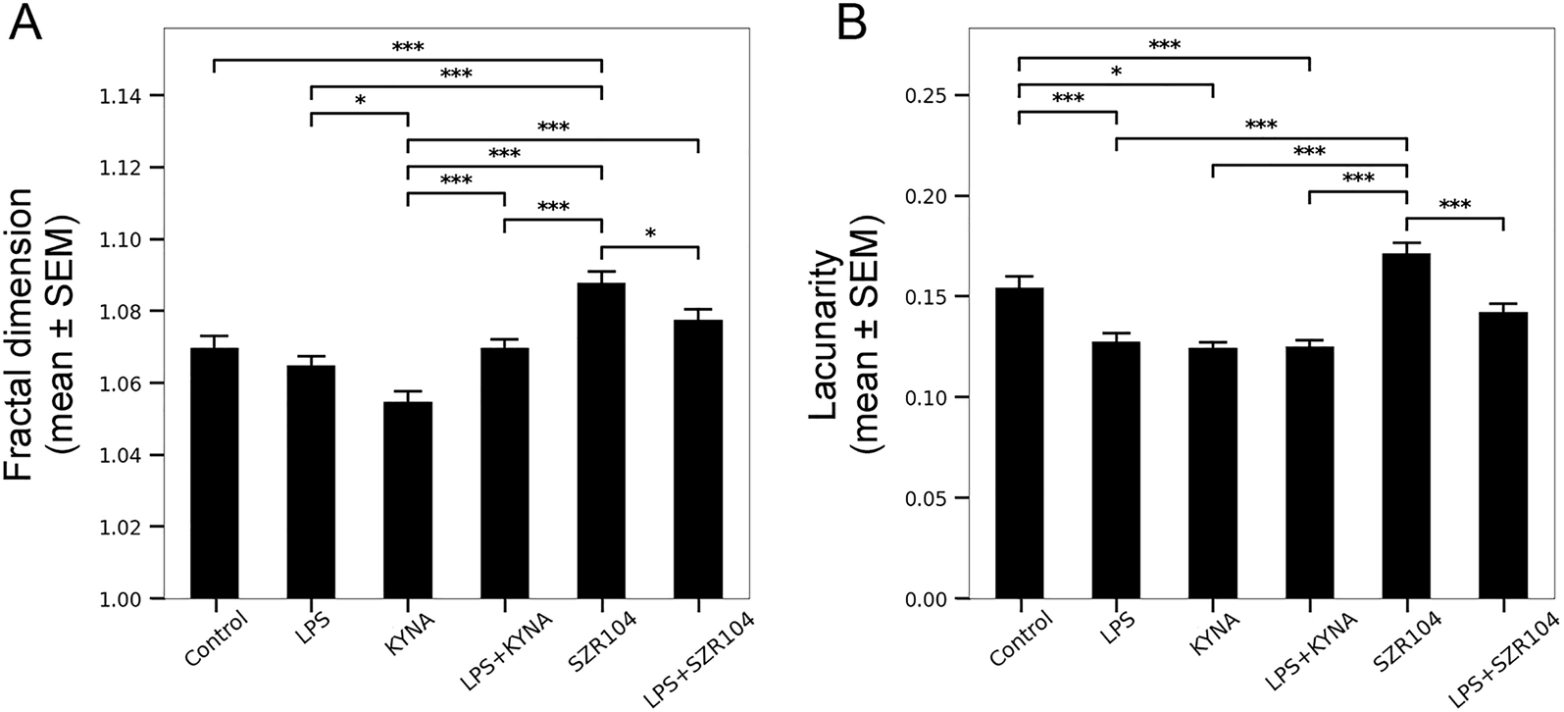
Morphological heterogeneity of microglial cells under various conditions as evidenced by the fractal dimension (**A**) and lacunarity (**B**) parameters. Morphological complexity was analyzed using fractal analysis to obtain fractal dimension and lacunarity (translational and rotational invariance) values. Fractal dimension and lacunarity were measured by the box counting method.

Finally, fractal dimension and lacunarity, determined by the box counting method, were analyzed to determine the heterogeneity and complexity of the cell morphology (Fig. 6). Activation of microglia by LPS and KYNA was evidenced by lower fractal dimension (Fig. 6A) and lacunarity (Fig. 6B), a measure of translational and rotational invariance and increased heterogeneity, respectively. The fractal dimension showed a significant difference (p < 0.001) between control (1.069746 ± 0.003361) and SZR104-treated cells (1.087849 ± 0.003153; Fig. 6A), indicating an increase in pattern complexity. Interestingly, this parameter differed the most between cells treated either with KYNA or its analog SZR104. Whereas KYNA treatment tended to lead to a simpler geometry, SZR104 treatment resulted in more complex cell forms (Figs. 1, 6A). Increased lacunarity was observed in SZR104-treated microglia (Fig. 6B); in fact, SZR104 treatment resulted in the largest lacunarity values among experimental groups (0.171496 ± 0.005049), even if not significantly different from control microglia (0.154234 ± 0.005571). Taken together, fractal dimension and lacunarity parameters indicated a strong anti-inflammatory effect of SZR104 on the microglial cells. All cytomorphological parameters that could collectively point to the anti-inflammatory activity of SZR104 are summarized in Table 1.

**Table 1:** SZR104 promotes microglia polarization toward anti‐inflammatory (M2) phenotype.

Cytomorphological features analyzed in automated image analysis workflow	Change after SZR104 as compared to control	*p*	Change after SZR104 as compared to LPS challenge	*p*	Change toward anti-inflammatory phenotype
Area	Up	***	Up	***	Yes
Perimeter	Up	***	Up	***	Yes
Circularity	Down	***	Down	***	Yes
Transformation index	Up	***	Up	***	Yes
Convex hull area	Up	***	Up	***	Yes
Convex hull perimeter	Up	***	Up	***	Yes
Mean radius of the convex hull	Up	***	Up	***	Yes
Maximum span across the convex hull	Up	***	Up	***	Yes
Diameter of the bounding circle	Up	***	Up	***	Yes
The ratio max/min convex hull radii	Down	***	Down	N.S.	Likely
Convex hull circularity	Up	N.S.	Up	**	Yes
Span ratio of the convex hull	Down	N.S.	Down	N.S.	Likely
Density	Down	N.S.	Down	***	Yes
Roughness	Up	***	Up	***	Yes
Fractal dimension	Up	***	Up	***	Yes
Lacunarity	Up	N.S.	Up	***	Yes

The anti-inflammatory potential (M2 polarization) of SZR104 was classified according to the changes it caused on selected cytomorphological features of cultured microglial cells [see refs.^7,8,28,29,57,60^]. Significant changes (*p*) are indicated as: *** (*p* < 0.005), ** (*p* < 0.05). N.S.: non-significant.

## Discussion

Microglial morphology under physiological and pathophysiological states^31,32^ have been extensively analyzed both in vivo^28^ and in vitro^7,8,10^. Under in vivo conditions, microglial morphology is greatly influenced by the age of the host^33^ and the surrounding tissue cytoarchitecture^34^ resulting in a characteristic spatial arrangement of their branches that are sometimes difficult to precisely reconstruct. While microglial cells also show a wide range of morphological differentiation in vivo^28,29^, their ramified processes are flattened to a monolayer during culturing and thus analyzing their digital silhouettes easily^10,35^.

The morphological changes in microglia occurring in response to various physiological and pathological conditions are well documented. Upon activation, sedentary, ramified microglia became reactive, often displaying ameboid shape and a unique set of immunoreactive markers^1,12^. Microglial hypertrophy has been studied extensively in vivo in several mammalian species including mice^36^, rats^37^, and humans^38^. When activated by physiological stimuli such as salt stress^37^, diabetic induction^39^, high fat diet^36^, neuronal trauma^40^, infection^41^ or neurodegenerative stimuli^38^, microglia became hypertrophied. Interestingly, both ameboid and ramified microglia can become hypertrophic^42^. Although hypertrophic microglia are sometimes indicative of neuronal tissue injury^36,43^, this microglial phenotype is dominant during physiological activation as well^37^. Moreover, a recent study observed dystrophic but not hypertrophied microglia in Alzheimer’s disease^38^.

The quantitative morphology parameters for SZR104 consistently showed an anti-inflammatory drug in action. While the ramified, surveillant phenotype of the microglia with a relatively small cell body and long primary and thin secondary branches display a high fractal dimension as well as high lacunarity and perimeter values together with low density and typically larger hull density values, the ameboid, activated, phagocytosis-capable microglia is characterized by small, roundish cell bodies without branches that would be associated to low fractal dimension, lacunarity and perimeter values as well as typically lower hull density values. This latter form corresponds to the activated, proinflammatory phenotype, capable of avid phagocytosis and proinflammatory cytokine production. Conversely, microglia treated with SZR104 show an increased area, perimeter, and transformation index values, as well as a decreased circularity value and high complex hull, density, and roughness values, all indicative of hypertrophied and swollen cell bodies. These findings further support that SZR104 inhibited the proinflammatory morphology in microglia induced by LPS. Previous in vivo and in vitro studies suggested that SZR104 is an anti-inflammatory agent^17,26,27,44^. In an animal model of epilepsy, for example, we showed that SZR104 potently inhibited both the synthesis of the proinflammatory IL-6 in the hippocampus during pilocarpine-induced seizures and the proinflammatory morphological transformation of microglia following status epilepticus^26^. Moreover, we also showed that SZR104 strongly inhibited microglial phagocytosis^26^ and the synthesis of the inflammatory marker proteins CXCL10 and CCR1 after LPS-treatment in microglial cultures^27^. Furthermore, our university-wide KYNA research project (GINOP 2.3.2-15-2016-00034) demonstrated that SZR104 not only inhibited tumor necrosis factor-α (TNF-α) synthesis, a molecule known for its proinflammatory function, and increased the expression of the anti-inflammatory gene TNF-Stimulated Gene-6 in U-937 cells after immunochallenge^17^, but also protected against sepsis-associated neutrophil activation and brain mitochondrial dysfunction in rats^44^. Thus, our previous in vivo and in vitro results and the present in vitro data seem to support the anti-inflammatory effects of SZR104.

Our previous in vitro studies also showed that hypertrophic phenotypes can also be of anti-inflammatory nature, as observed in RST- and ASP-activated hypertrophic microglia^7,8^. We found that these two widely used pleiotropic drugs displaying anti-inflammatory properties, affected microglia very similarly after LPS immunochallenge in culture^7,8^. When secondary microglia were cultured for a week and treated with SZR104, RST, or ASP cells displayed a distinct morphological characteristic with transformation index values significantly higher than those in the control (unchallenged) or LPS-treated microglia cultures. This similarity was observed irrespectively to the purity of the microglial cultures used as microglia-enriched secondary cultures for KYNA and SZR104 had, on average, 78% of purity, while pure microglial cultures for RST and ASP had > 99% purity^7,8^. When treated with SZR104, RST or ASP, microglial cells acted very similarly and became enlarged, often with two-fold increase in their area, and developed short filopodia and pseudopodia, effectively increasing their perimeter. The combined treatment of LPS with either SZR104, RST^7^ or ASP^8^ resulted in a similarly elevated area, perimeter, and transformation index values of the cultured microglia.

Phenotypic plasticity of microglia is a feature of the normal structural remodeling that accompanies microglial activation^37^, where hypertrophy of cytoplasm is a characteristic feature of the activated microglia^12,36^. Based on our present quantitative measurements, we assume that the hypertrophic enlargement of the cell somata and filopodia formation exemplify anti-inflammatory processes in microglia. The cytomorphological changes elicited by SZR104 were like those of other anti-inflammatory drugs such as RST and ASP and point out to the involvement of actin cytoskeleton^14^.

The synthetic SZR104 is a structural analog of the endogenous KYNA. It must be noted that despite their chemical similarity, they are not necessarily functional analogs in every aspect; indeed, they have different physiological, biochemical, pharmacological, or cytomorphological properties (for example, while KYNA is an endogenous compound, SZR104 is a synthetic molecule; KYNA cannot pass the blood–brain barrier, SZR104 can; both have anti-inflammatory properties, etc.). Studies on the relationship between molecular and biological similarity showed that cell morphology changes and chemical structure differences contain valuable information for the prediction of biological activity^45–47^. While the extensive molecular structure modifications of SZR104 certainly attributed to its improved anti-inflammatory characteristics as compared to that of KYNA, the mode of action is still undetermined.

Collectively, our quantitative morphology data strongly indicate that SZR104 is a more potent anti-inflammatory activator of microglia than the physiologic KYNA. As it can also cross the BBB, SZR104 could be a potential target molecule to explore the endogenous KYNA pathway when applied in vivo. In conclusion, our cytomorphology-based analysis can provide useful information for predicting a measure of biological activity, such as the anti-inflammatory nature, of a KYNA analog.

## Materials and methods

### Animals

All animal experiments were carried out in strict compliance with the European Council Directive (86/609/EEC) and EC regulations (O.J. of EC No. L 358/1, 18/12/1986) regarding the care and use of laboratory animals for experimental procedures and followed relevant Hungarian and local legislation requirements. Our experimental protocols were approved by the Institutional Animal Welfare Committee of the University of Szeged (II./1131/2018). Pregnant Sprague–Dawley rats (190–210 g) were maintained under standard housing conditions and fed ad libitum.

### Reagents

KYNA was purchased from Sigma-Aldrich (Steinheim, Germany). The KYNA analog SZR104 was synthesized in house at the Institute of Pharmaceutical Chemistry, University of Szeged, Hungary. For this study, we further optimized the previously published synthesis of the amide and aminoalkylated derivatives^48,49^. The direct amidation was carried out in ethanol at reflux, using 1,2 equivalent of amine, 6 h reaction time (yield: 78%). The subsequent aminoalkylation was also optimized as the 3 equivalent aqueous formaldehyde previously described was replaced by a 1,2 equivalent of paraformaldehyde resulting in a yield of 89%^50^. KYNA and SZR104 were dissolved in phosphate buffered saline (PBS) and added to the cell cultures at a final concentration of 1 μM. Unless stated otherwise, all reagents were purchased from Sigma (St. Louis, MO, USA).

### Preparation of high-purity microglial cultures

Mixed primary neuron/glia cultures were established from newborn rats of both sexes. The animals were decapitated in accordance with ARRIVE guidelines (https://arriveguidelines.org/arrive-guidelines/experimental-procedures) and AVMA Guidelines for the Euthanasia of Animals (https://www.avma.org/sites/default/files/2020-02/Guidelines-on-Euthanasia-2020.pdf; see Section M3.7 for explanation). The brains were quickly removed and cleared from the meninges. The forebrains were minced with scissors and incubated in 9 mL of Dulbecco’s Modified Eagle’s Medium (DMEM; Invitrogen, Carlsbad, CA, USA) containing 1 g/L of d-glucose, 110 mg/L of Na-pyruvate, 4 mM L-glutamine, 3.7 g/L of NaHCO_3_, 10,000 U/mL of penicillin G, 10 mg/mL of streptomycin sulfate, and 25 μg/mL of amphotericin B, supplemented with 1 mL 2.5% trypsin (Invitrogen) for 10 min at 37 °C, followed by centrifugation at 1000 × *g* for 10 min at room temperature (RT)^10,51,52^. The pellet was resuspended in 10 mL DMEM containing 10% heat-inactivated fetal bovine serum (FBS; Invitrogen) and filtered using a sterile filter (100 µm pore size; Greiner Bio-One Hungary Kft., Mosonmagyaróvár, Hungary), to eliminate the tissue fragments that resisted dissociation. The filtered cell suspension was centrifuged for 10 min at 1000 × *g* at RT and the pellet resuspended in 5 mL DMEM/10% FBS; then, the primary mixed cells were seeded in the same medium on poly-L-lysine-coated culture flasks (75 cm^2^; 10^7^ cells/flask). The number of collected cells was determined in a Bürker chamber after trypan blue staining. The cultures were maintained at 37 °C in a humidified air atmosphere supplemented with 5% CO_2_. The medium was changed the following day and on the fourth day.

The preparation of secondary, microglia-enriched cultures from mixed primary forebrain cultures followed a slightly modified version of our previous protocol^51^. Briefly, after seven days of culture, the microglial cells in the primary cultures were shaken off using a platform shaker (120 rpm for 20 min) at 37 °C, collected from the supernatant by centrifugation (3000 × *g* for 8 min at RT), resuspended in 4 mL of DMEM/10% FBS and plated in the same medium on poly-l-lysine-coated coverslips (15 × 15 mm; 2 × 10^5^ cells/coverslip) for immunocytochemistry^51^. The number of collected cells was determined in a Bürker chamber after trypan blue staining. After allowing the cells to adhere to the poly-l-lysine-coated surface for 30 min, the supernatant which may contain floating cells was carefully removed and cell culture medium (DMEM/10% FBS) was added to the cells. On the sixth day of subcloning, the culture medium was replaced, and the microglia were treated for 24 h with either LPS (20 ng/mL), KYNA (1 μM), or SZR104 (1 μM) alone, or with a combination of LPS + KYNA or LPS + SZR104. LPS treatment served as immunochallenge. Microglial cells seeded on coverslips were fixed in 0.05 M PBS (pH 7.4 at RT) containing 4% formaldehyde for 10 min at RT and stored at – 20 °C until use.

### Fluorescent immunocytochemistry

For the identification of microglial cells, we used the anti-CD11b/c antibody (clone OX42) as it recognizes a common epitope shared between CD11b and CD11c (integrin αM and αX chains) and reacts with all monocytes and macrophages^53,54^, identifying resident microglia in the CNS^10^. Light microscopic immunofluorescence labeling was performed as previously described^51^. Fixed secondary cultures on coverslips were washed three times for 5 min each in 0.05 M PBS. After permeabilization and blocking of nonspecific sites for 30 min at 37 °C in 0.05 M PBS containing 5% normal goat serum, 1% heat-inactivated FBS albumin, and 0.05% Triton X-100, the cells on the coverslips were incubated overnight at 4 °C with the microglia-specific mouse anti-CD11b/c monoclonal primary antibody (1:100 final dilution; Abcam, Cambridge, England). The cultured cells were then washed four times for 10 min each at RT in 0.05 M PBS and incubated with the goat anti-mouse Alexa Fluor 568 fluorochrome-conjugated secondary antibody (final dilution: 1/1000; Invitrogen, Carlsbad, CA, USA, without Triton X-100) in the dark for 3 h at RT. The cells on the coverslip were washed four times for 10 min each in 0.05 M PBS at RT, rinsed in distilled water, and mounted on microscope slides covered with Prolong Diamond Antifade with DAPI (ThermoFisher). To confirm the specificity of the secondary antibody, omission control experiments (i.e., staining without the primary antibody) were performed. In these experiments, no immunocytochemical signals were observed.

### Image analysis

Digital images were captured by a Leica DMLB epifluorescence microscope using a Leica DFC7000 T CCD camera (Leica Microsystems CMS GmbH, Wetzlar, Germany) and the LAS X Application Suite X (Leica). For each culture, 15–20 randomly selected microscope fields per coverslip from ≥ 8 coverslips were counted and analyzed using the computer programs ImageJ (version 1.47; developed at the U.S. National Institutes of Health by W. Rasband, available at https://imagej.net/Downloads;^55^) and the plugin FracLac for ImageJ (https://imagej.nih.gov/ij/plugins/fraclac/fraclac.html;^56,57^). The complete set of CD11b/c-immunopositive cells analyzed for this study from the control and treated cultures is shown in Supplementary Fig. S1. For the measurements, CD11b/c-positive microglial images were converted into binary replicas using thresholding procedures implemented in ImageJ and FracLac. The image processing and measurements pipeline is depicted in Supplementary Figs. S2, S3. Digital images in tagged image file formats (.tif) were opened in ImageJ (Supplementary Fig. S2A). The measurements were performed on segmented (binary) and processed images of selected individual cells (400 × 400 pixel cropped images) identified by both the DAPI-labeled cell nuclei (Supplementary Fig. 2SB) and the CD11b/c-positive cytoplasm (Supplementary Fig. S2C). To this end, each fluorescence image obtained was processed as follows: the labeled cytoplasmic and nuclear compartments of individual microglial cells were identified on the two-channel image (Supplementary Fig. S2A), but the blue (Supplementary Fig. S2B) and the red (Supplementary Fig. S2C) color channel images were converted to 8-bit grayscale images and threshold separately selecting the following sequence of menus: Image → Color → Split channels/Merge channels/Channels Tool…, Image → Type → 8-bit (Supplementary Figs. S2D, E, F, S3G). Since the Alexa Fluor 568 fluorochrome-conjugated secondary antibody-labeled cytoplasm showed a weaker staining in the location of the cell nuclei, merging their binary silhouettes (cleared foreground pixels of threshold color channels) with the binary silhouettes of the corresponding cell nuclei proved beneficial in many cases, resulting in complementary silhouettes (Supplementary Fig. S3H, I, J). For this reason, two color channels (red and blue) were used to extract the binary silhouettes. Thresholding was performed separately on the images of the two-channels (after channel splitting and converting to 8-bit grayscale images; Supplementary Fig. S2D, E), since transforming them together into 8-bit grayscale images would lead to them canceling out each other's intensity values; further, after binarization, the cells could become connected to neighboring ones due to the parallel use of two-channels.

In most cases, a few simple ImageJ commands were sufficient to clean the binary silhouettes (Analyze→ Analyze Particles: Size: depending on the circumstances, Show: Masks, Exclude on edges; Process→ Binary: Fill holes, Watershed); not all commands were necessarily applied in each case (see^58^ for details, and the documentation web pages for ImageJ at http://rsb.info.nih.gov/ij/docs/index.html); however, in some cases manual editing was required to separate the microglial cells in the binarized images (Freehand selections, Edit → Cut, Wand tool, Edit→ Selection→ Make Inverse). The ImageJ Macro Recorder was used to generate macro codes to apply commands and automate image processing in some cases. For instance, Plugins→ Macros → Record… →’used commands’ → Create→ Run provided a cell profile formed by a continuous, filled set of pixels (Supplementary Fig. S3H, I, J). These final, clean, and merged binary silhouettes (Supplementary Fig. S3K) were used to measure most analyzed parameters (such as area, perimeter, lacunarity and convex hull measurements), but before measuring fractal dimension, we applied the Process→ Binary → Outline commands on the images (Supplementary Fig. S3K; see also the black contour lines enclosing the dark gray areas in Supplementary Fig. S3L). Using the FracLac plugin, Box Counting (BC) measurements were performed without selecting “Legacy model”. The process involved the following commands: “Use binary”, “Lock white as background”. In “Grid design” section, the Scaling Method of “Power series” with base 2 and exponent 2 was selected, and “Db” (to measure the fractal dimension by box counting) was chosen in the corresponding dialog box. Convex hull measurements were performed with the following settings: in “Grid design”, the number of grid orientations was set to 0, and in Graphics options, “Hull and circle” and “metrics” as well as “bounding circle” and “convex hull” were selected (black edges of convex polygons enclosing the dark gray areas and white concavities and black bounding circles enclosing the light gray parts as well) (Supplementary Figs. 3L, S4). The full set of binary silhouettes used in this study is shown in Fig. 1.

The quantitative parameters used to analyze microglial morphology (described in detail in^28,29,58–60^) were grouped into four classes: (1) fractal dimension and lacunarity (computed using box counting software); (2) area (number of pixels in the filled shape of the binary image, μm^2^), perimeter (μm), transformation index (determined according to Fujita et al.^61^ using the following formula: [perimeter of the cell (μm)]^2^/4π [cell area (μm^2^)]), and circularity (calculated as [4π × cell area (μm^2^)]/cell perimeter (μm)^2^]; note that transformation index = 1/circularity where the circularity value of a circle = 1); (3) convex hull area (smallest convex polygon containing the whole cell shape, μm^2^), convex hull perimeter (single outline of the convex hull, μm), convex hull circularity ([4π × convex hull area]/[convex hull perimeter]^2^; this value is 1 for a circle), mean radius of the convex hull (mean length, μm) from the center of mass of the convex hull to an exterior point, maximum span across the convex hull (maximum distance between two points across the convex hull, μm), diameter of the bounding circle (the smallest circle that encloses the convex hull, μm), max/min radii (ratio of the largest to the smallest radius from the center of mass of the convex hull to an exterior point), and span ratio of the convex hull (ratio of the major to the minor axes of the convex hull, as a measure of cell shape/elongation); and 4) density (ratio of the cell area to its convex hull area), and roughness (ratio of cell perimeter to convex hull perimeter) were analyzed using ImageJ and FracLac^28,29,55–57,59,60,62,63^.

### Statistical analysis

A total of 974 microglia, identified by the presence of the microglia-specific marker CD11b/c, were quantitatively analyzed: 148 controls, 131 LPS-treated, 107 KYNA-treated, 215 LPS + KYNA-treated, 164 SZR104-treated, and 209 LPS + SZR104-treated microglia. The statistical analysis on the measured cytomorphological parameters of the cells was performed in a Colab notebook of the Google Colaboratory (https://colab.research.google.com) running Python version 3.7.13^64,65^. The non-parametric Kruskal–Wallis H-test was performed using the function (scipy.stats.kruskal) of the SciPy scientific library (https://scipy.org)^66^ to determine whether there was a difference between the six groups with regard to the given parameters (Supplementary Table S1, S2). Mann–Whitney U post-hoc testing was performed using normal approximation, continuous distributions, and continuity correction since n1 > 20 and n2 > 20. The Bonferroni correction method was also applied for multiple comparisons (for controlling Type I error; https://scikit-image.org; see ref.^67^ for details) to indicate which groups are different. Python software libraries Pandas (ver. 1.4.4, https://pandas.pydata.org) and NumPy (ver. 1.23.0, https://numpy.org) were also used for basic data manipulation, formatting, and processing^68,69^. Values are indicated as the mean ± SEM. *p* < 0.05 was considered significant; *, **, and *** denote *p* < 0.05, *p* < 0.01, and *p* < 0.005, respectively.

### Ethics declarations

All applicable international, national, and/or institutional guidelines for the care and use of animals were followed. Experimental procedures were carried out in strict compliance with the European Communities Council Directive (86/609/EEC) and followed Hungarian and local legislation requirements (XXVIII/1998 and 243/1998) and university guidelines regarding the care and use of laboratory animals. This study is also in accordance with ARRIVE guidelines (https://arriveguidelines.org/arrive-guidelines/experimental-procedures) and with AVMA Guidelines for the Euthanasia of Animals (https://www.avma.org/resources-tools/avma-policies/avma-guidelines-euthanasia-animals). The experimental protocols were approved by the Institutional Animal Welfare Committee of the University of Szeged (II./1131/2018; date of approval: 30 May 2018).

## Supplementary Information

Supplementary Information.

## Data Availability

Data is contained within the article and Supplementary material.

## Abbreviations

ASP: Aspirin
BBB: Blood–brain barrier
CD11b/c: A common epitope shared between CD11b and CD11c (integrin αM and αX chains)
CNS: Central nervous system
DAPI: 2-(4-Amidinophenyl)-6-indolecarbamidine dihydrochloride
DMEM: Dulbecco’s Modified Eagle’s Medium
FBS: Fetal bovine serum
KYNA: Kynurenic acid
LPS: Lipopolysaccharide
PBS: Phosphate-buffered saline
rpm: Revolutions per minute
RST: Rosuvastatin
RT: Room temperature
SEM: Standard error of the mean
SZR104: *N*-(2-(Dimethylamino)ethyl)-3-(morpholinomethyl)-4-hydroxyquinoline-2-carboxamide
