# Supplementary material for: The kynurenic acid analog SZR104 induces cytomorphological changes associated with the anti-inflammatory phenotype in cultured microglia
Source: Sci Rep. 2023 Jul 13;13:11328. doi: 10.1038/s41598-023-38107-8 (PMC10344911; doi:10.1038/s41598-023-38107-8)
Supplement: Supplementary file 1 — Supplementary Information. [file 41598_2023_38107_MOESM1_ESM.pdf]

Supplementary Figure S1

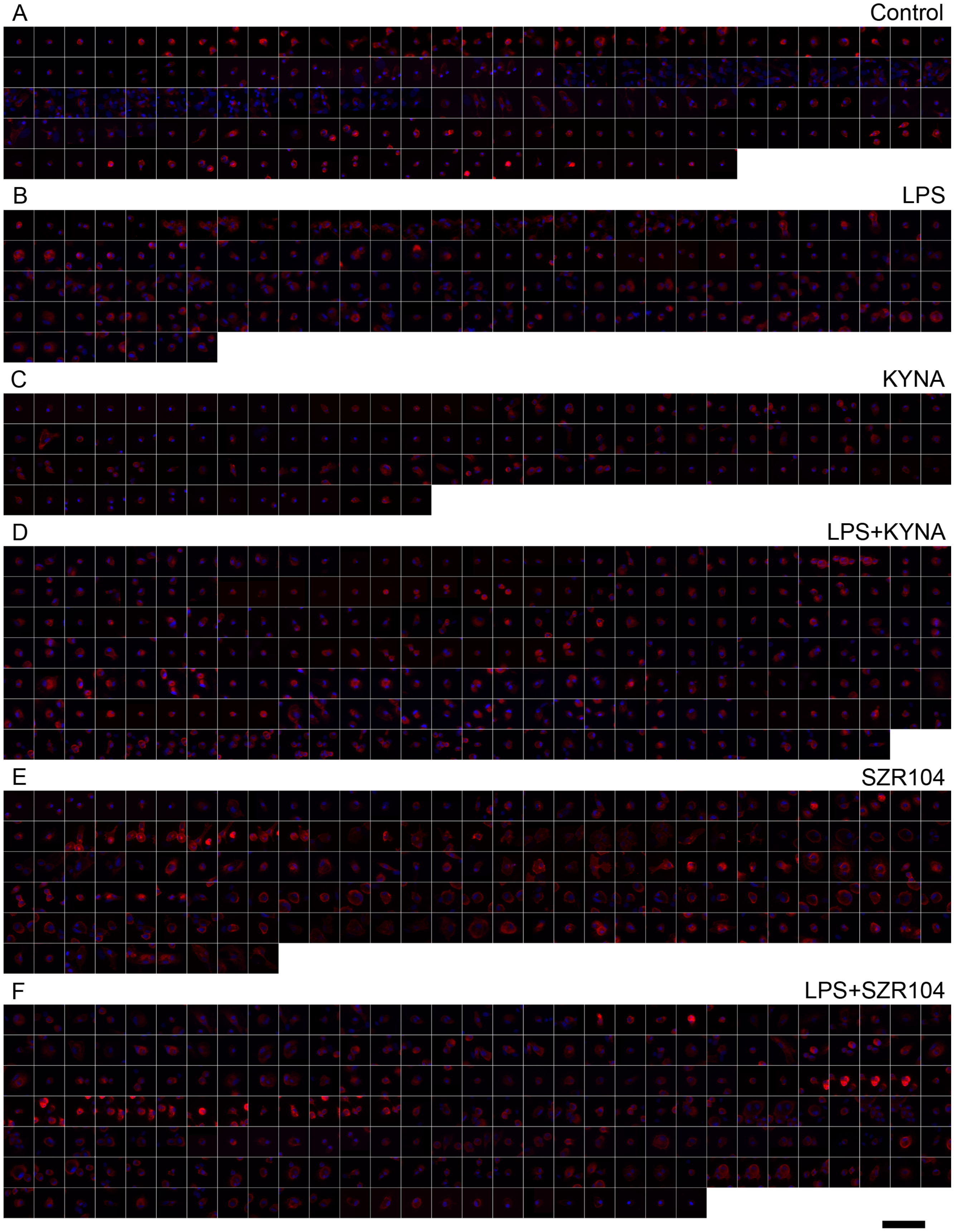

Supplementary Figure S2

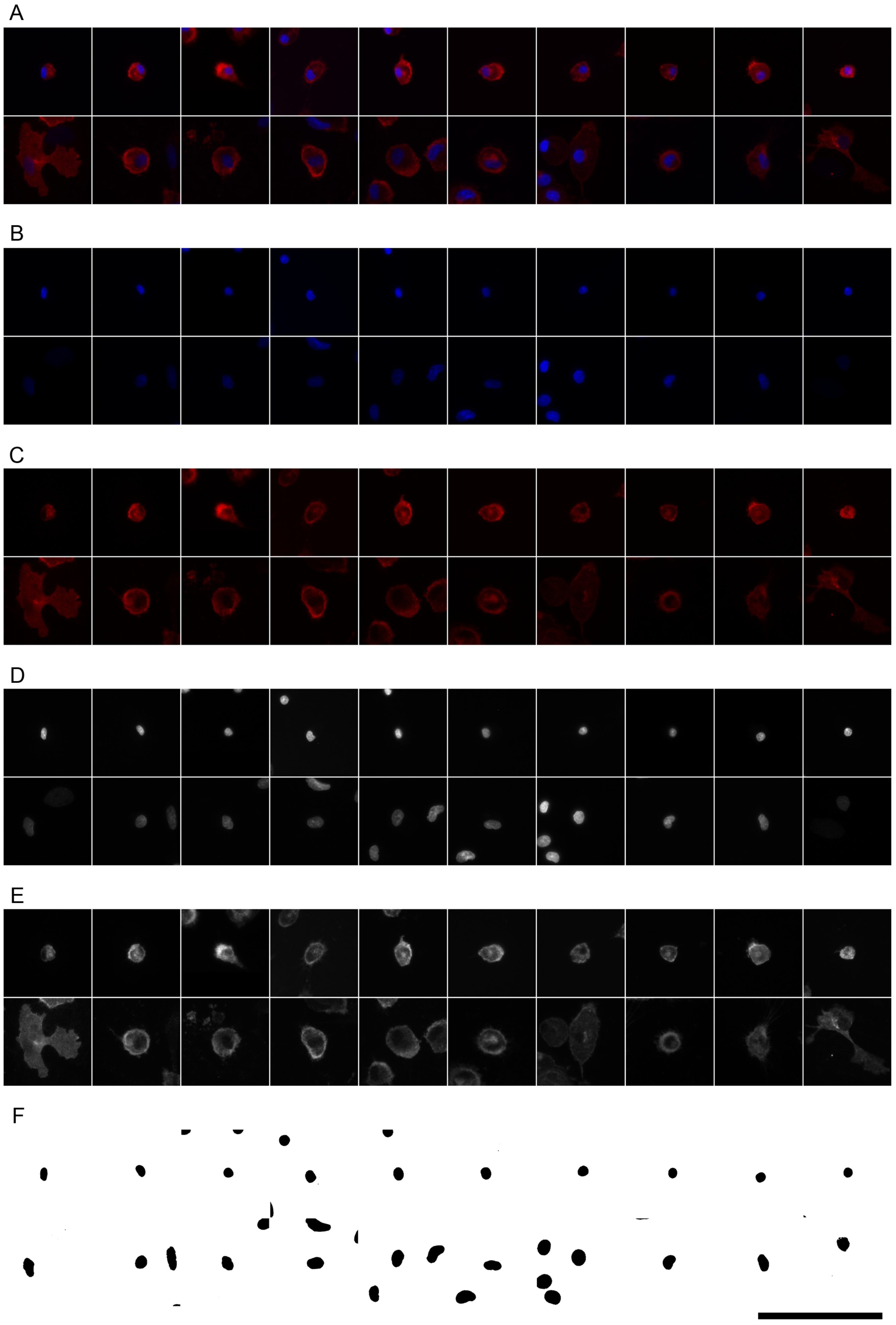

Supplementary Figure S3

G

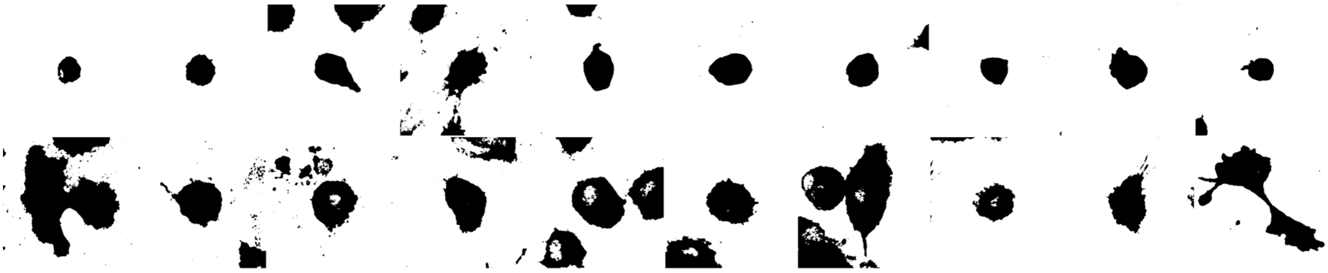

H

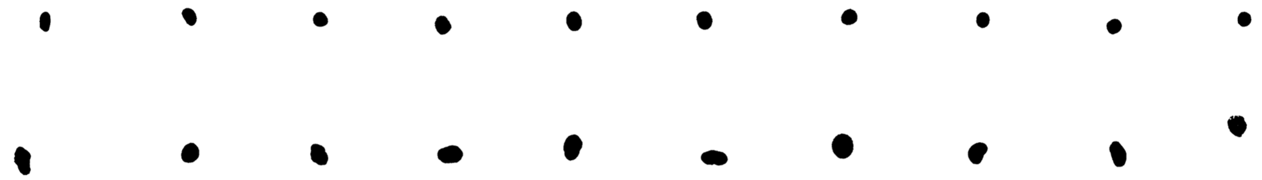

I

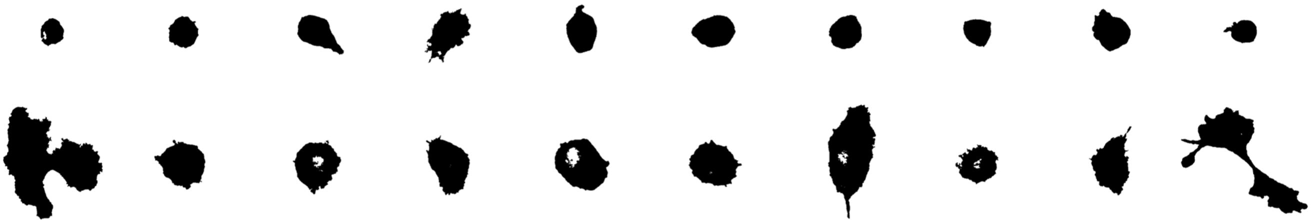

J

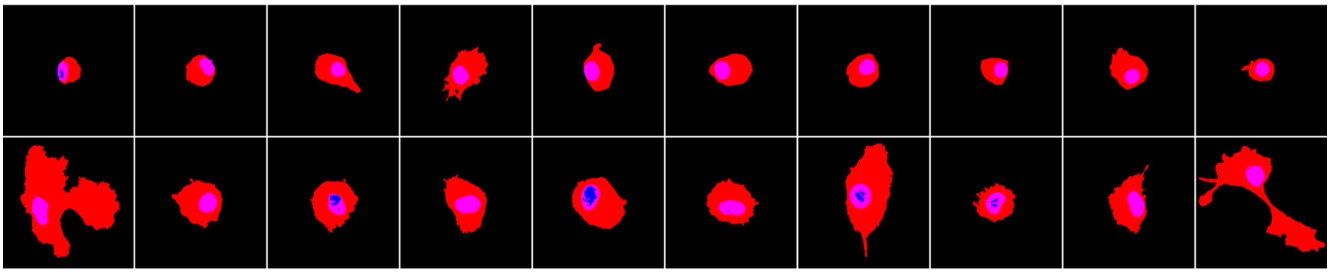

K

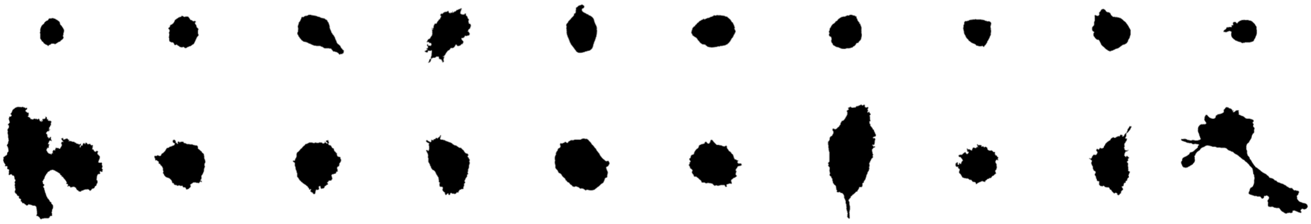

L

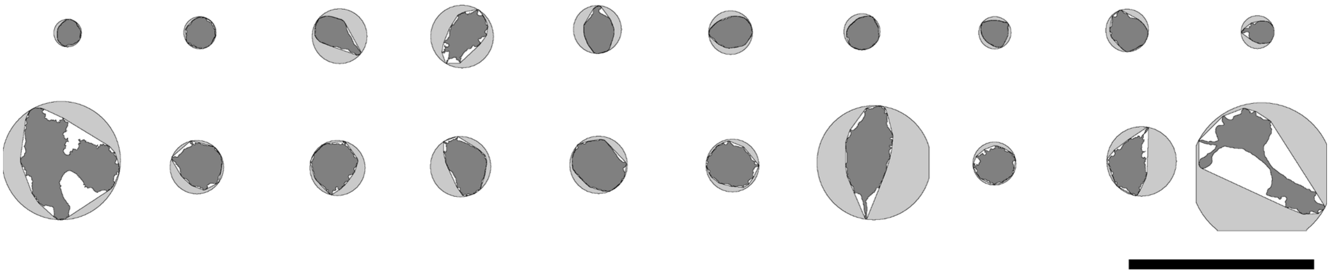

Supplementary Figure S4

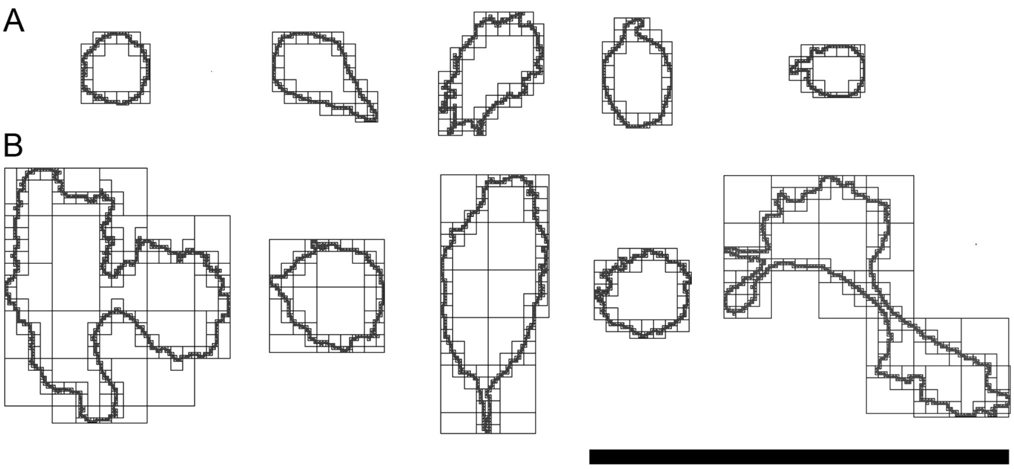

Supplementary Table S1

Num1	Group	Num2	Fractal Dim	Lacunarity	Area	Perimeter	Circularity	TI	Hull Area	Hull Perim	Hull Circula	Mean Radiu	MSACH	Diameter o	Span Ratio	Max/Min R	Density	Roughness
1	CONTROL	1	1,0328	0,1355	145,4907	46,48032	0,835294	1,197184	145,4907	43,5108	0,965718	6,994805	15,16138	15,16156	1,1329	1,4552	0,987035	1,068248
2	CONTROL	2	1,052	0,1253	177,6822	55,01613	0,707409	1,41361	177,6822	49,84472	0,898701	8,454574	19,58798	19,58798	1,5172	2,9484	0,958953	1,103751
3	CONTROL	3	1,0465	0,1137	215,3438	58,67358	0,75657	1,321755	215,3438	52,77246	0,971689	8,59117	17,62468	17,64429	1,0564	2,1974	0,962482	1,111822
4	CONTROL	4	1,0509	0,1208	129,6464	43,47145	0,860854	1,161638	129,6464	40,85926	0,975863	6,788333	13,72138	13,72186	1,0719	2,6681	0,998545	1,063932
5	CONTROL	5	1,0421	0,1055	282,0847	66,42145	0,77885	1,283944	282,0847	61,4103	0,939954	10,73936	22,47371	22,47371	1,2746	2,7407	0,969353	1,081601
6	CONTROL	6	1,0336	0,0968	264,0083	62,47039	0,845967	1,18208	264,0083	57,99989	0,986218	9,54734	19,22103	19,30571	1,0648	2,6336	0,995118	1,077078
7	CONTROL	7	1,0308	0,11	278,155	68,40975	0,721066	1,386836	278,155	63,15103	0,876467	10,95294	23,25941	23,30369	1,3411	1,7614	0,965416	1,083272
8	CONTROL	8	1,0697	0,2491	527,7338	103,277	0,518973	1,926883	527,7338	88,07491	0,854909	15,19105	33,46934	33,46934	1,3358	1,778	0,834694	1,172604
9	CONTROL	9	1,0593	0,259	440,213	91,26631	0,605744	1,650862	440,213	79,27385	0,880263	13,10087	26,82057	28,57615	1,1601	1,9754	0,91209	1,151279
10	CONTROL	10	1,0288	0,107	416,0694	87,95319	0,582787	1,715893	416,0694	79,82305	0,820576	14,21032	27,64422	29,06945	1,1917	1,379	0,862259	1,101852
11	CONTROL	11	1,0693	0,1338	365,2357	97,98298	0,402182	2,486438	365,2357	85,66621	0,625408	18,80387	37,42521	37,42521	2,4095	2,8367	0,841281	1,143776
12	CONTROL	12	1,0667	0,2302	1122,114	170,9449	0,334821	2,986671	1122,114	136,9311	0,752043	25,38869	54,26459	54,26459	2,0568	1,2545	0,69387	1,248401
13	CONTROL	13	1,066	0,0939	465,6142	97,56028	0,577507	1,731581	465,6142	79,6286	0,922779	14,06516	28,88112	28,88713	1,269	2,9805	0,939437	1,225192
14	CONTROL	14	1,0258	0,1371	199,4995	57,78174	0,732184	1,365776	199,4995	53,27417	0,88332	10,0725	20,0989	20,15603	1,3497	2,862	0,975102	1,084611
15	CONTROL	15	1,0447	0,0927	357,7851	79,8789	0,662477	1,509486	357,7851	71,15374	0,888048	11,92622	26,15715	26,22046	1,533	1,6363	0,940163	1,122624
16	CONTROL	16	1,0583	0,1179	280,1041	73,93652	0,581596	1,719407	280,1041	61,98035	0,916265	10,40894	21,2766	22,29328	1,2065	1,7962	0,903255	1,192903
17	CONTROL	17	1,0335	0,1072	288,1206	69,01507	0,736506	1,357762	288,1206	62,39911	0,92988	10,97025	21,27956	22,11129	1,1679	2,395	0,968903	1,106026
18	CONTROL	18	1,135	0,0919	542,9179	128,2404	0,372983	2,681088	542,9179	85,74874	0,927872	13,95626	30,22569	30,22887	1,2672	1,7812	0,899074	1,495537
19	CONTROL	19	1,0529	0,2694	474,165	95,78723	0,575403	1,737911	474,165	83,21007	0,860572	14,10707	29,34598	29,36789	1,0967	2,3171	0,886031	1,15115
20	CONTROL	20	1,0656	0,1742	991,8389	146,1535	0,56074	1,783357	991,8389	115,2416	0,938496	18,2911	40,42243	40,97172	1,206	1,881	0,961014	1,268236
21	CONTROL	21	1,0364	0,1079	476,8057	106,3037	0,434815	2,299827	476,8057	89,4794	0,74835	16,6805	35,27121	35,27121	2,0166	1,78	0,82007	1,188025
22	CONTROL	22	1,1212	0,2233	848,486	168,9537	0,315164	3,172956	848,486	112,6343	0,840452	19,56466	38,0252	40,16941	1,113	1,7664	0,843757	1,50002
23	CONTROL	23	1,0586	0,1288	245,6805	69,80284	0,586764	1,704262	245,6805	58,67732	0,896685	9,994025	21,82004	21,82715	1,4074	1,9414	0,92604	1,189605
24	CONTROL	24	1,0395	0,1225	214,2435	59,97039	0,703663	1,421136	214,2435	54,9291	0,892303	9,697961	21,17663	21,17663	1,4995	2,8651	0,939985	1,091778
25	CONTROL	25	1,0396	0,1069	361,4946	81,71702	0,641765	1,558203	361,4946	71,42113	0,890549	13,31459	26,58984	26,59326	1,5592	2,3823	0,943386	1,144157
26	CONTROL	26	1,0326	0,105	258,2239	64,09663	0,76589	1,30567	258,2239	58,82094	0,937869	10,78888	21,26255	21,26255	1,1647	3,261	0,969686	1,089691
27	CONTROL	27	1,0944	0,226	642,5419	121,8248	0,514105	1,945127	642,5419	92,54766	0,942714	14,7483	32,31817	32,52977	1,1367	1,5586	0,944958	1,316347
28	CONTROL	28	1,0519	0,0833	533,2352	101,142	0,618117	1,617816	533,2352	84,52268	0,937955	14,49663	30,42059	30,50809	1,2973	2,5228	0,943639	1,196626
29	CONTROL	29	1,0343	0,1086	316,8226	79,06578	0,592315	1,688291	316,8226	72,38293	0,759895	13,80569	29,89048	29,89048	1,8634	2,054	0,930046	1,092326
30	CONTROL	30	1,0504	0,0977	269,8556	66,63972	0,744045	1,344005	269,8556	61,37213	0,900324	10,08683	23,21071	23,21918	1,3688	1,8197	0,974371	1,08583
31	CONTROL	31	1,0335	0,1338	147,9742	47,14663	0,822692	1,215522	147,9742	44,53507	0,937543	7,533387	16,5569	16,5569	1,3616	2,8255	0,983429	1,058641
32	CONTROL	32	1,0292	0,1258	189,5969	54,68688	0,77315	1,293411	189,5969	50,77011	0,924325	9,135638	18,64739	18,6573	1,3001	2,2046	0,970486	1,077147
33	CONTROL	33	1,0338	0,1348	143,7931	46,43741	0,831159	1,203139	143,7931	43,29897	0,963812	6,795408	14,98725	15,00984	1,1267	1,8811	0,991911	1,072483
34	CONTROL	34	1,0406	0,1251	171,772	54,07411	0,71241	1,403685	171,772	49,02654	0,898048	9,21289	19,2684	19,2684	1,4967	3,8626	0,965044	1,102956
35	CONTROL	35	1,0933	0,1749	174,0355	73,06259	0,355372	2,813952	174,0355	49,4997	0,89257	8,611365	19,08316	19,08316	1,4977	3,2529	0,867413	1,476021
36	CONTROL	36	1,037	0,1498	227,4156	71,18564	0,51414	1,944996	227,4156	63,14188	0,716795	10,12151	27,1653	27,1653	2,2717	2,5241	0,911667	1,127392
37	CONTROL	37	1,0278	0,1188	178,311	52,05018	0,811469	1,232333	178,311	48,14652	0,966624	8,248652	15,92392	16,19002	1,0606	3,2411	0,981135	1,081079
38	CONTROL	38	1,0712	0,1251	288,2149	78,80461	0,517367	1,932864	288,2149	63,65277	0,893905	10,19223	22,21624	22,85574	1,153	1,4245	0,887107	1,238039
39	CONTROL	39	1,0897	0,1167	292,1445	83,42926	0,495597	2,01777	292,1445	64,54539	0,881205	10,07521	24,60199	24,77773	1,6045	1,8462	0,939632	1,292567
40	CONTROL	40	1,0993	0,1443	185,6987	67,43191	0,472541	2,116219	185,6987	50,36603	0,919905	9,028475	17,46071	17,56817	1,298	2,8037	0,920772	1,338837
41	CONTROL	41	1,1207	0,129	482,8102	133,4454	0,27504	3,635836	482,8102	84,5023	0,849667	14,97927	34,20883	34,20883	1,7555	2,3546	0,807267	1,579192
42	CONTROL	42	1,1163	0,1184	187,4591	62,70337	0,556646	1,796473	187,4591	50,66147	0,917826	7,939929	18,53918	18,60105	1,253	1,9378	0,929063	1,237693
43	CONTROL	43	1,1767	0,2919	620,0015	181,9968	0,174596	5,727497	620,0015	103,5147	0,727106	18,52512	42,26	42,26865	2,1357	2,6774	0,742268	1,758173
44	CONTROL	44	1,1392	0,1372	465,6456	138,8149	0,238593	4,19124	465,6456	84,71163	0,815415	15,04193	34,18126	34,18126	1,7833	2,7363	0,785714	1,638676
45	CONTROL	45	1,0658	0,0947	446,7834	96,91915	0,567888	1,760912	446,7834	79,18736	0,895354	12,7264	30,61683	30,61704	1,3597	1,8199	0,950113	1,223922
46	CONTROL	46	1,1055	0,1023	540,3715	126,3635	0,375882	2,660408	540,3715	91,72743	0,807056	17,11401	35,78268	35,78268	1,6978	2,9327	0,883879	1,377597
47	CONTROL	47	1,0749	0,094	358,6967	83,59397	0,618809	1,616007	358,6967	71,47459	0,882335	11,2511	27,93383	27,93383	1,5077	1,7589	0,959334	1,169562
48	CONTROL	48	1,0653	0,15	147,817	53,48351	0,625895	1,597712	147,817	44,01846	0,958659	7,699486	14,71204	14,89899	1,0522	2,7001	0,963845	1,215025
49	CONTROL	49	1,1018	0,2949	441,8792	113,203	0,366999	2,724804	441,8792	81,75472	0,830783	13,81988	33,19346	33,19346	1,6406	1,8088	0,846969	1,384666
50	CONTROL	50	1,0562	0,1359	163,5041	55,16312	0,643925	1,552976	163,5041	49,27493	0,846226	8,293564	19,9608	19,9608	1,6791	3,1491	0,953663	1,119497
51	CONTROL	51	1,0723	0,1112	298,5891	78,8477	0,536945	1,862387	298,5891	65,62913	0,871144	11,73316	24,8341	24,84188	1,4073	1,8436	0,889661	1,201413
52	CONTROL	52	1,0412	0,1238	401,2625	106,2534	0,368673	2,712429	401,2625	91,64051	0,600431	16,1719	41,10303	41,10303	2,9021	3,0165	0,825447	1,159458
53	CONTROL	53	1,0616	0,1246	270,0443	76,05674	0,528587	1,891834	270,0443	62,5711	0,866756	10,68035	22,5533	22,77002	1,1492	2,4878	0,901048	1,215525
54	CONTROL	54	1,1078	0,1397	334,1758	101,9447	0,345454	2,89474	334,1758	68,07504	0,906168	12,39725	25,3453	25,3453	1,4392	1,9019	0,854939	1,497534
55	CONTROL	55	1,0464	0,1487	136,2482	56,92872	0,503795	1,984935	136,2482	51,27066	0,651332	9,180372	22,14681	22,14681	2,8969	3,2807	0,953623	1,110357
56	CONTROL	56	1,0659	0,1281	277,4634	77,75851	0,505377	1,97872	277,4634	62,42521	0,894736	10,40613	22,47021	22,5394	1,4749	1,487	0,876388	1,245627
57	CONTROL	57	1,1247	0,3087	501,8296	138,306	0,267943	3,732132	501,8296	102,2825	0,602786	19,58702	45,92027	45,92027	2,6923	2,9532	0,812754	1,352196
58	CONTROL	58	1,0512	0,1175	257,8781	69,1367	0,644409	1,55181	257,8781	59,72168	0,908573	10,32014	21,87401	21,90876	1,4055	3,5319	0,950506	1,157648
59	CONTROL	59	1,1206	0,1309	343,8899	100,5335	0,367298	2,722583	343,8899	68,0125	0,934225	11,22649	23,29791	23,52207	1,2438	2,1806	0,859036	1,478162
60	CONTROL	60	1,0467	0,1117	243,9515	64,21826	0,714521	1,39954	243,9515	57,17349	0,937829	9,841879	20,2353	20,25085	1,121	2,44	0,961211	1,123217
61	CONTROL	61	1,1344	0,15	552,2232	135,6546	0,222726	4,489828	552,2232	103,3757	0,649363	20,06002	43,79433	43,79433	2,0315	1,7302	0,59063	1,312249
62	CONTROL	62	1,0947	0,2929	444,3627	113,8619	0,36767	2,719832	444,3627	90,27869	0,685136	16,45387	39,7531	39,7531	2,3175	3,7132	0,853626	1,261227
63	CONTROL	63	1,0955	0,2483	789,9816	155,1521	0,336721	2,969813	789,9816	108,1835	0,848212	18,19908	41,00156	41,00652	1,6842	1,9775	0,816507	1,434157
64	CONTROL	64	1,1454	0,3086	529,5886	141,7734	0,256648	3,896382	529,5886	87,22562	0,874702	14,31645	30,63684	31,71082	1,2609	1,6311	0,775139	1,625364
65	CONTROL	65	1,1411	0,3226	676,9969	162,8392	0,222087	4,502734	676,9969	109,3288	0,711749	17,48704	46,80986	46,80986	2,0322	2,4608	0,692222	1,489444
66	CONTROL	66	1,1232	0,2927	529,3999	123,1647	0,33381	2,995719	529,3999	86,66583	0,885722	15,06223	31,82117	31,82422	1,2977	1,7421	0,761164	1,421145
67	CONTROL	67	1,1743	0,2704	1153,08	240,1541	0,147563	6,776766	1153,08	143,7145	0,701565	25,4956	59,2647	59,2647	1,9388	1,9006	0,587339	1,67105
68	CONTROL	68	1,1117	0,2957	490,4494	117,6661	0,350614	2,852138	490,4494	89,8478	0,763464	14,57067	36,59404	36,59404	1,7714	2,0326	0,787642	1,309616
69	CONTROL	69	1,0461	0,1545	205,9127	67,39929	0,490478	2,038826	205,9127	57,48174	0,783128	9,788404	23,43715	23,43715	2,1216	2,1932	0,861069	1,172534
70	CONTROL	70	1,0377	0,1338	200,0025	57,54894	0,732514	1,365162	200,0025	52,4145	0,914833	8,875674	19,87399	19,87399	1,342	3,6821	0,965262	1,097958
71	CONTROL	71	1,1091	0,1447	377,4018	107,3676	0,307944	3,247345	377,4018	75,37335	0,834791	13,37945	29,3433	29,3433	1,7444	2,2014	0,748521	1,424476
72	CONTROL	72	1,0475	0,1274	287,3661	82,30762	0,456772	2,189277	287,3661	71,19977	0,712341	12,81002	30,05881	30,05881	2,3859	1,8449	0,856908	1,15601
73	CONTROL	73	1,0462	0,1441	279,3811	84,72908	0,40875	2,446481	279,3811	75,50163	0,615877	13,76429	32,68814	32,71392	2,6426	2,3176	0,835828	1,122215
74	CONTROL	74	1,0788	0,1058	371,1144	92,36277	0,510084	1,96046	371,1144	71,19667	0,920021	11,49569	23,88885	24,93622	1,046	1,3792	0,933079	1,297291
75	CONTROL	75	1,0784	0,1251	454,7369	121,1156	0,31318	3,193056	454,7369	81,85441	0,852875	14,06282	31,40752	31,40752	1,5257	2,3707	0,803941	1,479647
76	CONTROL	76	1,1377	0,1685	310,7238	109,0768	0,243317	4,109867	310,7238	71,76647	0,758125	12,8184	29,78988	29,78988	2,264	2,6906	0,7414	1,519885
77	CONTROL	77	1,1567	0,2671	510,1919	140,398	0,281202	3,556168	510,1919	82,4066	0,944102	13,21429	28,76333	28,76333	1,1347	1,4465	0,864563	1,703723
78	CONTROL	78	1,2102	0,3125	1037,423	262,95	0,102832	9,724579	1037,423	143,3709	0,634225	25,43069	61,228	61,228	2,818	2,3656	0,545394	1,834054
79	CONTROL	79	1,1509	0,3688	816,3259	211,5764	0,138553	7,217447	816,3259	138,0695	0,538118	26,36743	63,41794	63,41794	3,4001	2,3842	0,604614	1,53239
80	CONTROL	80	1,1056	0,28	528,0167	122,5562	0,359305	2,783151	528,0167	89,9091	0,820824	15,72406	32,46424	33,1927	1,5202	1,6828	0,813348	1,363112
81	CONTROL	81	1,0861	0,2161	808,0894	142,1532	0,446161	2,241343	808,0894	106,7325	0,891406	17,70241	38,07931	38,31562	1,4499	1,6352	0,887843	1,331864
82	CONTROL	82	1,0493	0,1234	186,0759	55,34255	0,75223	1,329381	186,0759	49,2608	0,9636	8,572074	17,21319	17,21319	1,2559	3,3315	0,985302	1,12346
83	CONTROL	83	1,0796	0,1561	621,0704	152,9385	0,208214	4,802746	621,0704	112,9952	0,611267	20,82152	51,63199	51,63199	2,8524	2,8842	0,624013	1,353496
84	CONTROL	84	1,1528	0,3175	328,4857	119,5578	0,234447	4,265354	328,4857	86,47309	0,552032	16,9352	39,49486	39,49486	3,3764	3,3153	0,811848	1,382601
85	CONTROL	85	1,0777	0,139	407,9272	112,1601	0,292992	3,41306	407,9272	81,97745	0,762787	14,0059	32,31527	32,31527	1,4735	2,4203	0,71902	1,368182
86	CONTROL	86	1,1628	0,3055	708,8426	185,0014	0,188997	5,291077	708,8426	113,4596	0,691954	20,14516	45,26835	45,43239	1,9982	2,5463	0,726184	1,630549
87	CONTROL	87	1,0269	0,1039	284,6625	66,94078	0,777921	1,285478	284,6625	62,43213	0,917748	10,10862	22,76833	22,86798	1,378	1,6894	0,974489	1,072217
88	CONTROL	88	1,0513	0,09	408,273	87,72021	0,63276	1,580379	408,273	77,58293	0,852369	14,76018	30,49697	30,49754	1,6572	3,0788	0,949026	1,130664
89	CONTROL	89	1,0199	0,0881	347,4737	73,63546	0,782641	1,277726	347,4737	67,57544	0,956211	11,57872	24,17402	24,17605	1,181	2,9968	0,971863	1,089678
90	CONTROL	90	1,0513	0,09	408,273	87,72021	0,63276	1,580379	408,273	77,58293	0,852369	14,76018	30,49697	30,49754	1,6572	3,0788	0,949026	1,130664
91	CONTROL	91	1,1275	0,1397	296,6086	95,41472	0,330612	3,024694	296,6086	63,93959	0,911703	10,98475	24,03644	24,03644	1,3203	2,3536	0,807525	1,492263
92	CONTROL	92	1,0559	0,1455	194,1867	66,93032	0,499492	2,002033	194,1867	58,91475	0,703041	10,00174	25,04271	25,04271	2,4609	2,4445	0,91695	1,136054
93	CONTROL	93	1,0681	0,1291	139,2976	47,94184	0,748531	1,335949	139,2976	42,45025	0,971388	7,567961	14,23087	14,3666	1,1053	2,7587	0,982848	1,129365
94	CONTROL	94	1,0851	0,2441	1259,588	205,548	0,251008	3,983933	1259,588	151,4596	0,689992	26,60309	63,11707	63,11707	2,1484	2,6501	0,670003	1,357114
95	CONTROL	95	1,1402	0,2734	465,0797	119,9837	0,288245	3,469272	465,0797	84,5652	0,817247	15,61757	32,74195	32,74195	1,6889	1,5133	0,710018	1,418831
96	CONTROL	96	1,0624	0,1258	255,7718	71,62642	0,571436	1,749979	255,7718	59,63693	0,903715	9,898475	22,83037	22,83037	1,3851	1,8794	0,912119	1,201041
97	CONTROL	97	1,1506	0,283	822,6133	177,5043	0,211444	4,729396	822,6133	110,5216	0,846274	18,89624	39,26135	40,08674	1,1601	1,308	0,644476	1,606059
98	CONTROL	98	1,0637	0,1501	127,3515	48,11383	0,666396	1,500609	127,3515	40,97977	0,95296	6,727624	13,9925	14,01211	1,1425	1,4842	0,96396	1,174087
99	CONTROL	99	1,0458	0,1357	194,8783	60,2211	0,644004	1,552786	194,8783	54,34899	0,829068	8,66656	19,01218	20,28012	1,1273	2,2588	0,953702	1,108045
100	CONTROL	100	1,0908	0,3365	501,8296	128,3296	0,26886	3,719412	501,8296	110,9342	0,512431	19,66977	51,79156	51,79156	3,493	3,3315	0,702124	1,156809
101	CONTROL	101	1,1397	0,1113	601,8623	160,2278	0,255852	3,908506	601,8623	101,9792	0,72725	17,05069	43,05738	43,05738	2,4135	2,8481	0,868477	1,571182
102	CONTROL	102	1,0494	0,1375	227,3213	69,80284	0,54055	1,849969	227,3213	60,24918	0,78695	9,626489	23,78268	23,79993	1,5364	2,013	0,922002	1,158569
103	CONTROL	103	1,1598	0,2316	562,1573	146,5807	0,301685	3,314717	562,1573	85,26222	0,971749	13,64262	29,0764	29,07823	1,114	1,9157	0,917571	1,719175
104	CONTROL	104	1,0881	0,1037	341,4064	87,8672	0,526876	1,89798	341,4064	66,68894	0,964658	10,87275	22,5289	22,59695	1,1306	1,9333	0,948158	1,317568
105	CONTROL	105	1,0443	0,0957	272,5278	66,3711	0,76398	1,308935	272,5278	59,94965	0,952899	11,19828	21,05082	21,05307	1,1521	4,6238	0,982697	1,107114

106	CONTROL	106	1,0677	0,1058	226,3782	61,76117	0,722999	1,383127	226,3782	54,44135	0,959812	8,897447	18,05466	18,33727	1,0963	2,0083	0,969449	1,134453
107	CONTROL	107	1,0355	0,1011	307,2343	73,99734	0,669381	1,493917	307,2343	66,1761	0,881611	13,1964	26,1367	26,1367	1,5763	5,1087	0,94935	1,118188
108	CONTROL	108	1,0597	0,1003	347,9138	85,51702	0,5531	1,807991	347,9138	72,89404	0,822805	14,65745	28,28557	28,28676	1,9284	2,9648	0,925183	1,173169
109	CONTROL	109	1,0273	0,0972	308,4603	71,03883	0,756513	1,321854	308,4603	64,11307	0,943009	11,09683	23,77541	23,77541	1,3552	4,0693	0,984916	1,108024
110	CONTROL	110	1,0743	0,1286	265,203	78,51082	0,486574	2,055188	265,203	63,77668	0,81934	10,66523	25,91626	25,91626	1,5995	2,8253	0,899953	1,231027
111	CONTROL	111	1,0401	0,1002	250,7734	62,21968	0,80504	1,242174	250,7734	56,92239	0,972578	9,784397	19,82727	19,82727	1,1282	3,2232	0,988968	1,093062
112	CONTROL	112	1,0238	0,106	225,8438	57,18688	0,867086	1,153289	225,8438	53,8673	0,978064	9,522039	17,56035	18,00608	1,0384	4,7586	0,999165	1,061625
113	CONTROL	113	1,0337	0,1195	175,8589	52,58741	0,794545	1,258581	175,8589	48,89461	0,924383	7,888138	17,02496	17,57012	1,1001	3,0019	0,99428	1,075526
114	CONTROL	114	1,0325	0,1227	205,2211	59,85904	0,691509	1,446113	205,2211	54,75218	0,860258	9,104858	21,50074	21,50074	1,6544	2,4835	0,960784	1,093272
115	CONTROL	115	1,0292	0,1055	291,7987	68,54911	0,746469	1,339641	291,7987	62,01179	0,953552	10,45179	22,24878	22,24878	1,2136	2,679	0,956583	1,105421
116	CONTROL	116	1,0524	0,1521	181,5175	59,77004	0,595594	1,678995	181,5175	53,78809	0,788417	9,985426	21,83805	21,8402	1,7683	2,95	0,932802	1,111213
117	CONTROL	117	1,0353	0,1404	169,2256	52,69858	0,726757	1,375975	169,2256	47,65631	0,936343	9,63117	16,63362	16,64511	1,1088	3,6821	0,949099	1,105805
118	CONTROL	118	1,0205	0,1277	181,6433	53,24309	0,795582	1,256942	181,6433	49,56809	0,929019	7,934344	17,56035	17,57727	1,1187	2,2304	0,988058	1,07414
119	CONTROL	119	1,0415	0,1344	142,5356	47,45816	0,777899	1,285514	142,5356	43,58995	0,942671	8,883422	14,97887	15,09621	1,1308	3,5269	0,978165	1,088741
120	CONTROL	120	1,0195	0,1411	184,3469	55,79362	0,694937	1,438979	184,3469	51,69642	0,866811	9,761738	19,79234	19,79234	1,4504	2,7786	0,933834	1,079255
121	CONTROL	121	1,0355	0,1155	214,4321	59,6766	0,743998	1,34409	214,4321	55,97789	0,859936	9,141188	22,02082	22,02177	1,6268	2,3777	0,983287	1,066074
122	CONTROL	122	1,0523	0,1304	303,5562	84,39965	0,477056	2,096189	303,5562	72,73046	0,721134	11,12418	29,61842	29,66637	2,1319	3,9421	0,890845	1,160444
123	CONTROL	123	1,0172	0,1076	239,3931	59,06383	0,856903	1,166993	239,3931	55,47188	0,977632	9,049291	18,07119	18,47855	1,0205	1,9704	0,993697	1,064753
124	CONTROL	124	1,1375	0,3388	840,7839	171,8619	0,195716	5,109454	840,7839	124,4489	0,6822	22,41977	49,7327	49,83746	1,4403	2,0134	0,54713	1,380984
125	CONTROL	125	1,0462	0,1099	192,9292	57,62748	0,710533	1,407393	192,9292	52,47	0,880613	9,282553	19,88426	19,90496	1,53	3,7513	0,973277	1,098294
126	CONTROL	126	1,0215	0,1457	145,4278	47,40461	0,798643	1,252123	145,4278	43,81493	0,951947	7,307411	14,98725	15,09413	1,1159	2,4472	0,982058	1,081928
127	CONTROL	127	1,0382	0,126	178,3424	53,01755	0,784516	1,274671	178,3424	48,81349	0,940555	8,780195	16,91195	16,94757	1,1657	2,4212	0,983959	1,086125
128	CONTROL	128	1,0592	0,1135	281,4245	72,22429	0,626311	1,59665	281,4245	61,3155	0,940656	10,92362	21,80851	21,85927	1,1643	2,4291	0,923816	1,177913
129	CONTROL	129	1,0541	0,1062	326,1594	78,46773	0,57796	1,730225	326,1594	69,67278	0,844332	12,41888	27,22945	27,22945	1,6007	1,9991	0,868241	1,126232
130	CONTROL	130	1,0145	0,1035	323,0471	72,26738	0,748333	1,336304	323,0471	65,96062	0,933052	12,44895	23,09053	23,36518	1,256	3,5964	0,962729	1,095614
131	CONTROL	131	1,0843	0,1093	332,101	89,41472	0,488735	2,046098	332,101	65,76833	0,964819	11,44878	22,34324	22,38161	1,1285	1,9916	0,936293	1,359541
132	CONTROL	132	1,0318	0,1007	253,8856	63,55213	0,774767	1,290711	253,8856	58,06695	0,964214	11,4205	20,71131	20,71131	1,1432	4,9454	0,980807	1,094463
133	CONTROL	133	1,0556	0,1093	217,7959	59,00301	0,765053	1,307099	217,7959	53,3997	0,959802	8,704699	17,9034	18,18941	1,131	1,9695	0,973152	1,104932
134	CONTROL	134	1,0243	0,1141	289,3152	71,8445	0,659968	1,515226	289,3152	64,84571	0,864606	13,65099	25,79711	25,79711	1,6447	4,9701	0,936977	1,10793
135	CONTROL	135	1,0513	0,1135	171,9292	55,17358	0,688713	1,451984	171,9292	51,53099	0,813621	9,627411	20,88589	20,88589	1,5202	3,2175	0,970378	1,070687
136	CONTROL	136	1,0575	0,1251	146,9996	47,56206	0,800699	1,248909	146,9996	43,97957	0,955045	7,812748	15,34277	15,53855	1,1416	3,5161	0,980539	1,081458
137	CONTROL	137	1,036	0,1335	124,7422	42,71915	0,860701	1,161843	124,7422	40,21665	0,969195	7,546348	14,03848	14,05938	1,1818	7,2839	1,002016	1,062225
138	CONTROL	138	1,0262	0,1526	148,6972	52,84557	0,649727	1,539109	148,6972	48,858	0,782783	7,894539	20,25083	20,2606	1,8037	4,1717	0,971036	1,081616
139	CONTROL	139	1,0458	0,1394	123,1704	43,57535	0,796419	1,255621	123,1704	40,27771	0,954083	7,101312	13,87064	14,00191	1,0792	3,1008	0,977029	1,081873
140	CONTROL	140	1,0445	0,1227	152,4383	48,37518	0,806927	1,23927	152,4383	44,80918	0,954046	7,762908	15,07092	15,60817	1,1183	3,5171	0,98577	1,079582
141	CONTROL	141	1,0439	0,1084	323,0471	77,6977	0,635476	1,573624	323,0471	66,35275	0,922057	11,51663	24,85371	24,86768	1,4712	2,1891	0,945018	1,170979
142	CONTROL	142	1,068	0,1618	254,5458	81,38333	0,38406	2,603762	254,5458	72,68534	0,605455	13,62633	28,0125	28,95422	1,71	2,0064	0,795233	1,119666
143	CONTROL	143	1,0639	0,1197	345,3046	88,17872	0,472251	2,117517	345,3046	74,70385	0,777546	12,2448	27,67434	28,46846	1,581	2,241	0,846231	1,180377
144	CONTROL	144	1,0455	0,1088	218,3618	60,83688	0,722614	1,383866	218,3618	55,32436	0,896506	10,2956	20,90168	20,91674	1,437	3,3745	0,974662	1,09964
145	CONTROL	145	1,0438	0,126	198,525	59,47926	0,664747	1,504333	198,525	54,04917	0,853978	8,470691	22,04651	22,04651	1,459	2,4574	0,942676	1,100466
146	CONTROL	146	1,1309	0,2049	218,739	85,55691	0,267792	3,73424	218,739	60,23826	0,757514	10,36142	22,09137	22,98525	1,473	2,8218	0,713136	1,420308
147	CONTROL	147	1,0571	0,1399	161,1463	55,34557	0,615569	1,624512	161,1463	47,7361	0,888661	9,607589	17,95076	18,03183	1,4017	5,3519	0,931135	1,159407
148	CONTROL	148	1,0491	0,1482	142,9443	50,22376	0,685033	1,459783	142,9443	46,05934	0,846722	7,704113	18,07642	18,07642	1,4509	4,2469	0,961953	1,090414
149	LPS	1	1,0272	0,1038	217,4501	56,80709	0,851664	1,174172	217,4501	53,55699	0,952658	9,732323	19,59842	19,59858	1,2762	6,4761	1,005783	1,060685
150	LPS	2	1,0207	0,1213	205,2839	56,64238	0,788656	1,26798	205,2839	52,51959	0,935237	8,881702	18,95174	18,95215	1,2684	2,7435	0,980858	1,0785
151	LPS	3	1,0262	0,1295	149,3889	46,68067	0,859684	1,163219	149,3889	44,0708	0,966553	7,944787	15,1966	15,24468	1,1956	5,0389	0,997896	1,05922
152	LPS	4	1,031	0,1207	161,7751	49,41365	0,836142	1,19597	161,7751	46,42381	0,943277	7,404273	16,88126	16,88126	1,274	4,6608	1,004275	1,064403
153	LPS	5	1,0354	0,1302	171,3319	51,42713	0,79585	1,256519	171,3319	47,58459	0,950857	8,923635	16,00172	16,13527	1,0934	3,2577	0,977615	1,080752
154	LPS	6	1,0548	0,1191	161,0834	51,10816	0,754241	1,325836	161,0834	48,22092	0,870541	8,762553	17,22142	17,68865	1,2985	3,8388	0,973263	1,059875
155	LPS	7	1,0678	0,2404	545,59	113,4034	0,4793	2,086376	545,59	97,1209	0,72686	18,05911	40,81867	40,81867	2,1809	2,9083	0,899049	1,167651
156	LPS	8	1,0872	0,1449	1180,304	164,6793	0,491873	2,033044	1180,304	130,2939	0,873686	21,98535	47,99695	47,99695	1,3655	1,7257	0,899347	1,263906
157	LPS	9	1,094	0,2613	608,6842	130,1383	0,328501	3,044134	608,6842	100,8286	0,752375	18,64755	41,71908	41,71908	2,1122	1,832	0,727353	1,290689
158	LPS	10	1,0435	0,112	223,9575	59,29681	0,783445	1,276414	223,9575	53,97445	0,966048	9,16922	18,63812	18,67387	1,1892	3,0599	0,978804	1,098609
159	LPS	11	1,042	0,1072	207,736	62,82074	0,610025	1,639277	207,736	58,47546	0,763438	10,65667	22,46741	22,50828	2,0364	1,5186	0,922215	1,07431
160	LPS	12	1,0841	0,285	532,1978	113,8619	0,425566	2,349813	532,1978	86,19012	0,900259	14,78278	31,63888	31,80551	1,3688	2,1839	0,824975	1,321055
161	LPS	13	1,0304	0,0879	1189,295	190,1798	0,304651	3,282447	1189,295	154,9602	0,622385	28,28872	66,92294	66,93472	3,2034	2,7564	0,737279	1,227282
162	LPS	14	1,0397	0,1456	180,4487	70,86986	0,43284	2,310321	180,4487	62,77351	0,575454	10,88534	29,11745	29,11745	3,228	7,905	0,958711	1,128977
163	LPS	15	1,0754	0,0982	373,535	88,55851	0,546639	1,829361	373,535	71,78674	0,910862	13,19672	25,73855	25,73855	1,2831	2,5516	0,913314	1,233633
164	LPS	16	1,0471	0,1012	360,5515	79,91152	0,678083	1,474746	360,5515	70,39021	0,914434	11,42746	26,56323	26,56323	1,3914	1,6761	0,955707	1,135265
165	LPS	17	1,0366	0,1122	292,396	72,27482	0,663552	1,507041	292,396	64,16112	0,892559	10,99693	24,30113	24,30113	1,2767	2,6291	0,943339	1,126458
166	LPS	18	1,08	0,1441	245,4291	79,30603	0,404631	2,471385	245,4291	59,89186	0,859805	10,66768	20,88665	21,05229	1,1639	1,4988	0,825157	1,324154
167	LPS	19	1,0638	0,1221	283,908	79,32394	0,519781	1,923886	283,908	61,76711	0,935131	10,25424	21,86323	21,86323	1,1457	2,2628	0,916731	1,284242
168	LPS	20	1,1169	0,2923	709,157	144,3553	0,269541	3,710016	709,157	106,5918	0,784339	19,51491	36,97521	39,25817	1,2024	1,2002	0,630286	1,354281
169	LPS	21	1,0872	0,114	340,0231	90,51099	0,473062	2,11389	340,0231	68,46504	0,91155	11,40117	25,72633	25,7291	1,456	1,5929	0,90699	1,322003
170	LPS	22	1,0493	0,244	506,3251	97,64929	0,567257	1,762869	506,3251	86,12904	0,857709	14,41537	31,29521	31,66023	1,325	1,5589	0,850118	1,133756
171	LPS	23	1,0453	0,1125	371,0829	92,56312	0,487038	2,053229	371,0829	73,29612	0,867998	13,39443	28,08199	28,08424	1,498	2,8579	0,894866	1,262865
172	LPS	24	1,0938	0,1167	393,026	113,0236	0,333745	2,996297	393,026	72,66543	0,935352	11,98796	25,95989	26,22622	1,2846	1,9084	0,863222	1,555397
173	LPS	25	1,0657	0,1259	293,905	78,68298	0,554319	1,804016	293,905	63,40885	0,918579	10,43941	23,72511	23,72544	1,3327	1,7695	0,92919	1,240883
174	LPS	26	1,0687	0,2095	493,153	95,80798	0,62908	1,589622	493,153	80,76879	0,949957	14,70149	26,7519	27,24915	1,0147	3,4606	0,931791	1,1862
175	LPS	27	1,0723	0,1131	241,9081	66,36809	0,658218	1,519254	241,9081	55,99592	0,969498	9,267872	18,77759	18,83945	1,0883	1,9371	0,953736	1,185231
176	LPS	28	1,1049	0,2534	473,1905	109,6138	0,450938	2,217602	473,1905	80,26254	0,923038	12,90429	27,98723	28,64959	1,1915	1,4146	0,911175	1,365691
177	LPS	29	1,0998	0,2135	653,3877	127,828	0,458393	2,181536	653,3877	93,83358	0,932532	15,75775	33,87638	33,97676	1,3496	1,954	0,91224	1,362284
178	LPS	30	1,04	0,1165	226,6297	61,13794	0,738343	1,354384	226,6297	54,28284	0,966499	8,845142	18,40729	18,66798	1,1004	3,0757	0,969066	1,126285
179	LPS	31	1,1008	0,1326	296,0741	84,6578	0,461363	2,167491	296,0741	62,81388	0,942972	10,74447	21,9264	21,9264	1,1123	1,817	0,888724	1,347756
180	LPS	32	1,0765	0,2676	470,8327	106,0321	0,486626	2,054967	470,8327	90,63977	0,720176	15,01809	38,93812	38,93812	2,1637	2,9017	0,924685	1,169819
181	LPS	33	1,0298	0,117	355,9932	86,85372	0,560348	1,784604	355,9932	77,23328	0,749967	13,05656	31,84587	31,84587	2,147	2,4352	0,944896	1,124563
182	LPS	34	1,0358	0,101	204,3723	59,03121	0,729065	1,371619	204,3723	55,52369	0,833057	8,790496	20,14188	20,20819	1,5451	1,8555	0,989232	1,063172
183	LPS	35	1,0361	0,1503	121,6299	48,17784	0,640799	1,560552	121,6299	45,59771	0,73513	8,040496	18,48144	18,48144	1,9447	1,6111	0,97312	1,056585
184	LPS	36	1,0531	0,1604	116,8515	48,39291	0,585352	1,708373	116,8515	44,48188	0,742126	7,76117	18,2495	18,2495	2,258	2,0119	0,933549	1,087924
185	LPS	37	1,0403	0,14	140,6179	49,37801	0,707404	1,413618	140,6179	45,88243	0,839378	7,747748	18,19342	18,19342	1,752	2,6577	0,976079	1,076186
186	LPS	38	1,0704	0,1174	185,2585	56,25213	0,715615	1,397399	185,2585	49,51826	0,949417	7,809309	18,08163	18,08163	1,2903	1,3946	0,972679	1,135988
187	LPS	39	1,0338	0,1037	384,5066	85,44574	0,619603	1,613936	384,5066	75,72087	0,842718	13,29941	29,62002	29,62002	1,9002	1,8573	0,936228	1,128431
188	LPS	40	1,0347	0,1046	309,372	73,72145	0,685013	1,459826	309,372	66,53965	0,878071	11,03548	23,415	24,12043	1,2857	2,2951	0,957626	1,107933
189	LPS	41	1,0571	0,1087	250,0503	64,89929	0,725959	1,377487	250,0503	56,72147	0,976657	9,18133	18,55275	19,04936	1,0213	1,573	0,973095	1,144175
190	LPS	42	1,0623	0,1149	363,8839	87,85674	0,531557	1,881267	363,8839	71,91901	0,884066	13,17243	25,22596	25,7558	1,1126	2,5665	0,897279	1,221607
191	LPS	43	1,0636	0,2683	494,8506	102,4356	0,551665	1,812693	494,8506	83,67878	0,888082	14,42316	29,22683	29,75426	1,2407	1,7311	0,930881	1,224153
192	LPS	44	1,0814	0,2095	592,2426	110,9819	0,561896	1,779688	592,2426	88,66833	0,946612	14,33592	31,04862	31,54849	1,2535	1,7468	0,929933	1,251652
193	LPS	45	1,1713	0,2183	1173,671	244,764	0,203831	4,906036	1173,671	124,8966	0,945485	20,8964	42,05679	43,33516	1,1206	1,4964	0,827958	1,959734
194	LPS	46	1,1236	0,2471	590,4507	133,4023	0,355066	2,816379	590,4507	89,23518	0,931796	15,44647	32,19929	32,19968	1,2043	1,9234	0,851613	1,494952
195	LPS	47	1,0534	0,1199	237,8527	65,92004	0,667105	1,499015	237,8527	57,11463	0,916269	9,464043	19,93794	20,1694	1,2997	2,1913	0,969865	1,154171
196	LPS	48	1,0434	0,1251	165,8304	50,51011	0,798376	1,252542	165,8304	46,62846	0,958455	7,763174	15,77216	15,96246	1,1352	1,6939	0,977441	1,083246
197	LPS	49	1,0531	0,0973	204,7181	56,53848	0,794894	1,25803	204,7181	51,60904	0,96586	8,337784	17,13631	17,31605	1,0495	1,3449	0,987715	1,095515
198	LPS	50	1,0061	0,1326	184,0639	53,47606	0,791704	1,263098	184,0639	49,33333	0,95038	8,233493	17,3306	17,33064	1,1775	1,7985	0,978822	1,083974
199	LPS	51	1,0434	0,1384	214,1178	62,64255	0,626286	1,596715	214,1178	54,01628	0,922174	8,623121	19,35794	19,41879	1,2477	1,8801	0,913375	1,159698
200	LPS	52	1,0698	0,1212	218,739	65,2539	0,610748	1,637336	218,739	53,70321	0,953093	9,457411	18,51119	18,54206	1,1838	2,4739	0,946105	1,215084
201	LPS	53	1,1416	0,1224	329,0202	102,9624	0,34682	2,883344	329,0202	67,50598	0,907293	11,88562	22,79248	23,49259	1,0851	1,8246	0,88926	1,525234
202	LPS	54	1,0774	0,1323	203,7121	63,5133	0,598165	1,67178	203,7121	52,36216	0,933665	8,602535	17,99885	18,1409	1,0665	2,3822	0,942593	1,212962
203	LPS	55	1,1204	0,1078	371,1458	100,63	0,416374	2,401689	371,1458	69,85959	0,955657	11,26069	24,09066	24,09372	1,0766	1,2135	0,904032	1,44046
204	LPS	56	1,076	0,114	212,0743	61,91543	0,658807	1,517895	212,0743	53,37596	0,935419	8,591489	19,03119	19,20551	1,168	2,0721	0,947673	1,159987
205	LPS	57	1,0333	0,1231	173,4696	51,84255	0,801813	1,247173	173,4696	47,67044	0,959256	7,962004	16,91195	16,91495	1,1728	2,8079	0,98583	1,08752
206	LPS	58	1,0469	0,1079	183,1523	54,13493	0,770663	1,297585	183,1523	48,67037	0,971611	8,615993	16,18241	16,22351	1,0563	3,8977	0,981291	1,112277
207	LPS	59	1,0665	0,1097	216,7899	63,16188	0,643756	1,553383	216,7899	55,61211	0,880866	10,522	20,75681	20,75755	1,3683	2,9172	0,94272	1,135758
208	LPS	60	1,0477	0,0999	283,9394	67,5461	0,764213	1,308535	283,9394	60,61782	0,971034	10,29108	20,25394	20,41691	1,0451	2,5698	0,977192	1,114294
209	LPS	61	1,1317	0,1321	325,6564	100,053	0,34325	2,91333	325,6564	67,20945	0,905959	11,29516	24,06454	24,13277	1,3101	2,4611	0,839656	1,488675
210	LPS	62	1,0283	0,0969	334,4588	74,67394	0,734953	1,360632	334,4588	66,82525	0,941176	11,33394	22,71995	22,80195	1,1708	3,0105	0,975092	1,117451
211	LPS	63	1,09	0,11	339,5201	85,89379	0,531498	1,881473	339,5201	67,47043	0,937234	11,09782	22,79248	23,02284	1,0736	1,3774	0,919074	1,273058

212	LPS	64	1,0391	0,0869	420,502	86,12979	0,680947	1,468543	420,502	76,45089	0,904092	13,30085	28,83592	28,8733	1,4871	3,0267	0,955966	1,126603
213	LPS	65	1,076	0,1102	334,5845	86,8211	0,467797	2,137678	334,5845	68,19521	0,904081	11,96525	23,60887	23,79296	1,2393	1,9003	0,838673	1,273126
214	LPS	66	1,0611	0,119	313,3645	83,30018	0,521387	1,917962	313,3645	66,95468	0,87841	11,56059	24,59367	24,76371	1,4418	2,0759	0,91874	1,244128
215	LPS	67	1,0881	0,2393	571,7456	115,8395	0,487938	2,049439	571,7456	88,37844	0,919856	14,21431	33,08342	33,08342	1,3735	1,7831	0,91131	1,310722
216	LPS	68	1,0251	0,0896	370,1084	77,75124	0,74883	1,335416	370,1084	70,74832	0,929194	12,5906	24,60199	25,19152	1,2501	3,8162	0,973329	1,098984
217	LPS	69	1,0472	0,1194	224,4605	60,12465	0,760161	1,31551	224,4605	54,98447	0,932974	9,242926	19,26106	19,36161	1,1403	1,8338	0,97423	1,093484
218	LPS	70	1,0735	0,0844	423,017	88,37606	0,663817	1,50644	423,017	73,642	0,980203	11,95559	25,0308	25,10807	1,1185	1,6301	0,975327	1,200077
219	LPS	71	1,0559	0,1317	178,311	57,38422	0,655147	1,526376	178,311	51,78988	0,835406	9,189238	20,38082	20,38082	1,6136	2,8627	0,9628	1,10802
220	LPS	72	1,0558	0,0846	529,5571	100,5158	0,617789	1,618677	529,5571	84,72736	0,92699	13,87349	30,84291	30,84291	1,2311	1,5756	0,937964	1,186344
221	LPS	73	1,0848	0,1133	217,5758	67,38156	0,569306	1,756523	217,5758	56,76048	0,848649	9,253865	22,16312	22,16312	1,82	1,7346	0,945384	1,187121
222	LPS	74	1,094	0,1053	388,0904	105,5264	0,398319	2,510549	388,0904	73,12952	0,91192	13,24762	26,88085	26,88248	1,2579	3,8481	0,909518	1,443007
223	LPS	75	1,0472	0,0921	373,4093	80,84628	0,688241	1,452979	373,4093	71,54738	0,916658	12,8364	26,82057	26,83429	1,517	2,4196	0,958663	1,129968
224	LPS	76	1,0307	0,0895	433,5798	87,92801	0,668095	1,496792	433,5798	78,71314	0,879395	14,23654	31,49548	31,49548	1,587	2,8838	0,948013	1,117069
225	LPS	77	1,0326	0,1071	263,7569	68,51667	0,68827	1,452918	263,7569	63,40374	0,824486	10,75025	24,30113	24,30129	1,7824	2,1421	0,974851	1,080641
226	LPS	78	1,0259	0,1214	311,6355	84,86117	0,457783	2,184441	311,6355	75,80431	0,681504	13,35949	31,80883	31,80883	2,5969	2,5118	0,841824	1,119477
227	LPS	79	1,0748	0,1081	380,6712	91,88652	0,524977	1,904844	380,6712	73,6492	0,881908	12,95759	26,23754	26,37511	1,3665	2,4961	0,926584	1,247624
228	LPS	80	1,0444	0,1305	185,5415	56,18387	0,712224	1,404053	185,5415	50,15209	0,926984	9,437908	17,92184	17,92535	1,3553	4,7564	0,964249	1,12027
229	LPS	81	1,0459	0,1121	296,0741	73,29557	0,671524	1,489149	296,0741	64,74	0,887697	10,6264	25,53684	25,53684	1,6124	2,4787	0,969633	1,132153
230	LPS	82	1,046	0,0877	446,689	89,03475	0,669729	1,493141	446,689	76,617	0,956235	13,00598	25,10603	26,09846	1,0138	2,3667	0,945809	1,162076
231	LPS	83	1,1019	0,08	622,485	123,7553	0,482843	2,071065	622,485	95,23225	0,862522	14,71832	36,39074	36,39074	1,7084	1,859	0,945356	1,299511
232	LPS	84	1,0319	0,0949	323,4558	72,07004	0,758825	1,317826	323,4558	64,8255	0,967237	10,3258	21,35034	21,62929	1,0583	1,6781	0,969676	1,111754
233	LPS	85	1,0327	0,105	366,0845	83,4828	0,620515	1,611564	366,0845	73,81078	0,844406	13,56168	28,97404	28,97404	1,7688	3,4796	0,94006	1,131038
234	LPS	86	1,0495	0,092	474,668	95,9266	0,607047	1,647318	474,668	78,57335	0,96616	13,7753	26,56617	26,90881	1,1135	2,3449	0,936486	1,220854
235	LPS	87	1,0522	0,0937	523,144	102,6539	0,578188	1,729541	523,144	84,6517	0,917401	14,71387	30,73878	30,74082	1,3906	2,2234	0,926807	1,212662
236	LPS	88	1,0453	0,0881	428,5813	86,85674	0,684101	1,461772	428,5813	76,76287	0,913988	13,1492	28,30945	28,30956	1,2859	1,9554	0,958263	1,131494
237	LPS	89	1,0441	0,0857	472,6875	91,03032	0,691174	1,446814	472,6875	81,41011	0,896245	13,22924	28,37433	29,062	1,0949	1,4973	0,964219	1,11817
238	LPS	90	1,0647	0,0873	407,4556	86,32695	0,657325	1,521317	407,4556	73,71892	0,942176	13,64502	25,19977	25,2975	1,1774	3,1033	0,956716	1,171028
239	LPS	91	1,0653	0,0941	380,9227	83,56879	0,653179	1,530974	380,9227	71,01902	0,949067	11,41004	24,6989	24,72706	1,1094	1,3614	0,952959	1,17671
240	LPS	92	1,0573	0,0924	313,6789	72,77926	0,707264	1,413898	313,6789	65,41016	0,921307	11,4409	22,49539	22,59913	1,1659	2,0268	0,950391	1,11266
241	LPS	93	1,0568	0,1054	400,508	89,56152	0,572485	1,746771	400,508	72,91301	0,946696	12,15846	24,43273	24,56743	1,1193	1,5776	0,912402	1,228334
242	LPS	94	1,0809	0,1055	402,8344	93,05408	0,535154	1,868622	402,8344	74,12684	0,921266	13,37399	26,10782	26,39608	1,2685	2,5394	0,915405	1,255336
243	LPS	95	1,1206	0,1805	949,2103	160,6612	0,413094	2,420759	949,2103	112,7002	0,939123	18,47275	40,50051	40,50051	1,1607	1,6111	0,893919	1,425562
244	LPS	96	1,0479	0,1019	271,8047	65,37553	0,782619	1,277761	271,8047	59,35571	0,969486	9,609184	20,50768	20,51255	1,1536	1,5915	0,979297	1,101419
245	LPS	97	1,0927	0,1025	458,6666	104,5989	0,464053	2,154927	458,6666	79,69745	0,90744	12,98991	28,42415	28,55073	1,127	1,3942	0,880877	1,31245
246	LPS	98	1,1309	0,218	733,5836	150,1624	0,358069	2,792755	733,5836	99,39482	0,933107	16,70436	35,73695	35,73695	1,1973	1,9126	0,875852	1,510767
247	LPS	99	1,0595	0,1179	200,3169	59,05337	0,699858	1,428862	200,3169	52,28785	0,920715	8,601755	18,2314	18,63876	1,2163	2,3174	0,969554	1,12939
248	LPS	100	1,0719	0,0848	347,5366	80,2156	0,657664	1,520533	347,5366	68,72541	0,924646	13,75176	24,69573	24,74158	1,2353	4,3437	0,968973	1,16719
249	LPS	101	1,0859	0,22	586,1124	111,577	0,550937	1,815089	586,1124	87,83933	0,95458	14,25135	29,71379	29,87592	1,1043	1,2079	0,931238	1,270239
250	LPS	102	1,1024	0,1054	467,0603	114,9583	0,379104	2,6378	467,0603	80,72106	0,900759	13,84032	28,24441	28,70938	1,323	1,8953	0,853604	1,424143
251	LPS	103	1,0425	0,1028	299,9409	71,26436	0,708639	1,411156	299,9409	63,50115	0,934721	10,59739	22,27066	22,37566	1,2516	3,3804	0,954827	1,122253
252	LPS	104	1,0552	0,0993	420,9421	93,60603	0,572685	1,746161	420,9421	76,81468	0,896486	12,1669	28,65546	28,66	1,5361	1,8745	0,948618	1,218596
253	LPS	105	1,0379	0,0844	463,3821	87,78103	0,717655	1,393426	463,3821	78,40993	0,947124	12,83559	27,1375	27,3866	1,1678	2,0031	0,949661	1,119514
254	LPS	106	1,0718	0,2177	613,997	111,4835	0,57729	1,732233	613,997	93,66172	0,879532	15,4916	36,03089	36,03089	1,5063	2,0847	0,929906	1,190278
255	LPS	107	1,0479	0,1144	262,7823	71,05656	0,624375	1,6016	262,7823	62,02087	0,858479	10,07177	24,45587	24,45587	1,6221	2,0833	0,95466	1,145688
256	LPS	108	1,061	0,0919	410,6622	88,03191	0,638992	1,564965	410,6622	74,29711	0,934868	12,62972	26,74486	26,7919	1,3263	2,4248	0,95958	1,184863
257	LPS	109	1,0569	0,1091	224,4605	61,59645	0,716147	1,396362	224,4605	54,1552	0,961766	8,976578	18,05553	18,35154	1,1129	1,8099	0,963305	1,137406
258	LPS	110	1,0639	0,1261	179,0026	55,65408	0,696003	1,436775	179,0026	48,84094	0,942976	8,754113	17,7305	17,73574	1,3411	2,7959	0,958377	1,139496
259	LPS	111	1,0658	0,0979	309,5921	73,77926	0,679223	1,472271	309,5921	63,9061	0,952609	10,31842	22,44502	22,51947	1,2219	1,3277	0,950345	1,154495
260	LPS	112	1,1382	0,1058	472,3102	117,5695	0,375684	2,661813	472,3102	79,01598	0,95062	12,54915	26,83404	27,33784	1,103	1,4088	0,874933	1,487921
261	LPS	113	1,0466	0,0945	434,5858	88,44734	0,66406	1,505887	434,5858	75,2403	0,964682	12,42479	24,9805	25,45284	1,0484	1,6315	0,951244	1,175531
262	LPS	114	1,0787	0,113	308,4603	80,61028	0,558649	1,790034	308,4603	65,5225	0,902876	10,76128	23,87041	24,06546	1,4233	1,6633	0,936506	1,230269
263	LPS	115	1,0684	0,1123	345,3989	85,99025	0,545961	1,831634	345,3989	67,89112	0,941683	11,08163	23,71913	23,78996	1,2752	1,9739	0,930099	1,266591
264	LPS	116	1,0945	0,1061	324,9019	88,08227	0,497778	2,008929	324,9019	66,15589	0,932878	10,43301	24,84358	24,84358	1,4209	1,4913	0,945912	1,331435
265	LPS	117	1,0793	0,0912	334,4588	81,18298	0,612114	1,633683	334,4588	67,69652	0,917106	10,82424	25,07972	25,07972	1,3946	1,6563	0,959865	1,199219
266	LPS	118	1,0622	0,1188	129,8979	44,67482	0,810945	1,233129	129,8979	41,32491	0,955846	7,064397	13,74543	14,05108	1,0106	2,3814	0,99153	1,081063
267	LPS	119	1,0792	0,0764	531,4748	101,4683	0,622283	1,606985	531,4748	83,45972	0,958823	13,44112	29,12824	29,20041	1,1466	1,4368	0,959304	1,215775
268	LPS	120	1,1339	0,2185	658,9206	135,9379	0,388804	2,571989	658,9206	95,18881	0,913841	16,79103	34,54491	34,54491	1,2355	1,6859	0,8677	1,428087
269	LPS	121	1,0732	0,0959	329,2088	80,00798	0,616648	1,621671	329,2088	65,50926	0,963997	11,12768	21,92353	21,94404	1,0965	2,281	0,954163	1,221323
270	LPS	122	1,0449	0,0955	459,924	99,09291	0,541637	1,846256	459,924	82,03647	0,85878	13,67264	33,07296	33,07296	1,6512	2,1662	0,920232	1,207913
271	LPS	123	1,0689	0,0903	472,0902	105,0784	0,504122	1,983647	472,0902	85,32262	0,814903	14,01424	30,0839	31,72651	1,3743	2,7507	0,93827	1,231542
272	LPS	124	1,088	0,1113	394,4721	105,4402	0,394742	2,5333	394,4721	79,90746	0,776338	13,95856	30,63684	30,82121	1,7339	2,5419	0,88532	1,319529
273	LPS	125	1,0502	0,1259	247,5039	76,9055	0,48833	2,047796	247,5039	66,16092	0,71054	11,12009	27,02083	27,02083	2,1088	2,8632	0,928617	1,162401
274	LPS	126	1,1045	0,0802	595,5749	123,8339	0,449102	2,226668	595,5749	92,49849	0,874735	15,19211	32,59954	34,07846	1,2459	2,0945	0,92019	1,338766
275	LPS	127	1,0446	0,0907	267,3407	64,1039	0,807439	1,238483	267,3407	58,56461	0,979499	9,801241	19,12018	19,52057	1,0209	2,6799	0,987653	1,094584
276	LPS	128	1,0839	0,1117	275,2943	75,4305	0,577046	1,732963	275,2943	60,53227	0,944132	9,474663	21,69796	21,69801	1,2429	1,356	0,949069	1,24612
277	LPS	129	1,0803	0,1078	327,6998	81,6133	0,572106	1,747927	327,6998	65,51943	0,95928	10,81681	22,49817	22,57362	1,1137	1,6756	0,925365	1,245635
278	LPS	130	1,0656	0,0976	359,9542	83,58652	0,611739	1,634685	359,9542	69,005	0,949939	11,14748	24,59367	24,62465	1,2437	1,7905	0,944891	1,211311
279	LPS	131	1,0304	0,0889	455,6486	90,16986	0,663663	1,506789	455,6486	79,76266	0,899995	13,9381	29,72014	29,72014	1,3045	2,5784	0,94239	1,130477
280	KYNA	1	1,0449	0,1044	194,4382	54,09929	0,832149	1,201708	194,4382	49,76411	0,986639	9,387004	16,1766	16,38436	1,0191	5,1272	0,996766	1,087115
281	KYNA	2	1,1124	0,1393	354,327	105,1257	0,321683	3,108654	354,327	73,25378	0,829762	13,14855	28,49374	28,52034	1,5852	3,035	0,798421	1,435089
282	KYNA	3	1,0642	0,108	231,3453	68,81773	0,582162	1,717735	231,3453	56,21465	0,919965	9,400124	20,93401	20,93401	1,2627	2,3393	0,948363	1,224196
283	KYNA	4	1,0558	0,1183	223,2345	64,14699	0,653705	1,529741	223,2345	54,60839	0,940702	8,935301	18,5502	18,8041	1,0615	1,9876	0,958879	1,174673
284	KYNA	5	1,1399	0,1147	256,3377	83,44716	0,417774	2,393636	256,3377	60,35606	0,88426	9,651631	21,87401	22,14365	1,2826	2,2146	0,903115	1,382581
285	KYNA	6	1,0594	0,1122	194,2495	56,50284	0,746896	1,338875	194,2495	50,13878	0,971006	8,035461	16,57968	16,6906	1,0827	1,3823	0,976857	1,126929
286	KYNA	7	1,0518	0,0951	258,1925	64,24344	0,767467	1,302987	258,1925	58,53663	0,946886	10,5758	20,25083	20,38904	1,2146	2,9332	0,976257	1,097491
287	KYNA	8	1,0592	0,1297	201,26	61,68245	0,614993	1,626036	201,26	51,93762	0,937568	9,570213	17,80569	18,28874	1,1705	2,93	0,92518	1,187626
288	KYNA	9	1,0503	0,1131	218,8962	61,39911	0,698227	1,432198	218,8962	54,8417	0,914589	8,784326	20,99848	20,99848	1,3195	2,6619	0,956915	1,11957
289	KYNA	10	1,0635	0,1132	265,9574	70,11454	0,644641	1,551252	265,9574	59,68922	0,938058	10,497	21,5066	21,55004	1,2475	2,8586	0,948227	1,17466
290	KYNA	11	1,0433	0,1196	318,143	84,29574	0,519818	1,923749	318,143	72,53539	0,759857	13,20434	30,7919	30,7919	2,2499	2,9678	0,923913	1,162133
291	KYNA	12	1,0635	0,118	301,8586	76,72004	0,599021	1,669391	301,8586	64,07619	0,923889	11,26996	23,59755	23,61718	1,2917	2,7202	0,929494	1,197325
292	KYNA	13	1,1018	0,1477	378,4707	107,4181	0,313851	3,186224	378,4707	83,76411	0,677838	14,93585	37,12963	37,12963	2,1775	3,3457	0,761442	1,282388
293	KYNA	14	1,0299	0,1365	186,4217	53,50124	0,803104	1,245168	186,4217	49,48441	0,956685	8,938901	17,58541	17,58541	1,1473	3,7584	0,981282	1,081174
294	KYNA	15	1,0547	0,1355	281,393	78,13103	0,497722	2,009153	281,393	66,55152	0,798375	11,47885	26,26628	26,31101	1,708	2,2195	0,859234	1,173993
295	KYNA	16	1,0381	0,1226	297,0487	79,09096	0,539836	1,852413	297,0487	72,27213	0,714653	12,27154	31,18149	31,18149	2,4337	3,3562	0,904646	1,094349
296	KYNA	17	1,0021	0,1551	206,6672	55,67199	0,76158	1,313059	206,6672	53,31583	0,913626	10,8986	18,2495	18,59922	1,0466	3,6548	0,908883	1,044192
297	KYNA	18	1,0845	0,2345	520,3775	105,4833	0,544639	1,836078	520,3775	83,30316	0,942335	14,09183	27,90512	29,01023	1,0859	2,0704	0,92672	1,266259
298	KYNA	19	1,0273	0,0801	449,7384	83,46791	0,797182	1,253946	449,7384	76,96262	0,954135	13,27161	27,40379	27,48654	1,24	2,9919	0,983084	1,084525
299	KYNA	20	1,0671	0,1242	227,8557	65,2539	0,619098	1,615253	227,8557	54,42154	0,96678	8,892128	18,1597	18,28477	1,0236	1,416	0,920668	1,199045
300	KYNA	21	1,0349	0,0863	397,3329	79,94716	0,758879	1,317732	397,3329	72,0347	0,962234	12,25764	24,6173	24,89039	1,1464	2,2625	0,971438	1,109842
301	KYNA	22	1,1321	0,1289	366,7761	105,7488	0,353053	2,832435	366,7761	69,93628	0,942335	12,36129	23,47199	23,60798	1,1337	1,8482	0,856604	1,512073
302	KYNA	23	1,1318	0,1382	336,2821	102,636	0,330128	3,029126	336,2821	68,5575	0,89909	11,77716	24,16817	24,23096	1,217	1,4148	0,822941	1,497079
303	KYNA	24	1,0242	0,1121	208,239	56,9539	0,794666	1,25839	208,239	52,67968	0,942943	8,85906	19,00475	19,01289	1,2417	2,286	0,985054	1,081136
304	KYNA	25	1,0726	0,1115	279,2239	72,69025	0,635503	1,573556	279,2239	60,27654	0,965752	9,754521	20,98874	20,98874	1,1409	2,6415	0,956992	1,205946
305	KYNA	26	1,0652	0,117	172,7152	52,25798	0,774071	1,29187	172,7152	47,75803	0,951584	8,589397	16,57303	16,57303	1,1439	4,4829	0,973972	1,094224
306	KYNA	27	1,1158	0,1215	506,5766	127,7746	0,329321	3,036547	506,5766	83,20589	0,919489	13,82592	31,0547	31,0547	1,3015	2,1042	0,844607	1,535644
307	KYNA	28	1,0425	0,1313	143,9817	46,46259	0,833003	1,200475	143,9817	43,52392	0,955126	7,620355	15,1966	15,1966	1,0757	2,3269	0,993886	1,067519
308	KYNA	29	1,1122	0,101	532,4808	124,6691	0,379102	2,63781	532,4808	89,19803	0,841013	14,56965	35,44548	35,44548	1,5674	1,9221	0,880564	1,397667
309	KYNA	30	1,0212	0,1113	218,6132	56,3055	0,863416	1,15819	218,6132	53,04246	0,976424	9,576684	18,21415	18,21445	1,1316	4,7348	0,996405	1,061517
310	KYNA	31	1,0532	0,1122	214,7151	58,75957	0,761909	1,312493	214,7151	53,13956	0,955512	9,790124	18,63053	18,65025	1,1834	3,1534	0,974963	1,10576
311	KYNA	32	1,0377	0,09	385,8584	81,38333	0,68718	1,455223	385,8584	73,92809	0,887194	12,39507	27,11839	27,14062	1,4761	2,0962	0,938651	1,100845
312	KYNA	33	1,0957	0,1977	1686,786	230,8943	0,289832	3,450278	1686,786	165,9343	0,769834	29,15156	62,86755	63,1077	1,4754	1,6592	0,728959	1,39148
313	KYNA	34	1,0539	0,1143	214,3378	58,92429	0,754014	1,326235	214,3378	53,13956	0,970326	9,335142	18,39363	18,39363	1,1708	2,8544	0,971986	1,118405
314	KYNA	35	1,0394	0,1317	228,3902	66,62181	0,582454	1,716873	228,3902	58,20677	0,84711	10,0152	22,36293	22,36293	1,4029	2,1689	0,900757	1,144571
315	KYNA	36	1,0398	0,1115	167,6224	50,23422	0,822355	1,21602	167,6224	46,84034	0,960067	7,473103	15,82789	16,16404	1,0985	1,7743	0,985184	1,072456
316	KYNA	37	1,0306	0,1169	177,1792	50,76082	0,863642	1,157887	177,1792	47,99096	0,966726	8,213582	15,86755	16,205	1,0607	4,1253	0,999468	1,057716
317	KYNA	38	1,0247	0,1258	172,1493	50,71046	0,835402	1,197029	172,1493	47,47349	0,95987	7,797961	16,84677	16,84677	1,1714	3,288	0,993061	1,068185

318	KYNA	39	1,0405	0,0966	278,9409	68,95727	0,722455	1,38417	278,9409	63,46681	0,870219	10,52395	24,55594	24,58488	1,7046	2,5775	0,980052	1,086509
319	KYNA	40	1,0513	0,1177	205,9127	58,65567	0,730163	1,369556	205,9127	52,99188	0,921455	8,811028	19,8534	19,86832	1,321	3,0528	0,97084	1,10688
320	KYNA	41	1,0295	0,1288	144,3904	46,42996	0,838756	1,192242	144,3904	43,45784	0,960753	7,695408	15,63906	15,63906	1,2456	2,8658	0,996516	1,068391
321	KYNA	42	1,0383	0,1107	183,4352	52,19716	0,844025	1,1848	183,4352	48,49718	0,980075	9,183954	16,08303	16,11216	1,064	6,0964	0,997601	1,076293
322	KYNA	43	1,0493	0,1064	197,1417	55,16312	0,807373	1,238585	197,1417	50,50968	0,971044	8,812642	17,68878	17,695	1,1538	3,2946	0,991708	1,09213
323	KYNA	44	1,0568	0,1132	198,1477	55,79362	0,786944	1,270738	198,1477	50,78365	0,965497	8,678209	17,52452	17,53199	1,1711	2,7963	0,983817	1,098653
324	KYNA	45	1,0527	0,1088	209,1821	58,04291	0,764189	1,308577	209,1821	52,16963	0,965825	8,466099	18,00496	18,01069	1,1859	1,8633	0,979411	1,11258
325	KYNA	46	1,0166	0,1133	193,2121	54,96578	0,798667	1,252087	193,2121	51,38711	0,919466	9,906312	18,5502	18,56372	1,2979	3,669	0,993817	1,069641
326	KYNA	47	1,0332	0,1272	197,5504	57,6883	0,732184	1,365777	197,5504	51,98624	0,918567	8,316028	19,54059	19,54255	1,4422	1,7075	0,98154	1,109684
327	KYNA	48	1,029	0,135	184,1897	56,03706	0,71659	1,395498	184,1897	50,7253	0,89955	8,790195	18,10683	18,52009	1,26	2,8674	0,97218	1,104716
328	KYNA	49	1,0741	0,1379	202,3289	64,58759	0,561575	1,780706	202,3289	53,99404	0,872119	8,991011	20,71131	20,71131	1,5999	1,5933	0,92138	1,196198
329	KYNA	50	1,0432	0,123	595,6378	133,8848	0,307707	3,24985	595,6378	109,5285	0,623933	20,50702	45,78624	45,86601	3,0222	1,9371	0,736898	1,222374
330	KYNA	51	1,0566	0,1337	235,715	69,45567	0,557677	1,793152	235,715	57,63284	0,891778	9,872535	20,97002	20,97002	1,4132	2,748	0,908242	1,205141
331	KYNA	52	1,0853	0,1151	233,5773	67,57145	0,612141	1,633612	233,5773	55,98934	0,936331	8,932589	20,13016	20,17516	1,3423	1,7811	0,952221	1,206863
332	KYNA	53	1,09	0,1274	352,5036	92,23369	0,426299	2,345769	352,5036	73,06989	0,829652	12,90702	25,77028	26,46037	1,258	1,2904	0,818693	1,262267
333	KYNA	54	1,1354	0,2545	607,0809	139,7927	0,336809	2,969045	607,0809	90,06429	0,940483	14,68702	31,24846	31,26387	1,0709	1,3302	0,862773	1,552144
334	KYNA	55	1,0592	0,1118	141,6553	45,99663	0,837082	1,194626	141,6553	42,97456	0,963873	7,207819	14,55629	14,6691	1,1079	2,9985	0,994896	1,070322
335	KYNA	56	1,0749	0,1135	285,92	75,89947	0,576795	1,733718	285,92	63,79358	0,882877	10,40337	24,38637	24,38637	1,4126	1,9456	0,924794	1,189767
336	KYNA	57	1,0802	0,0898	550,6199	117,5296	0,457962	2,183587	550,6199	92,09707	0,815774	15,96427	36,33367	36,33367	2,0151	2,7467	0,914245	1,276149
337	KYNA	58	1,0514	0,1088	255,2688	65,98085	0,708162	1,412106	255,2688	58,02044	0,952895	9,35039	20,82333	20,82333	1,1993	1,7004	0,961084	1,1372
338	KYNA	59	1,0745	0,1296	308,3346	83,85514	0,464957	2,150739	308,3346	67,02578	0,862478	11,11078	24,83853	24,88355	1,2485	1,3438	0,843801	1,251088
339	KYNA	60	1,1259	0,0898	593,4058	128,147	0,393735	2,539782	593,4058	90,49342	0,910599	14,10415	32,9253	32,94612	1,2324	1,5807	0,86708	1,416092
340	KYNA	61	1,0389	0,1038	232,0997	59,16773	0,820607	1,21861	232,0997	54,85456	0,969301	8,733067	18,44739	18,4711	1,1124	1,3085	0,984965	1,078629
341	KYNA	62	1,072	0,1048	321,5382	75,96028	0,637629	1,568311	321,5382	66,09021	0,925055	10,74232	22,86684	23,28441	1,1322	1,7446	0,91054	1,149342
342	KYNA	63	1,0442	0,1142	173,8783	52,25798	0,779568	1,282761	173,8783	48,4908	0,929258	9,357004	16,37362	16,81982	1,0565	4,5025	0,974327	1,077689
343	KYNA	64	1,0183	0,1084	277,0233	69,20798	0,708405	1,411622	277,0233	64,84484	0,827894	10,49617	26,52947	26,52947	1,7097	2,7987	0,974694	1,067286
344	KYNA	65	1,0242	0,1472	117,3545	41,23245	0,870909	1,148226	117,3545	38,77066	0,981077	7,216613	12,81021	12,90147	1,0672	6,8207	1,004018	1,063496
345	KYNA	66	1,0103	0,1184	193,7465	53,67642	0,847232	1,180314	193,7465	50,24695	0,964326	9,385869	17,34057	17,34796	1,1834	8,4364	1,002596	1,068252
346	KYNA	67	1,0355	0,1277	156,1793	48,60798	0,825968	1,210701	156,1793	45,31537	0,955744	8,396986	15,8636	15,8636	1,1258	4,8372	0,994364	1,07266
347	KYNA	68	1,031	0,1393	230,8737	75,53759	0,461451	2,167079	230,8737	66,77176	0,650726	11,52846	30,12828	30,12828	2,7254	3,8418	0,907544	1,131281
348	KYNA	69	1,0505	0,0959	321,6639	73,81489	0,710976	1,406516	321,6639	64,88337	0,960163	10,35895	22,1936	22,43074	1,1668	1,5208	0,958366	1,137655
349	KYNA	70	1,0295	0,1016	349,9887	87,73369	0,52417	1,907779	349,9887	81,20339	0,666984	15,11596	35,37979	35,37979	2,6137	3,2272	0,917363	1,080419
350	KYNA	71	1,0385	0,1206	156,5879	48,29645	0,840045	1,190413	156,5879	44,99599	0,971897	7,076365	15,64509	15,6789	1,2169	1,383	0,995784	1,07335
351	KYNA	72	1,0477	0,105	228,2959	59,63351	0,791064	1,264121	228,2959	54,82952	0,954286	9,752199	19,28145	19,33379	1,2081	3,4241	0,980584	1,087617
352	KYNA	73	1,0899	0,2238	726,0072	141,9055	0,375291	2,664601	726,0072	109,7694	0,75716	19,01048	45,4361	45,4361	2,1472	2,1722	0,828354	1,29276
353	KYNA	74	1,0633	0,1253	275,7029	75,3445	0,553173	1,807754	275,7029	61,41115	0,918663	9,801082	23,4217	23,4217	1,3722	1,6552	0,906385	1,226886
354	KYNA	75	1,0548	0,1902	671,3382	106,8839	0,70955	1,409343	671,3382	94,25422	0,949619	15,29789	33,98062	33,98062	1,308	1,9045	0,960852	1,133996
355	KYNA	76	1,0652	0,2091	553,9837	104,8853	0,575898	1,73642	553,9837	89,4986	0,869109	15,73197	33,9334	33,99553	1,6944	1,6092	0,910056	1,171921
356	KYNA	77	1,0494	0,2256	615,6318	120,356	0,502812	1,988814	615,6318	106,8148	0,678059	19,34814	46,91115	46,91115	2,845	2,7504	0,94148	1,126773
357	KYNA	78	1,0835	0,0975	360,1429	88,97394	0,519439	1,925153	360,1429	70,82028	0,902337	12,15392	24,54314	25,19566	1,1683	1,8494	0,908607	1,256334
358	KYNA	79	1,0142	0,1331	160,3918	47,98493	0,869346	1,15029	160,3918	45,57137	0,970527	7,464504	15,30995	15,31122	1,06	1,4099	0,99314	1,052962
359	KYNA	80	1,0436	0,1043	200,6627	55,1305	0,813268	1,229607	200,6627	51,71078	0,943005	9,633777	17,99885	17,99885	1,0814	2,9058	0,98026	1,066132
360	KYNA	81	1,045	0,1135	212,263	56,69273	0,81995	1,219586	212,263	52,38645	0,971954	9,251649	18,01369	18,01369	1,123	3,2244	0,988004	1,082202
361	KYNA	82	1,0389	0,1	400,4766	86,62376	0,622451	1,606553	400,4766	74,65867	0,902781	13,37117	27,52113	27,533	1,4057	3,1681	0,928095	1,160264
362	KYNA	83	1,036	0,1242	350,4602	94,23528	0,391744	2,552684	350,4602	84,63525	0,614817	16,69108	36,39938	36,39938	2,7851	1,4644	0,789917	1,113428
363	KYNA	84	1,0409	0,104	318,0486	74,10869	0,708372	1,411689	318,0486	65,92089	0,919723	10,99449	25,05715	25,05715	1,4717	2,3388	0,973411	1,124206
364	KYNA	85	1,0284	0,1072	207,9561	55,07713	0,86329	1,158359	207,9561	51,49083	0,985647	9,550089	17,31426	17,31832	1,1077	5,188	1,002116	1,069649
365	KYNA	86	1,0629	0,127	317,64	82,31507	0,498376	2,006517	317,64	69,53073	0,825641	13,33466	26,96959	26,96989	1,5088	2,1473	0,846002	1,183866
366	KYNA	87	1,0431	0,1186	159,2915	48,96259	0,828054	1,207651	159,2915	45,69559	0,958636	7,814113	16,21348	16,25199	1,264	3,4372	0,991711	1,071495
367	KYNA	88	1,0616	0,1416	177,808	58,5445	0,587481	1,702183	177,808	50,47346	0,877071	9,233298	18,50863	18,68842	1,3023	2,4418	0,901167	1,159907
368	KYNA	89	1,0455	0,1427	142,2527	47,92394	0,750811	1,331893	142,2527	43,657	0,937912	7,911152	16,08303	16,08303	1,2395	2,9682	0,964641	1,097738
369	KYNA	90	1,0387	0,1309	190,0684	55,6289	0,753824	1,326569	190,0684	50,04278	0,953754	9,254504	17,56035	17,56206	1,1571	3,5244	0,976679	1,111627
370	KYNA	91	1,0715	0,1138	344,2671	82,76613	0,539729	1,852783	344,2671	69,39594	0,898332	12,09566	25,57927	25,57927	1,2563	1,8979	0,854625	1,192665
371	KYNA	92	1,0344	0,1323	273,4395	72,69025	0,584429	1,711043	273,4395	64,07418	0,83696	10,97881	23,33431	23,82674	1,5515	1,7335	0,898712	1,13447
372	KYNA	93	1,0342	0,1243	154,8903	48,43599	0,820057	1,219428	154,8903	45,03858	0,959543	7,306223	16,02429	16,02429	1,2115	2,429	0,988431	1,075433
373	KYNA	94	1,0979	0,1288	315,3451	91,22021	0,426852	2,342733	315,3451	70,29947	0,801847	10,90009	29,34706	29,34706	1,8465	2,1485	0,896321	1,297595
374	KYNA	95	1,045	0,1101	209,0878	58,39752	0,752851	1,328284	209,0878	52,96294	0,936686	8,380284	18,82441	18,99289	1,2899	1,6833	0,977146	1,102611
375	KYNA	96	1,0403	0,1387	164,8559	52,28316	0,725056	1,379203	164,8559	46,20688	0,970288	7,622039	15,83484	15,83484	1,1438	2,3471	0,956712	1,131502
376	KYNA	97	1,0337	0,1112	240,1476	62,70337	0,750669	1,332146	240,1476	55,99656	0,962421	10,43202	19,22103	19,22103	1,1535	4,1867	0,978008	1,119772
377	KYNA	98	1,0975	0,1221	348,1653	96,85106	0,419387	2,384434	348,1653	71,89512	0,84644	11,2936	28,66041	28,66041	1,5284	1,7216	0,899142	1,347116
378	KYNA	99	1,0564	0,1171	185,2271	54,1422	0,779755	1,282455	185,2271	48,78039	0,978191	7,698014	16,36209	16,48312	1,0732	1,2128	0,98201	1,109917
379	KYNA	100	1,0324	0,1238	197,9905	55,2922	0,797793	1,253458	197,9905	50,90941	0,95997	9,031277	17,46071	17,55885	1,1928	2,8011	0,980311	1,08609
380	KYNA	101	1,0477	0,1189	184,8813	54,18528	0,773537	1,292764	184,8813	48,7953	0,955768	8,188848	15,94759	16,28472	1,0555	2,6348	0,977555	1,110461
381	KYNA	102	1,0271	0,1389	155,3619	50,1555	0,763064	1,310506	155,3619	45,14404	0,957973	7,176684	14,61342	15,52443	1,0403	3,2691	0,983205	1,11101
382	KYNA	103	1,0466	0,1132	168,2825	50,1734	0,830941	1,203455	168,2825	46,73576	0,968166	7,523298	16,23188	16,23188	1,1569	1,5976	0,989165	1,073555
383	KYNA	104	1,0545	0,1176	165,9248	52,93156	0,719669	1,389528	165,9248	47,22059	0,9351	8,147376	17,25333	17,28858	1,3513	2,4873	0,967033	1,120942
384	KYNA	105	1,0132	0,1255	248,5728	67,56401	0,655546	1,525446	248,5728	63,02027	0,786507	12,68392	26,25071	26,25131	1,7576	4,489	0,958012	1,0721
385	KYNA	106	1,0491	0,0954	380,5141	86,39096	0,59024	1,694226	380,5141	72,40199	0,912177	13,59323	26,63355	26,6378	1,3759	3,1107	0,921266	1,193213
386	KYNA	107	1,0504	0,1085	262,8766	70,71986	0,625201	1,599486	262,8766	64,36796	0,7973	11,29113	26,1367	26,14055	1,6618	3,1859	0,946544	1,098681
387	LPS+KYNA	1	1,0922	0,1109	361,4318	88,75585	0,528665	1,891558	361,4318	68,73422	0,961368	11,19801	22,82557	23,11762	1,0274	1,6892	0,916935	1,291291
388	LPS+KYNA	2	1,0443	0,107	450,3986	97,86436	0,518528	1,928537	450,3986	81,98424	0,842065	15,24656	31,51243	31,51243	1,5889	2,0145	0,877434	1,193697
389	LPS+KYNA	3	1,0708	0,1276	173,061	54,93759	0,682731	1,464706	173,061	48,36895	0,929555	8,310638	18,2081	18,2081	1,2772	2,5697	0,947502	1,135803
390	LPS+KYNA	4	1,0742	0,0973	404,0604	94,22482	0,517622	1,931913	404,0604	75,72872	0,88539	12,5652	27,77979	27,83651	1,4164	2,1047	0,905081	1,244242
391	LPS+KYNA	5	1,0753	0,1122	403,3374	96,0484	0,493614	2,025875	403,3374	75,14613	0,897562	12,48578	28,40424	28,40559	1,5159	1,6314	0,898441	1,278155
392	LPS+KYNA	6	1,0814	0,2268	619,3728	120,5103	0,495433	2,018438	619,3728	95,53447	0,852789	15,04674	37,02661	37,08032	1,4412	1,8727	0,924424	1,261433
393	LPS+KYNA	7	1,1418	0,1669	243,8886	90,67571	0,306398	3,26373	243,8886	62,68626	0,779931	10,19619	24,92633	25,12816	1,9574	2,0933	0,82199	1,446501
394	LPS+KYNA	8	1,047	0,1153	279,7269	73,82535	0,617706	1,618893	279,7269	64,2833	0,850643	11,18654	25,57129	25,57129	1,763	4,7823	0,957743	1,148438
395	LPS+KYNA	9	1,1819	0,1053	409,4047	132,9899	0,260041	3,845549	409,4047	73,66021	0,948192	12,53718	26,56617	26,61239	1,3232	3,2134	0,893957	1,805451
396	LPS+KYNA	10	1,103	0,3065	571,1483	127,3266	0,346045	2,889802	571,1483	90,9941	0,866826	16,53933	34,71965	34,71965	1,3932	2,2748	0,781649	1,399284
397	LPS+KYNA	11	1,1143	0,1299	200,5998	69,27624	0,465413	2,14863	200,5998	53,49706	0,880806	10,28757	20,42704	20,42816	1,6296	2,6197	0,886068	1,294954
398	LPS+KYNA	12	1,0528	0,1253	127,8545	43,88688	0,826789	1,209498	127,8545	40,94094	0,958539	6,573014	13,68009	14,06658	1,0575	1,6585	0,991148	1,071956
399	LPS+KYNA	13	1,0617	0,1098	236,2494	64,06099	0,703786	1,420886	236,2494	57,28787	0,904596	9,315798	20,79312	20,81883	1,2441	2,3249	0,972854	1,11823
400	LPS+KYNA	14	1,0247	0,0847	351,5605	71,7406	0,853544	1,171585	351,5605	67,14794	0,979815	11,29874	22,57977	22,58229	1,1322	4,4715	0,994366	1,068396
401	LPS+KYNA	15	1,0662	0,1043	228,7045	62,85018	0,711363	1,405753	228,7045	55,17101	0,944197	9,114362	20,35922	20,35929	1,3626	2,1486	0,977732	1,139188
402	LPS+KYNA	16	1,0878	0,1248	295,0053	87,65195	0,427091	2,341423	295,0053	62,57688	0,946698	10,77977	21,26255	21,53982	1,0671	2,9274	0,885124	1,400708
403	LPS+KYNA	17	1,0499	0,1149	200,3169	60,28191	0,682276	1,465682	200,3169	54,96658	0,833162	9,032606	22,34394	22,34516	1,8405	3,1863	0,984934	1,096701
404	LPS+KYNA	18	1,0554	0,1196	161,5236	53,5977	0,692952	1,443101	161,5236	48,94729	0,847204	8,143652	19,88979	19,88979	1,8725	3,5477	0,980732	1,095008
405	LPS+KYNA	19	1,0875	0,1458	146,2766	53,87376	0,601752	1,661815	146,2766	44,1642	0,942418	7,39367	15,27397	15,44665	1,3025	1,4171	0,95014	1,219851
406	LPS+KYNA	20	1,0391	0,1053	194,6897	54,65426	0,80502	1,242205	194,6897	50,16266	0,97228	8,108138	17,27245	17,27255	1,1281	1,5467	0,982884	1,089541
407	LPS+KYNA	21	1,0754	0,1335	468,6007	125,7539	0,277638	3,601814	468,6007	98,98195	0,601036	18,89615	44,18346	44,18346	3,1525	2,0865	0,745606	1,270473
408	LPS+KYNA	22	1,0542	0,0824	433,1082	85,70691	0,715057	1,39849	433,1082	75,76326	0,948175	12,62426	25,95989	25,95989	1,0932	1,6436	0,965087	1,131246
409	LPS+KYNA	23	1,085	0,2262	590,6707	114,0949	0,519605	1,924537	590,6707	88,6453	0,94459	15,20759	31,35691	31,35691	1,2132	1,7507	0,911278	1,287094
410	LPS+KYNA	24	1,0381	0,1184	278,4065	73,95443	0,601394	1,662803	278,4065	64,11977	0,850952	11,25082	26,27046	26,27046	1,6454	3,2053	0,940154	1,15338
411	LPS+KYNA	25	1,0538	0,097	306,9828	71,9305	0,713899	1,400758	306,9828	62,88543	0,975492	10,41177	20,97002	21,08335	1,0846	1,5089	0,957501	1,143834
412	LPS+KYNA	26	1,0426	0,0967	363,6324	77,80904	0,733558	1,363218	363,6324	69,03287	0,958871	11,20679	23,16461	23,55365	1,1036	2,041	0,971903	1,12713
413	LPS+KYNA	27	1,072	0,1154	306,9514	79,58209	0,570121	1,754015	306,9514	65,46316	0,900088	10,42404	24,39152	24,40754	1,3563	1,9099	0,936092	1,215678
414	LPS+KYNA	28	1,0555	0,2125	535,3415	97,00089	0,679494	1,471683	535,3415	84,00112	0,953389	14,13317	28,31167	28,40355	1,0982	2,5543	0,950379	1,154757
415	LPS+KYNA	29	1,043	0,1242	223,486	62,57429	0,668111	1,496757	223,486	56,01294	0,895124	9,594894	20,16294	20,49032	1,2072	2,1224	0,931495	1,11714
416	LPS+KYNA	30	1,0491	0,1258	209,2764	60,17057	0,699207	1,430192	209,2764	52,10239	0,968756	10,00977	17,27245	17,32348	1,0983	3,1754	0,962596	1,154852
417	LPS+KYNA	31	1,0796	0,0904	454,7055	97,0766	0,569568	1,755716	454,7055	77,60005	0,94889	12,62532	25,96716	26,84039	1,1446	1,6545	0,939367	1,250986
418	LPS+KYNA	32	1,0802	0,1071	421,225	104,0041	0,446404	2,240122	421,225	82,72633	0,773457	13,955	34,81007	34,81007	2,1454	3,2393	0,912232	1,257206
419	LPS+KYNA	33	1,054	0,1093	242,7255	63,25833	0,733606	1,363129	242,7255	56,5928	0,952363	9,973564	19,4244	19,46041	1,0671	3,2247	0,96244	1,117781
420	LPS+KYNA	34	1,0956	0,0995	482,15	112,4434	0,413561	2,418022	482,15	81,66688	0,908447	14,46945	30,15488	30,15807	1,4032	2,7978	0,863011	1,376855
421	LPS+KYNA	35	1,0509	0,1385	223,2659	73,20213	0,46593	2,146244	223,2659	65,00564	0,663942	11,78594	27,12012	27,12176	2,7466	2,2499	0,88989	1,126089
422	LPS+KYNA	36	1,0484	0,0923	289,9754	68,37763	0,746004	1,340476	289,9754	62,17216	0,942711	10,39844	21,91493	21,93037	1,2431	2,1236	0,957177	1,099803
423	LPS+KYNA	37	1,0922	0,1229	279,8841	80,36986	0,49258	2,030128	279,8841	60,86876	0,94929	9,789149	21,45683	21,53089	1,2063	1,3707	0,904639	1,320379

424	LPS+KYNA	38	1,0728	0,1162	229,0818	64,29379	0,649768	1,539011	229,0818	55,3592	0,939335	9,355745	19,68404	19,8077	1,2617	2,1535	0,933031	1,161393
425	LPS+KYNA	39	1,1222	0,111	307,6115	95,04238	0,400732	2,495431	307,6115	65,19371	0,909496	11,62904	22,99502	23,17394	1,2596	2,646	0,936433	1,457846
426	LPS+KYNA	40	1,2027	0,0846	572,4373	142,2897	0,328096	3,047887	572,4373	86,40085	0,96361	13,74789	30,49851	30,49851	1,182	1,2963	0,923444	1,646855
427	LPS+KYNA	41	1,0793	0,1049	276,8347	74,62801	0,602858	1,658764	276,8347	61,08871	0,932198	10,1683	22,66177	22,66177	1,3683	2,8037	0,965137	1,221634
428	LPS+KYNA	42	1,1429	0,1211	484,0991	132,0699	0,289315	3,456442	484,0991	80,76225	0,932668	13,34305	28,15567	28,2244	1,1536	1,3964	0,829534	1,635292
429	LPS+KYNA	43	1,1324	0,1049	286,2029	86,29876	0,460375	2,172142	286,2029	60,59353	0,97956	9,780213	20,3167	20,34355	1,0937	1,6191	0,953317	1,424224
430	LPS+KYNA	44	1,0985	0,1051	268,3152	76,01809	0,561939	1,779552	268,3152	58,43734	0,987356	9,401241	19,23821	19,28598	1,0596	1,5857	0,963093	1,300848
431	LPS+KYNA	45	1,1021	0,118	259,9844	80,04043	0,467784	2,137739	259,9844	61,67599	0,858864	10,83193	20,86631	22,23222	1,1752	2,1925	0,917291	1,297757
432	LPS+KYNA	46	1,0799	0,1187	224,4291	72,37128	0,514327	1,944288	224,4291	57,6141	0,849633	9,488174	23,4452	23,4452	1,7982	3,3662	0,955176	1,256138
433	LPS+KYNA	47	1,0518	0,1072	232,477	62,08014	0,73691	1,357018	232,477	56,16282	0,926172	9,593564	20,94301	20,9439	1,3653	4,3026	0,972143	1,10536
434	LPS+KYNA	48	1,0381	0,101	244,5174	62,03706	0,788744	1,267838	244,5174	56,1564	0,974363	9,159965	19,29287	19,32461	1,1129	1,9388	0,987915	1,104719
435	LPS+KYNA	49	1,0795	0,1022	368,1907	88,1	0,560487	1,784162	368,1907	72,53385	0,879429	12,82842	24,96451	26,50122	1,1795	3,1459	0,940232	1,214605
436	LPS+KYNA	50	1,0455	0,11	248,6356	64,73457	0,719194	1,390445	248,6356	58,16973	0,923376	9,662943	21,48977	21,48977	1,3221	1,8748	0,964597	1,112857
437	LPS+KYNA	51	1,0256	0,113	190,6028	55,36348	0,763132	1,310389	190,6028	50,0544	0,955992	9,496649	17,39035	17,44777	1,1913	3,4012	0,976579	1,106066
438	LPS+KYNA	52	1,0227	0,1076	243,8258	61,27748	0,797583	1,253788	243,8258	56,5583	0,957848	9,199131	19,88663	19,93121	1,2573	2,088	0,977437	1,083439
439	LPS+KYNA	53	1,0517	0,0943	380,2311	82,47234	0,662066	1,510423	380,2311	70,94337	0,949366	11,86002	25,44929	25,44929	1,2211	2,235	0,942456	1,16251
440	LPS+KYNA	54	1,0929	0,0945	546,5646	117,3649	0,455262	2,196538	546,5646	87,01096	0,907012	14,23539	33,46934	33,46934	1,4264	1,8827	0,913033	1,348712
441	LPS+KYNA	55	1,0964	0,1087	387,1787	97,92535	0,454769	2,198919	387,1787	73,82986	0,8926	11,74493	28,42746	28,45963	1,5125	2,9688	0,896314	1,326365
442	LPS+KYNA	56	1,0574	0,0982	277,7778	69,63085	0,688501	1,452431	277,7778	59,87039	0,973829	11,88507	19,91034	19,91034	1,0279	4,4934	0,956315	1,163027
443	LPS+KYNA	57	1,1225	0,0982	363,318	92,73511	0,482247	2,073626	363,318	73,44273	0,846445	12,28816	29,60516	29,60516	1,7249	2,0229	0,908367	1,262686
444	LPS+KYNA	58	1,0562	0,1152	254,3257	67,61436	0,656471	1,523297	254,3257	59,23514	0,910838	10,13317	22,21979	22,21979	1,4154	2,6882	0,939061	1,141457
445	LPS+KYNA	59	1,0555	0,0967	373,7551	80,55975	0,690284	1,448678	373,7551	69,90883	0,96102	11,10718	23,4217	23,55115	1,1151	1,4744	0,953823	1,152354
446	LPS+KYNA	60	1,0344	0,1079	289,378	70,84894	0,698715	1,4312	289,378	62,83268	0,921094	10,28762	23,47199	23,47232	1,4001	2,8197	0,964476	1,127581
447	LPS+KYNA	61	1,0616	0,088	474,7309	99,60496	0,554996	1,801815	474,7309	83,35514	0,858603	15,84578	31,71975	31,72628	1,7714	4,2781	0,922985	1,194947
448	LPS+KYNA	62	1,0306	0,1304	271,8362	69,72429	0,646433	1,546952	271,8362	61,27057	0,90994	12,97594	21,4077	21,92023	1,2822	3,5738	0,919972	1,137974
449	LPS+KYNA	63	1,0977	0,0941	499,5662	111,6807	0,456889	2,188714	499,5662	84,21436	0,885177	13,51252	31,52739	31,60842	1,4579	2,1908	0,907747	1,326148
450	LPS+KYNA	64	1,0665	0,1019	326,5681	77,30018	0,634425	1,57623	326,5681	67,7183	0,894893	11,85273	24,86129	24,9578	1,2115	2,4517	0,923758	1,141496
451	LPS+KYNA	65	1,0372	0,1187	161,8379	48,93741	0,839133	1,191706	161,8379	45,73115	0,972446	7,687518	15,75223	15,75642	1,1598	3,1068	0,988151	1,070111
452	LPS+KYNA	66	1,1101	0,1208	264,7628	81,64894	0,442898	2,257858	264,7628	61,16732	0,889258	9,797571	21,23296	22,00495	1,2747	1,6822	0,887438	1,334846
453	LPS+KYNA	67	1,0463	0,1083	270,6416	70,48688	0,653115	1,531125	270,6416	64,44181	0,818971	11,79491	24,88594	25,04035	1,7063	1,7696	0,954118	1,093807
454	LPS+KYNA	68	1,0921	0,203	659,4864	127,7924	0,476573	2,098313	659,4864	99,66067	0,834387	15,93784	41,17142	41,17142	1,7717	2,0092	0,939127	1,282275
455	LPS+KYNA	69	1,0679	0,1019	459,2953	101,7977	0,507213	1,971559	459,2953	80,56567	0,889204	13,36142	29,55466	29,65887	1,5521	1,675	0,910678	1,263537
456	LPS+KYNA	70	1,0625	0,1217	169,3514	52,93156	0,736166	1,35839	169,3514	47,42163	0,946336	7,470567	17,06739	17,06739	1,1813	1,5818	0,969185	1,11619
457	LPS+KYNA	71	1,0441	0,1258	288,6864	77,29273	0,519025	1,926689	288,6864	66,8806	0,811027	12,4367	25,31739	25,33273	1,5729	1,4652	0,854732	1,155682
458	LPS+KYNA	72	1,0917	0,1343	166,2391	58,55195	0,565668	1,767821	166,2391	49,00894	0,869745	8,090106	17,15374	18,00191	1,2539	2,2587	0,928328	1,19472
459	LPS+KYNA	73	1,0776	0,1165	264,9829	72,99433	0,558671	1,789961	264,9829	61,70963	0,874423	9,815071	22,38401	22,68968	1,3201	1,8912	0,893938	1,182868
460	LPS+KYNA	74	1,0263	0,1069	250,5533	62,0906	0,801116	1,248259	250,5533	57,35807	0,957019	9,377819	19,67766	19,75548	1,1151	2,2563	0,980928	1,082509
461	LPS+KYNA	75	1,0954	0,1226	289,0637	85,30621	0,440045	2,272497	289,0637	65,95202	0,835116	11,32379	25,57927	25,59355	1,881	1,8842	0,881566	1,293459
462	LPS+KYNA	76	1,1073	0,1082	381,2685	103,406	0,408357	2,448839	381,2685	77,32828	0,801242	13,42083	30,80262	30,82264	1,9451	2,5977	0,911362	1,337234
463	LPS+KYNA	77	1,0296	0,1284	175,2616	52,69858	0,785222	1,273525	175,2616	49,04101	0,915751	7,901135	18,49165	18,49165	1,421	2,5355	0,990135	1,074582
464	LPS+KYNA	78	1,1492	0,2012	746,5985	154,5216	0,348045	2,873188	746,5985	99,65186	0,944769	16,34113	34,28777	34,77885	1,2185	1,8313	0,885764	1,550615
465	LPS+KYNA	79	1,0799	0,1223	370,8314	95,45035	0,44952	2,224594	370,8314	72,68837	0,881975	11,91158	28,19651	28,19651	1,4576	2,4879	0,878857	1,313145
466	LPS+KYNA	80	1,1292	0,1179	357,4393	100,8986	0,388393	2,574709	357,4393	73,29142	0,836191	11,68262	29,1773	29,1773	1,6828	2,7429	0,880299	1,376677
467	LPS+KYNA	81	1,0293	0,1073	308,0831	72,6828	0,683119	1,463873	308,0831	66,89126	0,865244	13,26323	25,66209	25,66209	1,4751	2,4498	0,932143	1,086581
468	LPS+KYNA	82	1,0971	0,1116	485,4824	115,4879	0,373709	2,675875	485,4824	82,42926	0,897884	14,16188	29,03745	29,03745	1,2179	1,9085	0,817004	1,401055
469	LPS+KYNA	83	1,1506	0,2415	777,8784	171,5828	0,279172	3,582024	777,8784	103,2247	0,91739	16,97885	34,89126	35,77973	1,1042	1,379	0,84081	1,662227
470	LPS+KYNA	84	1,1166	0,1344	461,1187	121,6349	0,293449	3,407749	461,1187	81,93154	0,863217	13,69202	28,65163	30,20514	1,2159	1,6831	0,74925	1,484592
471	LPS+KYNA	85	1,0675	0,0893	316,7912	73,03741	0,728045	1,373541	316,7912	63,99965	0,971913	10,81949	21,53507	21,74082	1,1473	2,5295	0,975588	1,141216
472	LPS+KYNA	86	1,0893	0,095	490,0093	106,0353	0,501178	1,9953	490,0093	79,75486	0,968054	13,13498	27,98106	27,98106	1,117	1,5105	0,915122	1,329515
473	LPS+KYNA	87	1,0682	0,1354	221,6626	67,61436	0,567035	1,763561	221,6626	57,81824	0,833244	9,889149	22,77869	22,85223	1,8074	3,0255	0,930648	1,16943
474	LPS+KYNA	88	1,0672	0,2911	408,2415	98,22642	0,448669	2,228815	408,2415	85,45268	0,702547	15,69172	35,98417	36,02846	2,6444	2,4865	0,843832	1,149483
475	LPS+KYNA	89	1,0881	0,0959	475,1396	104,1048	0,510825	1,957618	475,1396	79,57823	0,942849	13,69144	26,64594	27,60904	1,0919	2,5044	0,92722	1,308207
476	LPS+KYNA	90	1,0457	0,1062	242,4111	64,13954	0,717044	1,394615	242,4111	56,79387	0,944407	9,096613	20,25472	20,28521	1,1447	1,7829	0,968357	1,129339
477	LPS+KYNA	91	1,0442	0,067	585,6722	100,694	0,708451	1,41153	585,6722	90,44762	0,899642	16,21966	33,7653	33,81399	1,5753	3,6024	0,976006	1,113285
478	LPS+KYNA	92	1,0424	0,089	343,104	75,12199	0,751203	1,331198	343,104	67,31566	0,951487	10,94826	23,83285	23,91846	1,255	1,5916	0,983233	1,115966
479	LPS+KYNA	93	1,0344	0,0826	605,6033	106,7193	0,624815	1,600475	605,6033	95,64757	0,831859	17,24934	37,7164	37,7164	1,7574	2,6302	0,93506	1,115756
480	LPS+KYNA	94	1,0379	0,0853	372,8434	77,43954	0,767453	1,303012	372,8434	69,91771	0,958433	11,25108	25,13106	25,13106	1,2101	1,9282	0,982293	1,107581
481	LPS+KYNA	95	1,024	0,0907	425,8777	82,11773	0,770025	1,298659	425,8777	74,6647	0,959984	11,99025	24,77199	25,36989	1,0806	1,6719	0,970252	1,09982
482	LPS+KYNA	96	1,0625	0,0747	486,1111	89,78706	0,727647	1,374292	486,1111	79,10078	0,9763	14,95218	25,79771	26,33663	1,0432	3,022	0,960292	1,135097
483	LPS+KYNA	97	1,0605	0,1976	719,7827	115,9287	0,609558	1,640533	719,7827	100,478	0,895919	17,90241	37,49193	37,49193	1,5566	2,0862	0,905704	1,153772
484	LPS+KYNA	98	1,0501	0,1055	361,6518	85,66082	0,578056	1,729937	361,6518	72,35844	0,868004	11,5261	28,27667	28,27745	1,3727	1,6768	0,933328	1,18384
485	LPS+KYNA	99	1,0783	0,1267	221,3168	64,96011	0,613477	1,630054	221,3168	54,3355	0,942013	9,953936	18,52477	18,61411	1,1821	1,7619	0,930824	1,195537
486	LPS+KYNA	100	1,0309	0,12	225,2779	60,4539	0,755038	1,324436	225,2779	55,09828	0,932507	9,171277	19,03862	19,30365	1,2005	2,0368	0,974742	1,097201
487	LPS+KYNA	101	1,0439	0,131	203,7435	62,95408	0,624688	1,600799	203,7435	56,82599	0,792865	10,58402	23,97227	23,97227	1,8564	4,7301	0,96698	1,107839
488	LPS+KYNA	102	1,0464	0,1119	188,2451	53,03546	0,835391	1,197045	188,2451	49,27466	0,974284	8,505762	16,41388	16,41801	1,1052	2,7343	0,99332	1,076323
489	LPS+KYNA	103	1,0431	0,0951	324,4304	73,46028	0,732205	1,365737	324,4304	65,62411	0,946682	10,79223	23,11365	23,21629	1,2646	1,9696	0,969186	1,11941
490	LPS+KYNA	104	1,0335	0,0858	448,701	87,7633	0,717484	1,39376	448,701	78,40619	0,917204	13,14433	29,29881	29,29881	1,5453	3,4758	0,980052	1,119341
491	LPS+KYNA	105	1,0799	0,2354	571,557	116,6778	0,498102	2,007621	571,557	93,16002	0,82758	15,91383	36,68285	36,8075	1,9446	2,802	0,944117	1,252445
492	LPS+KYNA	106	1,0933	0,284	459,0124	101,7977	0,412213	2,425931	459,0124	84,62897	0,805371	15,89284	29,59718	30,90867	1,261	1,383	0,740566	1,202871
493	LPS+KYNA	107	1,06	0,1131	250,3647	64,65585	0,729357	1,37107	250,3647	57,35933	0,956257	10,06204	19,81775	19,89312	1,1645	2,1561	0,969111	1,127207
494	LPS+KYNA	108	1,094	0,2035	621,0389	114,2564	0,564952	1,770062	621,0389	91,86121	0,924835	14,83566	31,95674	32,52598	1,1386	1,7356	0,945027	1,243794
495	LPS+KYNA	109	1,0267	0,129	191,4202	54,83369	0,793191	1,260731	191,4202	49,83181	0,968688	8,778812	17,54512	17,56665	1,2192	3,7363	0,99146	1,100375
496	LPS+KYNA	110	1,0292	0,1208	195,004	54,9805	0,804251	1,243393	195,004	50,26993	0,969698	9,422518	17,38674	17,38674	1,1595	4,7506	0,992101	1,093705
497	LPS+KYNA	111	1,0422	0,0821	365,047	75,45142	0,795176	1,257583	365,047	70,057	0,934663	12,2945	25,63637	25,63637	1,322	4,372	0,986824	1,077
498	LPS+KYNA	112	1,0389	0,089	330,0576	75,32979	0,712186	1,404128	330,0576	67,94335	0,898474	10,94872	26,42736	26,42736	1,5835	2,2588	0,974379	1,108715
499	LPS+KYNA	113	1,0804	0,1049	363,821	90,97996	0,515112	1,941324	363,821	73,14475	0,854537	12,07973	28,19149	28,19149	1,6079	1,6917	0,932602	1,243834
500	LPS+KYNA	114	1,0347	0,111	224,8378	58,04291	0,829503	1,205541	224,8378	53,63665	0,9821	10,62966	17,66745	17,72777	1,0428	5,9607	0,989094	1,08215
501	LPS+KYNA	115	1,1333	0,1175	469,2923	124,0638	0,308251	3,244114	469,2923	84,29333	0,829978	14,46899	32,14113	32,31743	1,5016	1,7205	0,804528	1,471811
502	LPS+KYNA	116	1,0301	0,1117	255,206	63,38741	0,776832	1,287279	255,206	58,42271	0,939587	9,759131	20,99399	20,99787	1,3099	2,6508	0,973269	1,084979
503	LPS+KYNA	117	1,0832	0,0889	468,0662	99,17908	0,549572	1,819599	468,0662	82,48867	0,864427	14,58918	31,79697	31,79697	1,7245	3,3269	0,919068	1,202336
504	LPS+KYNA	118	1,0386	0,1039	227,1327	58,8383	0,809738	1,234967	227,1327	54,49637	0,961068	9,516011	19,07161	19,07161	1,155	3,4136	0,982145	1,079674
505	LPS+KYNA	119	1,0859	0,1261	213,7091	67,02695	0,56031	1,784727	213,7091	53,32466	0,944444	9,324805	18,69117	18,71004	1,2652	3,0231	0,937335	1,25696
506	LPS+KYNA	120	1,092	0,2844	414,6861	100,308	0,469778	2,128663	414,6861	77,73385	0,8624	12,9995	27,09752	28,50051	1,2667	2,2138	0,907058	1,290403
507	LPS+KYNA	121	1,0492	0,1257	230,6536	62,77163	0,687879	1,453745	230,6536	55,30569	0,947611	10,28142	18,76754	18,91335	1,2154	3,1262	0,935123	1,134994
508	LPS+KYNA	122	1,0562	0,1064	248,3527	64,77021	0,713883	1,400789	248,3527	57,12337	0,956425	9,426773	19,65608	19,66147	1,1638	2,5431	0,95962	1,133865
509	LPS+KYNA	123	1,0471	0,1037	191,3259	53,79805	0,828254	1,207358	191,3259	50,17411	0,955045	8,063103	16,82996	17,27044	1,0697	2,7803	0,997042	1,072227
510	LPS+KYNA	124	1,1189	0,2558	526,7907	122,6885	0,38488	2,59821	526,7907	86,0533	0,893949	14,33413	30,00856	30,79211	1,1767	1,6167	0,875157	1,425727
511	LPS+KYNA	125	1,0743	0,0994	296,1999	72,71844	0,656302	1,523688	296,1999	63,46713	0,924053	11,41009	22,32777	22,42711	1,3494	1,8338	0,932392	1,145765
512	LPS+KYNA	126	1,1277	0,1897	1823,632	276,802	0,246163	4,06235	1823,632	160,0271	0,89487	25,59766	60,96456	60,96456	1,4461	2,1626	0,823027	1,729719
513	LPS+KYNA	127	1,0643	0,088	462,8163	96,01277	0,590874	1,692409	462,8163	83,28848	0,838394	13,66998	33,3847	33,38755	1,7817	2,2606	0,936558	1,152774
514	LPS+KYNA	128	1,1239	0,2141	681,9325	145,1326	0,37606	2,65915	681,9325	94,61137	0,957336	14,94349	32,24074	32,24078	1,1405	1,5397	0,92435	1,533987
515	LPS+KYNA	129	1,0982	0,2519	500,2263	116,9821	0,42017	2,379991	500,2263	84,00088	0,890697	14,27578	30,44952	30,45876	1,3985	1,9999	0,914718	1,392503
516	LPS+KYNA	130	1,0987	0,1004	435,7804	106,9195	0,44088	2,268193	435,7804	80,9031	0,836656	13,22278	31,22028	31,2489	1,5888	1,8558	0,920358	1,321575
517	LPS+KYNA	131	1,0418	0,0865	480,3267	95,62553	0,627465	1,593713	480,3267	84,85638	0,838257	14,33103	34,00975	34,00975	1,7477	2,7914	0,950586	1,12691
518	LPS+KYNA	132	1,0334	0,1013	253,7913	61,40656	0,834045	1,198976	253,7913	57,05652	0,979661	10,12176	18,98158	19,01977	1,0513	2,7978	0,986127	1,076241
519	LPS+KYNA	133	1,0779	0,1182	322,2612	85,80035	0,480496	2,081181	322,2612	69,60133	0,835954	12,36688	26,31112	26,33933	1,3634	1,9516	0,873476	1,23274
520	LPS+KYNA	134	1,1676	0,1153	553,1663	154,5007	0,24957	4,006898	553,1663	86,01158	0,939618	13,79261	31,54434	31,54434	1,2249	1,3898	0,857013	1,796278
521	LPS+KYNA	135	1,1105	0,1096	585,9237	140,1147	0,309184	3,234325	585,9237	94,12734	0,831034	17,56743	37,16644	37,16644	1,6483	2,6545	0,824391	1,488566
522	LPS+KYNA	136	1,0606	0,1187	433,8942	108,3736	0,379111	2,637747	433,8942	87,40433	0,713719	15,63429	36,17195	36,17195	2,076	2,2148	0,816621	1,239911
523	LPS+KYNA	137	1,0414	0,0891	406,0724	94,66862	0,548264	1,823939	406,0724	84,3331	0,717492	13,97512	34,48661	34,48661	2,2016	3,5178	0,962917	1,122556
524	LPS+KYNA	138	1,1665	0,0972	521,352	133,245	0,331606	3,015623	521,352	82,14103	0,971002	14,4923	28,26222	28,27358	1,1539	2,259	0,898637	1,62215
525	LPS+KYNA	139	1,0367	0,0933	368,4422	78,51082	0,73127	1,367483	368,4422	70,53016	0,930742	11,29984	26,40654	26,40959	1,356	1,5481	0,973549	1,113152
526	LPS+KYNA	140	1,0711	0,095	453,4166	97,77535	0,549431	1,820063	453,4166	78,92307	0,914743	14,07261	28,40035	28,40044	1,3299	3,6833	0,921861	1,238869
527	LPS+KYNA	141	1,039	0,1284	222,6372	66,67234	0,591881	1,68953	222,6372	60,16195	0,772971	9,589716	22,77041	23,13684	1,7952	2,4426	0,940412	1,108214
528	LPS+KYNA	142	1,0882	0,1034	426,7894	102,1167	0,466885	2,141856	426,7894	77,03057	0,903851	12,99862	28,47608	28,48376	1,4993	2,1151	0,907778	1,325664
529	LPS+KYNA	143	1,0633	0,0954	330,372	76,88475	0,677923	1,475095	330,372	68,74113	0,878576	11,04718	26,85278	26,85278	1,5752	2,1301	0,965268	1,118468

530	LPS+KYNA	144	1,0649	0,0828	499,8491	97,48156	0,625084	1,599784	499,8491	81,63232	0,942593	13,33824	28,72613	28,85463	1,1741	1,5479	0,94566	1,194154
531	LPS+KYNA	145	1,1367	0,2554	675,2678	151,7707	0,307685	3,24797	675,2678	100,4331	0,841262	17,91379	37,57025	38,25764	1,6292	2,3933	0,835754	1,511162
532	LPS+KYNA	146	1,0347	0,1036	253,7913	63,16188	0,783776	1,275874	253,7913	57,48807	0,965008	9,615195	19,92216	20,04346	1,1491	2,3042	0,980429	1,098695
533	LPS+KYNA	147	1,0717	0,1255	373,1264	98,67748	0,401815	2,488709	373,1264	74,39791	0,847118	12,74509	27,20865	27,24929	1,383	1,9419	0,834443	1,326348
534	LPS+KYNA	148	1,1487	0,1134	305,3795	100,289	0,339397	2,946398	305,3795	68,91291	0,808069	11,30571	25,23094	25,47707	1,5415	2,5969	0,889541	1,455301
535	LPS+KYNA	149	1,0757	0,1237	217,01	66,81915	0,567251	1,762888	217,01	53,75546	0,943722	9,813954	18,24174	18,41855	1,182	3,4552	0,928727	1,243021
536	LPS+KYNA	150	1,0464	0,1231	198,0849	61,78635	0,633726	1,577969	198,0849	56,19076	0,788372	9,911277	23,70323	23,70323	1,8729	4,7445	0,971909	1,099582
537	LPS+KYNA	151	1,0332	0,1214	175,8274	51,0117	0,843023	1,186208	175,8274	47,68004	0,971903	7,915035	16,22025	16,22168	1,1186	2,2896	0,992848	1,069876
538	LPS+KYNA	152	1,0399	0,0837	371,4916	76,73032	0,777613	1,285988	371,4916	69,78074	0,95871	10,81872	24,9402	24,9402	1,1879	1,5728	0,980706	1,099592
539	LPS+KYNA	153	1,0586	0,0758	477,183	89,76933	0,72068	1,387579	477,183	78,42652	0,974919	14,6027	25,73855	26,26596	1,0287	3,2279	0,968509	1,14463
540	LPS+KYNA	154	1,0495	0,1072	365,8958	86,88936	0,567685	1,761539	365,8958	72,19729	0,882115	11,81573	27,92707	27,94785	1,3665	1,7349	0,932125	1,203499
541	LPS+KYNA	155	1,1866	0,2275	1379,301	297,9076	0,160586	6,227211	1379,301	137,9347	0,911006	22,67062	49,59502	49,97706	1,4173	1,4866	0,822245	2,159772
542	LPS+KYNA	156	1,0909	0,2318	627,6407	126,9007	0,445687	2,243725	627,6407	94,76553	0,878253	15,76989	37,26991	37,26991	1,7001	2,0589	0,909992	1,339102
543	LPS+KYNA	157	1,0734	0,2296	604,3459	114,4241	0,53762	1,860051	604,3459	91,0522	0,916039	14,5767	34,17988	34,17988	1,4076	1,6792	0,926862	1,256687
544	LPS+KYNA	158	1,0377	0,0948	280,2299	66,02394	0,797591	1,253775	280,2299	60,86303	0,950641	9,827394	21,68998	21,70771	1,3238	1,8222	0,987323	1,084795
545	LPS+KYNA	159	1,0441	0,0929	410,2221	85,08369	0,656975	1,522127	410,2221	74,05535	0,939973	13,90463	25,98229	25,98229	1,1939	2,5924	0,922599	1,14892
546	LPS+KYNA	160	1,0464	0,1184	185,6672	55,33511	0,746626	1,339358	185,6672	49,70076	0,944536	8,77773	17,80216	17,81018	1,2423	3,7873	0,979851	1,113365
547	LPS+KYNA	161	1,0389	0,1177	233,0429	63,59504	0,694797	1,43927	233,0429	56,89479	0,906385	10,51738	21,86323	21,86323	1,4039	4,4233	0,959531	1,117766
548	LPS+KYNA	162	1,0258	0,1276	172,9981	50,11259	0,866781	1,153694	172,9981	47,07596	0,980963	8,32945	15,66016	15,67553	1,0947	5,5323	1,001272	1,064505
549	LPS+KYNA	163	1,0293	0,1158	206,2271	58,12908	0,749766	1,33375	206,2271	53,86777	0,893094	8,587996	21,10823	21,10823	1,4509	1,8077	0,977591	1,079107
550	LPS+KYNA	164	1,0403	0,0848	330,7806	71,78369	0,784594	1,274544	330,7806	65,91516	0,956707	12,08496	23,1456	23,15238	1,1533	3,0662	0,972629	1,089032
551	LPS+KYNA	165	1,0585	0,1626	1215,576	156,3984	0,587185	1,703042	1215,576	128,2459	0,928763	20,47204	46,38381	46,53745	1,3693	1,5425	0,940259	1,21952
552	LPS+KYNA	166	1,0849	0,2074	571,4313	112,0353	0,531867	1,880171	571,4313	89,61009	0,894252	14,49762	31,8108	32,51626	1,4027	2,2636	0,929691	1,250253
553	LPS+KYNA	167	1,0711	0,2164	585,1378	111,9745	0,529546	1,888411	585,1378	93,03151	0,849586	16,16399	36,24011	36,24011	1,8132	1,8021	0,902971	1,203619
554	LPS+KYNA	168	1,0892	0,2111	788,7556	141,8447	0,421342	2,373369	788,7556	110,1439	0,817017	18,78117	43,14016	43,14016	1,6514	2,0816	0,855281	1,287812
555	LPS+KYNA	169	1,0739	0,0928	468,2549	102,955	0,510372	1,959354	468,2549	80,30988	0,912334	13,86975	30,71216	30,71216	1,3995	2,7356	0,919369	1,281971
556	LPS+KYNA	170	1,0457	0,1085	288,6236	70,64113	0,693015	1,442971	288,6236	61,89055	0,946875	10,22291	21,38346	21,58915	1,1761	2,1421	0,953491	1,141388
557	LPS+KYNA	171	1,0347	0,091	487,18	96,22039	0,628905	1,590065	487,18	81,74348	0,916205	13,34268	29,55945	29,62223	1,3974	1,4808	0,951087	1,177102
558	LPS+KYNA	172	1,0746	0,0894	524,4329	105,6628	0,544313	1,83718	524,4329	86,61754	0,878391	13,66521	33,58326	33,58326	1,5498	2,1912	0,922132	1,219877
559	LPS+KYNA	173	1,0554	0,0853	521,9493	102,6895	0,586741	1,704331	521,9493	87,56677	0,85538	14,29996	35,10548	35,12191	1,674	2,7436	0,943323	1,1727
560	LPS+KYNA	174	1,0467	0,1085	258,0667	66,27465	0,721325	1,386338	258,0667	59,32236	0,92152	10,26706	22,56723	22,56723	1,3997	2,8074	0,976976	1,117195
561	LPS+KYNA	175	1,0557	0,1149	316,8855	81,47677	0,554328	1,803987	316,8855	68,89784	0,838882	11,62686	28,05007	28,05007	1,8127	2,1731	0,924107	1,182574
562	LPS+KYNA	176	1,1395	0,1084	313,1445	96,45337	0,391514	2,554186	313,1445	64,32743	0,950959	10,57222	22,9156	22,93899	1,2412	1,7527	0,92561	1,499413
563	LPS+KYNA	177	1,0416	0,0951	325,4678	71,55816	0,77759	1,286025	325,4678	64,71521	0,976573	10,16674	21,75367	21,7577	1,0812	1,5877	0,973534	1,105739
564	LPS+KYNA	178	1,0657	0,2136	606,4836	106,0961	0,639968	1,562578	606,4836	88,69457	0,968801	14,22504	29,38025	29,45007	1,0655	1,1545	0,94521	1,196196
565	LPS+KYNA	179	1,0886	0,0969	442,4765	104,0723	0,47456	2,107214	442,4765	76,58346	0,948047	12,63619	26,8475	26,85282	1,2252	2,3506	0,924405	1,35894
566	LPS+KYNA	180	1,032	0,1231	249,7359	65,83404	0,694097	1,440721	249,7359	59,81915	0,877021	11,4689	22,53936	22,53936	1,3533	2,417	0,958585	1,100551
567	LPS+KYNA	181	1,0826	0,1127	294,4709	78,42482	0,542944	1,84181	294,4709	63,4777	0,918353	10,39229	21,9264	22,44872	1,1323	1,3838	0,902423	1,235471
568	LPS+KYNA	182	1,0587	0,096	242,0024	62,59947	0,760824	1,314364	242,0024	56,11972	0,965602	9,047411	19,40496	19,43319	1,1411	1,4938	0,980385	1,115463
569	LPS+KYNA	183	1,0537	0,1002	306,7627	75,29415	0,647776	1,543743	306,7627	64,38186	0,930004	11,19365	23,22966	23,23021	1,3106	2,5203	0,952654	1,169493
570	LPS+KYNA	184	1,0671	0,0876	599,5674	120,8041	0,464277	2,153885	599,5674	96,21241	0,813927	16,46317	37,95858	37,95858	1,7692	2,1274	0,899276	1,255598
571	LPS+KYNA	185	1,0513	0,116	242,0967	63,48085	0,715042	1,398519	242,0967	57,38422	0,923875	9,42383	19,33356	19,80293	1,0492	1,5286	0,94715	1,106242
572	LPS+KYNA	186	1,0428	0,1244	129,4263	43,47145	0,86169	1,16051	129,4263	40,68879	0,982387	6,761933	13,56933	13,60605	1,0858	2,626	1,001214	1,068389
573	LPS+KYNA	187	1,0459	0,1241	124,1449	42,42535	0,869371	1,150257	124,1449	39,73968	0,987847	7,112074	12,94447	12,94762	1,0156	4,4201	1,003039	1,067582
574	LPS+KYNA	188	1,0722	0,1231	433,1397	105,0323	0,391978	2,551166	433,1397	83,38108	0,782893	14,69206	34,87638	34,87638	1,875	2,0356	0,794455	1,259665
575	LPS+KYNA	189	1,0588	0,1524	154,6074	53,62305	0,641052	1,559937	154,6074	46,24363	0,908523	9,032518	17,87353	17,87353	1,4833	5,3159	0,94876	1,159577
576	LPS+KYNA	190	1,0347	0,1115	391,0455	96,79326	0,445525	2,244545	391,0455	85,68489	0,669311	15,3303	35,63124	35,63124	2,5356	2,1267	0,849425	1,129642
577	LPS+KYNA	191	1,0754	0,1161	379,1623	91,28103	0,511483	1,955099	379,1623	73,27837	0,887326	12,50512	28,23105	28,23144	1,4819	2,5152	0,894453	1,245675
578	LPS+KYNA	192	1,1423	0,1318	338,1998	109,8142	0,304203	3,287282	338,1998	70,0491	0,866119	11,5131	26,87209	26,87209	1,3743	2,0154	0,863172	1,567675
579	LPS+KYNA	193	1,0292	0,1083	274,7284	66,62926	0,758783	1,3179	274,7284	59,70372	0,968524	10,22262	20,397	20,56296	1,1412	2,4838	0,975741	1,115998
580	LPS+KYNA	194	1,0668	0,1134	309,6549	78,63989	0,59038	1,693825	309,6549	64,2797	0,941759	10,47801	21,91493	22,1911	1,0741	1,8432	0,938274	1,223402
581	LPS+KYNA	195	1,0521	0,125	146,9996	48,00266	0,79584	1,256534	146,9996	43,80465	0,962688	8,575816	15,5352	15,5352	1,2091	8,0018	0,992729	1,095835
582	LPS+KYNA	196	1,0562	0,1496	194,1238	61,18103	0,593769	1,684157	194,1238	51,86796	0,906755	8,098954	19,34901	19,35924	1,2826	1,854	0,911093	1,179553
583	LPS+KYNA	197	1,0458	0,1347	129,5206	44,53528	0,81006	1,234477	129,5206	41,41059	0,94913	6,500975	14,25734	14,36241	1,178	1,9208	0,987136	1,075457
584	LPS+KYNA	198	1,0798	0,1303	201,26	64,50904	0,569589	1,755653	201,26	52,81014	0,906843	8,832128	19,32706	19,33074	1,2344	2,6737	0,937207	1,221528
585	LPS+KYNA	199	1,148	0,1586	450,8387	131,9587	0,237192	4,216	450,8387	81,13227	0,860683	14,29943	28,13401	29,63727	1,2083	1,6864	0,729029	1,626464
586	LPS+KYNA	200	1,07	0,1418	318,6774	90,56454	0,39486	2,532545	318,6774	68,9873	0,841438	12,89709	26,96551	26,96551	1,5257	1,7371	0,808721	1,312771
587	LPS+KYNA	201	1,0555	0,1186	151,8724	49,23121	0,763738	1,309264	151,8724	44,35379	0,970123	7,28828	14,90732	14,96878	1,0879	1,5092	0,969986	1,109966
588	LPS+KYNA	202	1,0236	0,1341	163,4098	51,71348	0,752346	1,329176	163,4098	47,66241	0,903932	7,77906	18,09901	18,09901	1,5833	1,7565	0,9798	1,084995
589	LPS+KYNA	203	1,0521	0,1092	318,5831	76,27199	0,645738	1,548617	318,5831	65,43537	0,93499	11,76516	23,80117	23,8044	1,3024	2,84	0,938326	1,165608
590	LPS+KYNA	204	1,0466	0,0971	298,9035	68,21968	0,789178	1,267142	298,9035	62,54197	0,960279	10,26988	22,11413	22,11413	1,1451	1,7394	0,977808	1,090782
591	LPS+KYNA	205	1,1626	0,2411	667,0628	164,6805	0,260252	3,842428	667,0628	98,5364	0,863342	16,34128	34,33862	35,67927	1,1353	1,3984	0,841981	1,671266
592	LPS+KYNA	206	1,0511	0,1213	217,3872	62,11578	0,673403	1,484994	217,3872	55,07798	0,900508	9,384787	20,92874	20,92874	1,5153	1,5773	0,951121	1,127779
593	LPS+KYNA	207	1,044	0,1286	175,1044	55,11277	0,701679	1,425154	175,1044	50,04606	0,87855	8,594309	19,23167	19,23167	1,6179	2,7127	0,968582	1,101241
594	LPS+KYNA	208	1,0866	0,0888	422,3568	92,56312	0,588291	1,69984	422,3568	76,85988	0,898441	12,87917	28,67356	28,67674	1,4514	1,8	0,949684	1,20431
595	LPS+KYNA	209	1,0358	0,103	196,9531	56,28032	0,773268	1,293213	196,9531	51,97089	0,91633	8,874397	19,29287	19,30004	1,4298	2,3297	0,989625	1,08292
596	LPS+KYNA	210	1,0441	0,1133	306,0711	73,4172	0,657868	1,520063	306,0711	64,57124	0,922473	11,06294	22,94371	22,94374	1,2593	2,4294	0,921939	1,136995
597	LPS+KYNA	211	1,0188	0,1124	203,5549	57,17943	0,773306	1,293149	203,5549	52,80691	0,917296	9,25055	19,71835	19,71835	1,4044	2,6271	0,988417	1,082802
598	LPS+KYNA	212	1,0374	0,1228	167,9367	52,03245	0,772333	1,294778	167,9367	48,44392	0,899242	7,81617	18,7147	18,7147	1,6021	4,444	0,990827	1,074076
599	LPS+KYNA	213	1,088	0,1285	298,1804	85,22021	0,46688	2,141878	298,1804	65,32863	0,877974	10,8375	25,19977	25,19977	1,4694	2,5444	0,904902	1,304485
600	LPS+KYNA	214	1,0941	0,1171	278,0921	79,27039	0,512751	1,950264	278,0921	62,56227	0,892839	10,01067	23,4217	23,4217	1,5032	1,8606	0,921999	1,267064
601	LPS+KYNA	215	1,0394	0,1167	359,4198	97,46986	0,43383	2,305049	359,4198	87,21647	0,593765	15,88628	39,39245	39,39245	3,3626	3,6021	0,912534	1,117563
602	SZR104_	1	1,0441	0,1114	209,5279	58,12908	0,744739	1,342753	209,5279	52,23498	0,965003	8,907713	17,66121	17,67443	1,1028	1,8091	0,955739	1,112838
603	SZR104_	2	1,0499	0,1201	240,3991	65,55496	0,681266	1,467855	240,3991	57,72677	0,906542	10,19957	21,67766	21,68021	1,3226	3,1445	0,969138	1,135608
604	SZR104_	3	1,0414	0,1136	235,0548	62,28794	0,73638	1,357995	235,0548	56,1375	0,937287	9,927074	18,83527	19,68188	1,0406	2,3923	0,967233	1,10956
605	SZR104_	4	1,0589	0,1112	278,3122	73,93652	0,600385	1,665598	278,3122	61,52975	0,923786	11,01865	21,26848	21,51122	1,1134	2,5166	0,938439	1,201639
606	SZR104_	5	1,0744	0,1192	398,0559	96,9445	0,442874	2,257976	398,0559	75,86965	0,868995	13,15945	27,873	27,87415	1,2365	1,4058	0,832096	1,277777
607	SZR104_	6	1,0659	0,1274	201,983	63,22996	0,611245	1,636005	201,983	52,19897	0,931538	9,057926	18,56544	18,73152	1,2545	2,9606	0,962802	1,211326
608	SZR104_	7	1,1946	0,0995	650,1182	171,3543	0,237872	4,203937	650,1182	94,10434	0,922534	15,10715	34,95787	34,95787	1,3311	1,6432	0,854932	1,820896
609	SZR104_	8	1,1017	0,1709	1339,187	201,6429	0,354729	2,81905	1339,187	141,1534	0,844633	24,67418	54,23475	54,23475	1,5365	1,9359	0,857062	1,428538
610	SZR104_	9	1,0737	0,1326	283,5622	90,43546	0,393766	2,539581	283,5622	71,01592	0,706556	12,13819	30,31707	30,31707	2,6321	3,034	0,903769	1,273453
611	SZR104_	10	1,0947	0,1068	272,6221	75,06117	0,576148	1,735666	272,6221	59,56363	0,965624	9,573369	20,0009	20,31463	1,0963	1,4087	0,947532	1,260185
612	SZR104_	11	1,093	0,1099	433,2969	101,5544	0,468047	2,136539	433,2969	76,10856	0,94	14,40863	26,36663	26,36663	1,09	2,4794	0,886527	1,334336
613	SZR104_	12	1,1556	0,2341	690,7349	156,9504	0,308458	3,241931	690,7349	95,8259	0,945268	15,4895	33,5856	33,77706	1,1606	1,279	0,875387	1,63787
614	SZR104_	13	1,0429	0,131	190,8229	58,09344	0,687007	1,455589	190,8229	50,28261	0,948428	8,275408	18,15537	18,15537	1,2321	1,5008	0,966886	1,155339
615	SZR104_	14	1,0241	0,092	316,6025	69,84894	0,797568	1,253811	316,6025	64,90605	0,944395	10,46241	23,18835	23,21638	1,1754	1,8493	0,978056	1,076155
616	SZR104_	15	1,044	0,1274	189,4711	57,62748	0,691024	1,447127	189,4711	51,17661	0,909095	8,94383	19,28798	19,28929	1,364	3,1525	0,963829	1,126051
617	SZR104_	16	1,0236	0,1265	340,589	93,02465	0,443094	2,256859	340,589	82,77303	0,624687	15,96388	37,16644	37,16644	3,1854	3,4831	0,895883	1,123852
618	SZR104_	17	1,0429	0,0865	366,3359	76,08209	0,779181	1,283399	366,3359	70,10002	0,936813	11,47236	24,66069	24,92794	1,2251	2,4609	0,979748	1,085336
619	SZR104_	18	1,0671	0,0921	449,6441	93,16844	0,607659	1,64566	449,6441	77,2872	0,945939	12,66654	27,99959	27,99959	1,281	1,7198	0,93351	1,205483
620	SZR104_	19	1,0733	0,1039	323,7702	78,90851	0,592775	1,68698	323,7702	66,10906	0,930946	11,96865	24,24479	24,24991	1,2832	3,0936	0,907175	1,193611
621	SZR104_	20	1,0683	0,2628	549,1738	123,7982	0,385564	2,593606	549,1738	110,9224	0,560894	22,63913	49,42739	49,42739	3,3753	3,9763	0,85626	1,11608
622	SZR104_	21	1,0403	0,1032	391,6742	86,1727	0,629356	1,588925	391,6742	72,48734	0,936721	11,65041	24,34507	25,25567	1,102	1,4161	0,949514	1,188796
623	SZR104_	22	1,0191	0,0879	451,2789	84,60745	0,762956	1,310692	451,2789	76,68879	0,964252	13,58041	26,25371	26,29363	1,1409	2,9908	0,963079	1,103257
624	SZR104_	23	1,044	0,0902	454,1082	88,65496	0,672212	1,487625	454,1082	77,97463	0,93856	12,58805	27,62433	27,62433	1,1906	1,2946	0,925857	1,136972
625	SZR104_	24	1,0606	0,2535	507,4254	96,61809	0,629663	1,588151	507,4254	81,99546	0,948423	13,75934	28,7234	28,72596	1,1865	1,8507	0,921814	1,178335
626	SZR104_	25	1,111	0,253	628,238	136,5791	0,35348	2,829011	628,238	93,24152	0,908061	15,98603	31,19358	33,21803	1,0581	2,5007	0,835218	1,464788
627	SZR104_	26	1,1169	0,178	818,2435	140,0644	0,473805	2,110574	818,2435	104,4237	0,942961	16,61484	35,07816	35,59683	1,0229	1,3267	0,903988	1,341308
628	SZR104_	27	1,0526	0,0971	427,7011	94,23227	0,545434	1,833401	427,7011	77,6827	0,890638	12,83441	29,87839	29,93605	1,5185	1,9396	0,901139	1,213041
629	SZR104_	28	1,0486	0,1048	394,1263	88,99911	0,549718	1,819113	394,1263	75,39635	0,871253	12,67766	28,61704	28,61966	1,5296	2,9941	0,879158	1,180417
630	SZR104_	29	1,0929	0,2447	599,5989	117,58	0,482659	2,071858	599,5989	89,8816	0,932672	15,21472	31,18199	31,49727	1,2412	1,506	0,885597	1,308165
631	SZR104_	30	1,1243	0,2184	730,7857	146,5732	0,387791	2,578707	730,7857	99,0427	0,93617	16,01608	33,93202	34,97349	1,1698	1,4694	0,90721	1,479899
632	SZR104_	31	1,0313	0,1063	247,8497	61,71809	0,800444	1,249306	247,8497	57,33371	0,947497	9,324344	20,69613	20,69613	1,2926	1,4671	0,978945	1,076471
633	SZR104_	32	1,049	0,1005	338,7342	75,64876	0,710266	1,407924	338,7342	67,13853	0,944332	10,74924	23,23168	23,24009	1,2583	1,8769	0,954896	1,126756
634	SZR104_	33	1,1863	0,3229	653,0104	181,6484	0,175027	5,713395	653,0104	96,13491	0,887907	16,04337	35,84762	35,86872	1,4121	1,4754	0,703784	1,889515
635	SZR104_	34	1,071	0,0921	696,2992	138,864	0,392115	2,55027	696,2992	113,4777	0,679493	19,91457	45,76667	46,07695	2,571	2,1935	0,864147	1,223712

636	SZR104_	35	1,0878	0,2369	1010,67	164,0353	0,35032	2,85453	1010,67	127,8773	0,776662	24,65005	49,99402	49,99402	1,6743	2,0072	0,7422	1,282756
637	SZR104_	36	1,1279	0,2263	591,6453	136,4216	0,371851	2,689247	591,6453	89,25853	0,933193	15,08252	31,44853	31,97559	1,2581	2,0787	0,930818	1,528388
638	SZR104_	37	1,0744	0,2351	549,8654	109,8142	0,529521	1,888499	549,8654	89,89165	0,85512	14,6372	35,89145	35,89145	1,715	2,625	0,924132	1,221628
639	SZR104_	38	1,1111	0,2795	1643,56	238,6527	0,173605	5,760203	1643,56	175,7007	0,669034	33,15645	70,17559	70,17771	2,217	1,2991	0,47874	1,358291
640	SZR104_	39	1,1062	0,0958	382,2117	95,78723	0,49364	2,025769	382,2117	71,10768	0,949906	11,96743	24,68363	24,70973	1,2268	2,0139	0,943	1,347073
641	SZR104_	40	1,0924	0,2184	772,0625	144,9576	0,412727	2,422908	772,0625	110,4269	0,795632	19,1723	45,43748	45,43748	1,8645	2,9876	0,893888	1,312703
642	SZR104_	41	1,0362	0,0792	381,0171	75,84911	0,820849	1,21825	381,0171	70,20486	0,971448	13,49759	23,33161	23,3683	1,0468	5,1379	0,986304	1,080397
643	SZR104_	42	1,0666	0,2107	577,0585	106,0278	0,602313	1,660266	577,0585	89,68741	0,901501	15,4319	31,9764	32,11174	1,2542	2,4062	0,933755	1,182193
644	SZR104_	43	1,1834	0,2006	1081,466	233,4018	0,213564	4,682435	1081,466	123,4195	0,892185	20,23014	46,28339	46,28472	1,345	1,7393	0,85608	1,891126
645	SZR104_	44	1,1288	0,3138	660,7439	158,1138	0,248376	4,026161	660,7439	99,52112	0,838325	16,40154	37,08514	37,25702	1,399	1,5558	0,747835	1,588747
646	SZR104_	45	1,1698	0,23	1743,342	292,0858	0,165476	6,043155	1743,342	159,498	0,861155	27,45885	61,88858	61,88858	1,3792	1,8062	0,644414	1,831282
647	SZR104_	46	1,1475	0,2841	820,8843	180,0578	0,209997	4,761968	820,8843	115,9809	0,766865	19,44129	47,41473	47,41473	1,5942	2,3038	0,660003	1,552479
648	SZR104_	47	1,0755	0,2881	512,5182	107,8512	0,449597	2,224214	512,5182	86,66426	0,857509	13,99809	32,73378	32,73378	1,2156	1,6279	0,811998	1,244472
649	SZR104_	48	1,1003	0,255	899,0682	165,0707	0,313362	3,191198	899,0682	113,4559	0,877705	18,9217	39,59621	40,7255	1,325	1,3965	0,755761	1,454934
650	SZR104_	49	1,0744	0,2065	1124,063	160,7755	0,450072	2,221867	1124,063	124,4192	0,912484	22,02353	45,2864	45,29406	1,3023	2,6792	0,82361	1,292208
651	SZR104_	50	1,1055	0,2615	465,8657	108,5176	0,412458	2,424489	465,8657	83,18101	0,8461	16,15261	31,91489	31,92296	1,449	2,444	0,829678	1,304595
652	SZR104_	51	1,0509	0,1951	999,1323	144,5525	0,538538	1,856879	999,1323	127,7085	0,769826	25,46262	52,77378	52,77378	1,8612	3,8036	0,896262	1,131894
653	SZR104_	52	1,0547	0,0929	560,6798	115,08	0,488375	2,047606	560,6798	92,07787	0,831025	16,61066	36,40456	36,40465	1,7375	2,269	0,91797	1,249811
654	SZR104_	53	1,1215	0,2172	948,7387	170,2147	0,335532	2,980345	948,7387	115,4562	0,894379	21,3305	41,43477	41,8431	1,346	2,5221	0,815401	1,47428
655	SZR104_	54	1,0574	0,2123	1293,54	196,9901	0,319246	3,132385	1293,54	162,3742	0,616531	30,07067	72,34043	72,34043	3,0187	2,6199	0,762121	1,213186
656	SZR104_	55	1,0988	0,1395	452,725	119,1349	0,282708	3,537215	452,725	82,08718	0,844293	14,2166	29,25961	30,11573	1,2193	1,5323	0,705298	1,451322
657	SZR104_	56	1,1304	0,3125	1559,246	229,3855	0,142748	7,00535	1559,246	155,0114	0,81545	27,08637	55,03456	55,57812	1,1073	1,2941	0,383334	1,479798
658	SZR104_	57	1,0415	0,1006	299,0921	69,07589	0,772715	1,294137	299,0921	61,95443	0,979196	11,043	20,57959	20,65206	1,0623	2,8334	0,980975	1,114947
659	SZR104_	58	1,0497	0,175	903,1865	125,8294	0,688421	1,452599	903,1865	110,3385	0,932251	18,20027	39,80881	40,13752	1,354	2,0666	0,960355	1,140395
660	SZR104_	59	1,0566	0,1537	1317,778	161,9757	0,598654	1,670413	1317,778	131,9035	0,951785	22,33516	46,42582	46,43278	1,172	1,9225	0,948471	1,227986
661	SZR104_	60	1,0411	0,1959	698,154	107,8083	0,72279	1,383528	698,154	97,65426	0,91998	15,80566	36,69656	36,74032	1,4133	2,1014	0,957538	1,10398
662	SZR104_	61	1,0497	0,175	903,1865	125,8294	0,688421	1,452599	903,1865	110,3385	0,932251	18,20027	39,80881	40,13752	1,354	2,0666	0,960355	1,140395
663	SZR104_	62	1,0697	0,1198	240,8392	68,23759	0,623409	1,604083	240,8392	56,14894	0,959961	10,11661	19,88663	19,88663	1,2383	3,2049	0,959144	1,215296
664	SZR104_	63	1,0401	0,1172	259,7643	67,7539	0,667281	1,49862	259,7643	58,53576	0,952679	9,659911	20,4355	20,45491	1,254	2,1722	0,9384	1,157479
665	SZR104_	64	1,0577	0,1033	362,8464	83,15337	0,627327	1,594065	362,8464	69,22466	0,951504	12,13461	24,00176	24,00176	1,1228	2,1851	0,951308	1,20121
666	SZR104_	65	1,1148	0,1899	2035,455	301,2668	0,214905	4,653215	2035,455	181,752	0,774306	31,33335	70,27296	70,27296	1,6107	2,5539	0,762568	1,657571
667	SZR104_	66	1,0766	0,114	263,1281	72,69025	0,567766	1,761288	263,1281	60,60372	0,900281	10,36167	22,60413	22,71488	1,48	1,7979	0,907288	1,199435
668	SZR104_	67	1,0852	0,1614	182,3663	65,92004	0,434282	2,302652	182,3663	55,24199	0,750958	10,7347	22,96629	22,96629	2,0435	3,024	0,823479	1,193296
669	SZR104_	68	1,1294	0,2271	1102,183	213,0482	0,266102	3,757961	1102,183	126,8153	0,861231	20,16209	51,1352	51,1352	1,7417	2,1722	0,872048	1,679988
670	SZR104_	69	1,1018	0,1079	351,4033	90,19202	0,496665	2,013431	351,4033	68,85844	0,931325	11,00582	24,34702	24,37436	1,128	1,6709	0,914922	1,309818
671	SZR104_	70	1,1875	0,2356	1594,487	329,6149	0,138951	7,196807	1594,487	164,7531	0,738182	27,36713	69,17734	69,17793	2,4255	2,3973	0,753431	2,00066
672	SZR104_	71	1,0846	0,1062	461,1501	106,6406	0,453471	2,205212	461,1501	80,20844	0,900765	13,50167	30,34454	30,46223	1,5272	1,9454	0,889904	1,329543
673	SZR104_	72	1,1894	0,241	901,8347	208,4027	0,203689	4,90584	901,8347	109,1271	0,951637	18,21723	36,84277	37,38351	1,1524	1,5034	0,78119	1,909725
674	SZR104_	73	1,1668	0,2222	1377,98	263,9246	0,206984	4,831293	1377,98	141,2859	0,867471	23,11018	50,1419	50,62165	1,2387	1,6947	0,832615	1,868019
675	SZR104_	74	1,0904	0,249	524,8416	115,7713	0,443742	2,253562	524,8416	87,50688	0,861298	14,22303	34,61582	34,61582	1,7828	2,7029	0,901767	1,322996
676	SZR104_	75	1,1161	0,2899	660,2409	164,6301	0,22352	4,473873	660,2409	123,2337	0,546327	23,43993	57,19067	57,19067	3,6437	3,8865	0,730169	1,335918
677	SZR104_	76	1,0776	0,1246	269,2269	74,1695	0,547858	1,82529	269,2269	61,58677	0,891976	11,35789	21,5358	22,21115	1,2616	2,3766	0,890822	1,204309
678	SZR104_	77	1,0949	0,2261	542,0691	114,2238	0,471622	2,120341	542,0691	85,35876	0,934906	14,37562	30,23401	30,4267	1,1991	1,6409	0,903323	1,338161
679	SZR104_	78	1,0497	0,0952	406,2296	89,41472	0,591413	1,690865	406,2296	77,24606	0,855516	13,62555	29,89679	29,89679	1,7961	2,0579	0,92625	1,157531
680	SZR104_	79	1,0719	0,1871	1150,313	165,1316	0,468639	2,133838	1150,313	130,563	0,847979	21,75746	48,19695	48,19901	1,3516	1,4348	0,884043	1,264765
681	SZR104_	80	1,1006	0,1805	1051,947	166,2385	0,439375	2,275959	1051,947	117,8904	0,951144	19,97704	40,77243	40,77622	1,1878	1,5993	0,918534	1,41011
682	SZR104_	81	1,0961	0,2141	723,4608	135,1502	0,442792	2,258398	723,4608	102,2113	0,870216	17,0181	36,61809	37,10551	1,1859	1,4491	0,889628	1,322263
683	SZR104_	82	1,0984	0,1773	2161,203	297,3619	0,22702	4,404899	2161,203	175,2741	0,884035	29,23849	62,42193	64,24977	1,2432	1,6804	0,739145	1,696553
684	SZR104_	83	1,0939	0,2712	704,9759	140,2186	0,335429	2,981259	704,9759	101,7351	0,855938	17,47805	39,35532	39,35798	1,4956	2,0545	0,744437	1,378272
685	SZR104_	84	1,0972	0,2134	1450,254	220,0943	0,265516	3,766246	1450,254	157,2423	0,73708	28,64723	61,70238	61,70248	2,1962	1,7473	0,705757	1,399715
686	SZR104_	85	1,1022	0,2019	642,7305	119,9511	0,516207	1,937207	642,7305	92,51972	0,943561	15,60289	31,69348	31,77911	1,0712	1,8159	0,919589	1,296492
687	SZR104_	86	1,0784	0,2329	575,4552	111,3544	0,545527	1,83309	575,4552	87,20034	0,951009	14,15819	30,14862	30,35975	1,0875	1,8333	0,935427	1,276995
688	SZR104_	87	1,0768	0,1044	381,7087	95,46082	0,487744	2,050255	381,7087	73,55046	0,886688	12,08268	27,18264	27,18566	1,5266	1,9961	0,926618	1,297896
689	SZR104_	88	1,1124	0,2128	919,7538	172,4316	0,334041	2,993641	919,7538	116,1681	0,85646	22,32252	45,06372	45,06372	1,6693	2,3121	0,859316	1,484328
690	SZR104_	89	1,1085	0,1949	938,3331	162,409	0,394425	2,535338	938,3331	115,4555	0,88458	19,51445	44,79959	44,79959	1,4714	3,5739	0,882304	1,40668
691	SZR104_	90	1,0779	0,1931	642,982	113,7936	0,591308	1,691165	642,982	95,98848	0,87694	16,05874	33,72759	34,65633	1,3351	2,4053	0,947636	1,185492
692	SZR104_	91	1,1867	0,2635	882,7209	223,1463	0,18076	5,532202	882,7209	115,3165	0,834161	20,5452	47,01223	47,01223	1,8624	4,4316	0,811425	1,935076
693	SZR104_	92	1,0646	0,1179	347,6623	88,66241	0,490933	2,036937	347,6623	72,48796	0,831449	11,77305	28,67356	28,68254	1,8351	1,9757	0,883353	1,223133
694	SZR104_	93	1,2064	0,2693	967,098	241,6082	0,163909	6,100954	967,098	116,9462	0,888603	19,15977	43,96734	43,96734	1,3663	1,527	0,787309	2,065977
695	SZR104_	94	1,0713	0,1192	193,1178	58,89911	0,659344	1,516658	193,1178	51,60782	0,911173	8,72523	17,84098	17,86755	1,1789	2,2034	0,942536	1,141283
696	SZR104_	95	1,0203	0,1151	270,4529	65,00301	0,787781	1,269388	270,4529	60,47454	0,9293	10,0745	21,30613	21,39027	1,18	2,6449	0,979426	1,074882
697	SZR104_	96	1,0739	0,1249	291,9559	78,90851	0,548553	1,822977	291,9559	63,73495	0,901174	10,425	24,78468	24,80119	1,4857	2,3739	0,930979	1,238073
698	SZR104_	97	1,0717	0,0952	731,8545	149,5927	0,359107	2,784685	731,8545	113,611	0,712514	20,7336	46,86656	46,87791	2,3608	1,8881	0,873797	1,31671
699	SZR104_	98	1,1539	0,1092	581,4282	148,0452	0,284679	3,512734	581,4282	92,612	0,851866	14,54012	34,4264	34,99472	1,6451	1,8419	0,853961	1,598553
700	SZR104_	99	1,0521	0,1285	238,5758	67,9617	0,584945	1,709561	238,5758	57,70628	0,900305	10,07629	21,97009	21,97009	1,4273	2,4159	0,901173	1,177718
701	SZR104_	100	1,0973	0,1158	406,1667	111,9924	0,367165	2,723572	406,1667	82,55098	0,748979	12,80977	35,71716	35,71716	2,1787	3,0016	0,902245	1,356645
702	SZR104_	101	1,0688	0,0897	507,897	107,4966	0,525318	1,903608	507,897	81,8981	0,951563	13,77624	28,42691	28,57363	1,2209	2,7751	0,951102	1,312566
703	SZR104_	102	1,1259	0,2463	608,6842	136,5686	0,360906	2,770808	608,6842	89,60285	0,952704	14,95805	31,58468	31,59665	1,1941	2,4083	0,880023	1,524155
704	SZR104_	103	1,146	0,2017	762,2227	153,397	0,362351	2,759755	762,2227	101,3116	0,933196	16,35934	36,10846	36,11241	1,2219	1,6275	0,890167	1,51411
705	SZR104_	104	1,0684	0,2434	510,4434	99,7844	0,584306	1,711432	510,4434	83,08007	0,929316	14,28241	29,0369	29,23617	1,169	1,9625	0,907003	1,201063
706	SZR104_	105	1,0651	0,1492	1247,422	155,256	0,621162	1,609885	1247,422	128,9272	0,943049	21,28426	45,12681	45,32757	1,2173	2,0059	0,955166	1,204215
707	SZR104_	106	1,0491	0,226	677,5627	118,7656	0,569331	1,756448	677,5627	97,02816	0,904406	15,77993	34,19413	34,84059	1,2557	1,941	0,943163	1,224032
708	SZR104_	107	1,0667	0,0971	522,5467	108,8722	0,515194	1,941016	522,5467	89,08686	0,827384	14,4417	36,16195	36,16195	1,5654	2,1803	0,929972	1,22209
709	SZR104_	108	1,0739	0,2075	1052,544	156,2516	0,477142	2,095812	1052,544	118,2306	0,946215	19,11544	40,11679	40,18894	1,1095	1,2194	0,880738	1,321584
710	SZR104_	109	1,0843	0,2017	653,8592	113,2461	0,590263	1,69416	653,8592	93,3992	0,941906	16,76599	32,13576	32,14238	1,1049	2,3139	0,921294	1,212495
711	SZR104_	110	1,0469	0,2012	608,8414	98,12252	0,766339	1,304905	608,8414	89,74456	0,949943	15,2475	31,3925	31,42106	1,2562	1,9894	0,964372	1,093353
712	SZR104_	111	1,073	0,2504	485,8596	100,6982	0,553724	1,805954	485,8596	83,0366	0,885485	13,83869	31,12952	31,20989	1,4498	1,6372	0,919638	1,212697
713	SZR104_	112	1,0979	0,22	702,178	129,3399	0,470078	2,127305	702,178	98,26012	0,913907	16,98447	34,44693	34,82876	1,1071	1,9235	0,891207	1,316301
714	SZR104_	113	1,0343	0,0961	397,7101	89,75443	0,59229	1,688362	397,7101	80,73984	0,766656	13,26943	32,70929	32,70929	1,8679	1,6907	0,954707	1,11165
715	SZR104_	114	1,0695	0,1068	443,9226	97,24858	0,510913	1,957282	443,9226	78,63496	0,902164	12,42826	26,81528	28,18629	1,0507	1,5099	0,866157	1,236709
716	SZR104_	115	1,0667	0,1136	409,4361	105,9239	0,404102	2,47462	409,4361	89,52684	0,641932	16,40594	39,1844	39,1844	2,9841	3,014	0,881219	1,183153
717	SZR104_	116	1,0744	0,24	508,3685	102,8615	0,567082	1,763413	508,3685	83,03493	0,926545	13,40837	30,01642	30,04011	1,15	1,4883	0,939212	1,238774
718	SZR104_	117	1,0505	0,0802	480,0438	93,03936	0,674197	1,483247	480,0438	80,54904	0,929757	13,67188	29,95771	29,95771	1,4439	2,3683	0,967453	1,155065
719	SZR104_	118	1,0657	0,0903	522,5152	104,8349	0,551002	1,814876	522,5152	87,62332	0,855203	15,26151	32,93151	32,96071	1,5865	3,1961	0,922267	1,196427
720	SZR104_	119	1,0188	0,0864	508,4629	91,03333	0,741277	1,349023	508,4629	81,94475	0,951539	13,55816	28,61429	28,64246	1,2269	2,5468	0,96142	1,110911
721	SZR104_	120	1,155	0,1998	767,9128	159,0351	0,33574	2,978498	767,9128	101,7821	0,931491	16,51702	36,35918	36,36647	1,2114	1,6265	0,879969	1,562505
722	SZR104_	121	1,0589	0,148	1248,743	152,4117	0,646213	1,547478	1248,743	128,955	0,94364	21,80582	45,27809	45,53832	1,2257	1,9517	0,956598	1,181899
723	SZR104_	122	1,0655	0,216	683,2843	117,7626	0,578728	1,727926	683,2843	96,51055	0,921852	16,30316	33,45619	34,15638	1,2332	1,5499	0,934714	1,220204
724	SZR104_	123	1,0739	0,2075	1052,544	156,2516	0,477142	2,095812	1052,544	118,2306	0,946215	19,11544	40,11679	40,18894	1,1095	1,2194	0,880738	1,321584
725	SZR104_	124	1,0776	0,2049	654,1421	111,344	0,612164	1,633549	654,1421	93,66099	0,937053	16,36452	32,13576	32,13576	1,1063	2,2995	0,923251	1,188798
726	SZR104_	125	1,0462	0,2013	606,7979	97,88972	0,769453	1,299625	606,7979	89,60367	0,949734	15,27252	31,27461	31,3409	1,2532	2,101	0,966946	1,092474
727	SZR104_	126	1,1194	0,2262	766,5296	142,6222	0,395572	2,527984	766,5296	104,6806	0,879035	19,38069	38,07477	38,20009	1,2509	2,3347	0,835336	1,362451
728	SZR104_	127	1,145	0,2058	836,3199	174,5562	0,292872	3,414462	836,3199	107,7112	0,905858	19,55929	39,11654	39,51388	1,2826	3,1934	0,849115	1,620595
729	SZR104_	128	1,0509	0,0894	411,3538	84,78989	0,683076	1,463965	411,3538	74,80835	0,923687	11,89613	27,10215	27,10215	1,2984	1,6684	0,950019	1,133428
730	SZR104_	129	1,0715	0,119	230,7794	67,32057	0,610087	1,639111	230,7794	55,51309	0,941057	9,341667	20,19098	20,19098	1,221	2,1143	0,953412	1,212697
731	SZR104_	130	1,0716	0,232	500,0377	98,1477	0,621303	1,60952	500,0377	81,82493	0,938515	13,04679	28,48215	28,61186	1,1103	1,618	0,952471	1,199484
732	SZR104_	131	1,0486	0,0923	294,2194	71,23174	0,695429	1,437961	294,2194	65,10271	0,872334	11,53475	24,19222	24,3152	1,5308	2,7576	0,954375	1,094144
733	SZR104_	132	1,0697	0,084	491,4554	99,63741	0,59387	1,683869	491,4554	82,0747	0,916801	13,1602	29,89679	30,04358	1,4567	1,8043	0,954647	1,213984
734	SZR104_	133	1,0575	0,0907	519,2458	103,7711	0,562174	1,778809	519,2458	87,45035	0,853217	14,93239	32,77266	32,88718	1,5877	3,3391	0,927771	1,186629
735	SZR104_	134	1,1342	0,1215	470,204	146,7128	0,237658	4,207734	470,204	82,66397	0,864696	14,29227	31,54085	31,6809	1,5323	2,7904	0,865748	1,774809
736	SZR104_	135	1,142	0,108	908,5308	213,0986	0,195206	5,122787	908,5308	121,2682	0,776346	20,00746	48,22988	48,22988	1,8863	1,8902	0,776436	1,757251
737	SZR104_	136	1,1465	0,1933	1434,661	240,3691	0,271002	3,690008	1434,661	142,8854	0,883046	23,71073	49,74787	51,54355	1,2442	1,5554	0,868503	1,682251
738	SZR104_	137	1,105	0,1805	1151,476	178,1704	0,40695	2,457303	1151,476	126,3959	0,905729	21,19968	43,58855	45,39032	1,175	2,0836	0,892787	1,409621
739	SZR104_	138	1,1617	0,2541	1049,557	224,547	0,189183	5,285888	1049,557	129,0225	0,792291	21,56693	48,66819	48,68131	1,6705	2,0034	0,723237	1,740371
740	SZR104_	139	1,0732	0,1791	1527,086	206,7764	0,389972	2,564285	1527,086	153,1425	0,818242	26,33307	60,67147	60,67826	1,6278	2,7226	0,868886	1,350222
741	SZR104_	140	1,0967	0,1465	2169,314	245,6663	0,407768	2,452374	2169,314	168,7304	0,957514	27,5814	56,72041	56,80383	1,1497	1,6766	0,902761	1,45597

742	SZR104_	141	1,088	0,1851	1305,738	189,8222	0,416062	2,403489	1305,738	139,1487	0,847437	22,97984	52,14725	52,15218	1,3921	2,0419	0,913663	1,364168
743	SZR104_	142	1,1245	0,2487	588,3444	140,3046	0,326027	3,067229	588,3444	94,39911	0,82967	17,67307	38,04917	38,04917	1,9267	2,6545	0,868074	1,486292
744	SZR104_	143	1,057	0,0865	513,1155	98,01862	0,620638	1,611245	513,1155	82,95426	0,937017	13,33615	29,62479	29,69344	1,213	1,3782	0,924764	1,181598
745	SZR104_	144	1,1774	0,2383	1229	264,9707	0,168233	5,944125	1229	139,863	0,789507	24,07041	53,40647	53,52851	1,8086	2,3088	0,764798	1,894502
746	SZR104_	145	1,0901	0,2205	582,4028	113,2387	0,523857	1,908917	582,4028	89,31144	0,917527	14,19566	32,71121	32,71121	1,3188	1,6806	0,917845	1,267908
747	SZR104_	146	1,0467	0,1087	342,5695	83,43972	0,575195	1,738541	342,5695	68,86957	0,907619	11,20248	23,6142	24,6016	1,1382	1,4859	0,930256	1,211561
748	SZR104_	147	1,1147	0,2606	578,3789	129,15	0,389301	2,568709	578,3789	87,46229	0,950124	14,39243	29,88943	30,04234	1,1567	1,6476	0,893412	1,476636
749	SZR104_	148	1,1071	0,1948	1540,541	239,9452	0,264557	3,779905	1540,541	158,1691	0,773819	26,77491	66,87594	66,87594	1,933	2,7588	0,786793	1,517017
750	SZR104_	149	1,0666	0,0936	242,0024	63,74202	0,730682	1,368584	242,0024	56,81174	0,942222	8,917287	20,37388	20,37789	1,1334	1,6452	0,976228	1,121987
751	SZR104_	150	1,1555	0,0991	485,1994	123,5979	0,366204	2,730718	485,1994	80,73773	0,935356	13,18457	29,22521	29,2566	1,2896	1,6821	0,91752	1,530856
752	SZR104_	151	1,0529	0,1061	362,1863	83,83741	0,606172	1,649697	362,1863	70,93576	0,904505	11,61899	26,06443	26,48223	1,4094	2,3476	0,936117	1,181878
753	SZR104_	152	1,0443	0,0987	260,4874	62,86809	0,817006	1,223981	260,4874	58,26628	0,964188	9,38328	20,1161	20,11746	1,1703	2,046	0,986483	1,078979
754	SZR104_	153	1,0994	0,2386	658,4805	126,1589	0,462486	2,162228	658,4805	95,96817	0,898458	16,54796	35,28546	35,47764	1,2741	2,7657	0,889573	1,314591
755	SZR104_	154	1,0864	0,2308	791,2077	144,7394	0,411484	2,43023	791,2077	110,0893	0,82037	18,7203	45,19887	45,19887	1,7852	2,3872	0,867014	1,314745
756	SZR104_	155	1,0746	0,0897	455,9944	94,7516	0,574278	1,741318	455,9944	79,43605	0,9081	14,4061	27,78488	28,17025	1,3438	1,9605	0,899759	1,192804
757	SZR104_	156	1,0843	0,1176	535,9074	122,767	0,373694	2,675987	535,9074	95,00236	0,746157	18,13704	37,90348	37,90645	1,6203	3,032	0,836335	1,292252
758	SZR104_	157	1,0935	0,1191	355,1758	93,72039	0,455419	2,194863	355,1758	68,01037	0,964945	10,89388	23,40761	23,45934	1,1171	2,5246	0,896619	1,378031
759	SZR104_	158	1,0755	0,212	1041,887	168,3619	0,406983	2,457105	1041,887	140,1951	0,666138	23,87133	61,93504	61,93504	2,3757	3,2938	0,881118	1,200912
760	SZR104_	159	1,0998	0,2478	1013,09	185,8502	0,277459	3,604141	1013,09	134,7058	0,701593	23,0761	54,00239	54,02895	1,7851	1,903	0,752777	1,379675
761	SZR104_	160	1,0928	0,173	1117,178	166,9402	0,464847	2,151244	1117,178	130,0388	0,830207	21,79395	47,86906	48,6741	1,3709	2,2492	0,922785	1,283773
762	SZR104_	161	1,1559	0,2177	687,0567	164,9521	0,275788	3,625979	687,0567	99,54782	0,871242	16,54319	37,177	37,177	1,3275	2,3601	0,869137	1,657014
763	SZR104_	162	1,0896	0,2156	1601,655	231,1124	0,292724	3,416185	1601,655	184,6815	0,590108	38,60773	82,76952	82,76952	2,7067	2,278	0,776831	1,25141
764	SZR104_	163	1,1445	0,2008	2540,239	366,8071	0,160471	6,231638	2540,239	196,4443	0,82719	34,03454	77,84677	77,84677	1,6202	2,4213	0,676377	1,867232
765	SZR104_	164	1,1028	0,2792	2116,719	281,7473	0,155409	6,434639	2116,719	191,5962	0,724601	34,7706	77,50356	77,50356	1,9482	1,8259	0,463791	1,470527
766	LPS+SZR10	1	1,0423	0,1679	672,5957	99,5945	0,844497	1,184136	672,5957	92,42699	0,989387	14,44479	30,22152	30,43759	1,0394	2,1585	0,991073	1,077548
767	LPS+SZR10	2	1,0392	0,1024	282,8077	67,55355	0,757379	1,320342	282,8077	60,88232	0,958779	10,88213	20,43628	20,6861	1,0496	3,338	0,972543	1,109576
768	LPS+SZR10	3	1,0942	0,0944	510,6006	117,1468	0,436779	2,289487	510,6006	88,16438	0,825476	15,58417	36,16195	36,16195	1,9984	2,9812	0,934183	1,328732
769	LPS+SZR10	4	1,0696	0,0977	290,7613	71,89486	0,688545	1,452338	290,7613	61,48977	0,966363	9,871454	21,78832	21,78832	1,1845	1,3092	0,974051	1,169217
770	LPS+SZR10	5	1,1822	0,1924	802,8708	178,2566	0,281659	3,550387	802,8708	102,1997	0,965953	16,79378	35,10817	35,10832	1,1314	1,5626	0,887075	1,744198
771	LPS+SZR10	6	1,0417	0,1283	141,341	45,99663	0,834281	1,198637	141,341	42,91248	0,964518	8,089273	15,07823	15,09005	1,236	4,4608	0,993772	1,071871
772	LPS+SZR10	7	1,1123	0,2761	686,3966	155,2959	0,296113	3,377089	686,3966	103,1295	0,810997	18,89085	39,49087	39,49395	1,5697	2,9277	0,827929	1,505835
773	LPS+SZR10	8	1,0976	0,3128	541,4718	122,2048	0,301141	3,320705	541,4718	95,29131	0,74934	16,91005	37,12835	37,16023	1,9057	1,4962	0,660938	1,282434
774	LPS+SZR10	9	1,196	0,1946	727,9563	177,1349	0,259641	3,851468	727,9563	96,78119	0,976636	16,68535	31,7158	32,13294	1,0361	3,0632	0,890568	1,830262
775	LPS+SZR10	10	1,0678	0,1073	410,8508	93,65957	0,521456	1,917706	410,8508	75,83264	0,897803	13,0142	26,75426	26,75452	1,41	1,4029	0,88599	1,235083
776	LPS+SZR10	11	1,069	0,1226	238,733	71,59379	0,517466	1,932494	238,733	59,50427	0,847277	11,84567	19,50355	21,34002	1,0862	2,12	0,884119	1,203171
777	LPS+SZR10	12	1,0345	0,1157	230,4021	60,80124	0,761825	1,312638	230,4021	54,82278	0,963327	9,303564	18,75163	18,93532	1,1795	2,3107	0,972711	1,109051
778	LPS+SZR10	13	1,0715	0,0889	375,547	87,51259	0,595428	1,679465	375,547	71,50385	0,923028	12,32473	27,27732	27,27732	1,4463	3,032	0,966265	1,223886
779	LPS+SZR10	14	1,0331	0,1358	282,1475	78,35355	0,534473	1,871001	282,1475	70,8666	0,705997	12,96642	30,73571	30,73571	2,2929	2,703	0,92546	1,105649
780	LPS+SZR10	15	1,031	0,1292	244,8317	70,10408	0,599738	1,667394	244,8317	65,38839	0,719575	11,09989	26,23934	26,23934	1,9422	2,0268	0,958012	1,072118
781	LPS+SZR10	16	1,0487	0,0999	505,6021	113,4894	0,452257	2,21113	505,6021	98,04587	0,660937	17,12213	42,54285	42,54592	2,7968	3,2941	0,916807	1,157513
782	LPS+SZR10	17	1,0961	0,0989	486,5198	112,6229	0,437628	2,285045	486,5198	80,60631	0,940962	13,41005	28,1742	28,75	1,1809	1,5888	0,907922	1,397197
783	LPS+SZR10	18	1,0954	0,1028	219,745	63,35177	0,654077	1,528873	219,745	54,36105	0,934443	8,782801	19,75101	19,75113	1,2519	1,3873	0,950644	1,165389
784	LPS+SZR10	19	1,0352	0,0878	384,6009	79,86099	0,729981	1,369899	384,6009	71,77278	0,93821	12,64422	25,45917	25,45917	1,1513	2,2536	0,963296	1,112692
785	LPS+SZR10	20	1,0559	0,0905	486,3626	109,0472	0,472214	2,117684	486,3626	90,42145	0,747527	17,0052	37,24459	37,24459	2,3884	1,9148	0,918751	1,205988
786	LPS+SZR10	21	1,0957	0,1274	381,3	100,3541	0,402897	2,482024	381,3	73,03713	0,898233	11,62908	26,07348	26,09124	1,2379	1,4151	0,846813	1,374015
787	LPS+SZR10	22	1,0763	0,1177	371,5859	94,34362	0,470958	2,12333	371,5859	75,28147	0,823934	12,70975	30,04365	30,04365	1,7742	1,756	0,897716	1,253212
788	LPS+SZR10	23	1,1273	0,0889	583,0001	128,509	0,400992	2,493816	583,0001	90,70099	0,890541	16,05202	33,98387	34,16548	1,551	2,3901	0,903909	1,416843
789	LPS+SZR10	24	1,0434	0,1092	205,3154	56,49557	0,79375	1,259842	205,3154	51,88824	0,958281	9,595496	17,88936	18,07679	1,1539	3,4837	0,981932	1,088793
790	LPS+SZR10	25	1,0929	0,0981	390,9197	94,80195	0,507426	1,970729	390,9197	73,4242	0,91121	11,98399	27,2531	27,2531	1,4521	1,4484	0,928347	1,291154
791	LPS+SZR10	26	1,1173	0,1097	471,5243	127,653	0,325052	3,076429	471,5243	84,39207	0,831975	15,81	33,7369	33,7369	1,8806	3,2889	0,893926	1,512619
792	LPS+SZR10	27	1,1488	0,1612	567,5016	188,7197	0,145951	6,851619	567,5016	105,5347	0,640304	19,73528	45,57255	45,57255	2,976	2,2867	0,728894	1,788225
793	LPS+SZR10	28	1,1479	0,2132	1222,587	226,492	0,229889	4,349918	1222,587	135,1077	0,841645	22,89551	52,31427	52,31445	1,7228	1,9573	0,767601	1,676381
794	LPS+SZR10	29	1,0366	0,0921	392,7116	80,4234	0,730801	1,368362	392,7116	71,66438	0,960896	11,97387	24,72181	24,77007	1,1427	2,1073	0,957813	1,122223
795	LPS+SZR10	30	1,1618	0,119	451,9076	129,4082	0,290133	3,44669	451,9076	77,63853	0,942116	12,89484	27,44335	27,46498	1,1896	1,6394	0,855583	1,666803
796	LPS+SZR10	31	1,0946	0,1245	249,9874	77,02411	0,451602	2,21434	249,9874	59,70067	0,881392	10,84638	21,63121	21,87711	1,3937	3,1851	0,852867	1,290172
797	LPS+SZR10	32	1,0618	0,1383	195,1298	61,35319	0,621296	1,609538	195,1298	52,27379	0,897356	8,435869	19,34574	19,3745	1,2942	1,7858	0,953762	1,173689
798	LPS+SZR10	33	1,1392	0,2025	765,5236	153,333	0,367241	2,72301	765,5236	100,731	0,948073	16,25028	34,47977	34,91055	1,1213	1,4756	0,89754	1,522203
799	LPS+SZR10	34	1,0356	0,1614	1305,706	158,6509	0,612128	1,633646	1305,706	139,6646	0,841167	23,97699	53,74943	53,75661	1,4648	2,3021	0,939014	1,135942
800	LPS+SZR10	35	1,1217	0,088	466,4944	110,8677	0,444298	2,25074	466,4944	77,78191	0,968944	12,87718	26,50397	26,55427	1,1722	2,1393	0,931599	1,425366
801	LPS+SZR10	36	1,0697	0,1024	331,3151	82,1	0,582985	1,715311	331,3151	68,44651	0,888686	11,21988	24,81319	25,07098	1,352	2,1364	0,943828	1,199477
802	LPS+SZR10	37	1,0355	0,107	340,9034	80,83883	0,599443	1,668216	340,9034	74,68027	0,76812	13,12897	27,19768	28,3023	1,2879	2,8745	0,914423	1,082466
803	LPS+SZR10	38	1,0836	0,2308	681,9325	131,3711	0,461217	2,168178	681,9325	96,97764	0,911187	17,58798	36,28	36,29621	1,5065	4,2301	0,928868	1,354653
804	LPS+SZR10	39	1,1465	0,1246	300,0981	104,6598	0,312904	3,195866	300,0981	65,28514	0,884798	11,12745	25,73917	25,74163	1,6545	2,1838	0,908862	1,603117
805	LPS+SZR10	40	1,067	0,2133	624,0255	117,3115	0,526522	1,899255	624,0255	99,03011	0,799608	18,5928	40,4473	40,4473	1,9485	2,987	0,92403	1,184605
806	LPS+SZR10	41	1,048	0,2796	475,5483	103,1553	0,518415	1,928955	475,5483	90,58193	0,728318	16,70773	38,102	38,18261	2,4256	2,1716	0,923118	1,138807
807	LPS+SZR10	42	1,1175	0,1313	266,8691	88,05709	0,372222	2,686567	266,8691	61,90691	0,875043	11,85635	23,47734	23,61296	1,487	2,7947	0,860643	1,422411
808	LPS+SZR10	43	1,0309	0,0845	405,7266	79,27358	0,79333	1,26051	405,7266	72,67793	0,965246	13,31663	24,75484	24,78713	1,1117	4,3905	0,97784	1,090752
809	LPS+SZR10	44	1,0229	0,1203	229,9935	61,57872	0,736979	1,356891	229,9935	56,75606	0,897223	10,96748	20,72952	20,72952	1,3064	4,523	0,966922	1,084972
810	LPS+SZR10	45	1,0193	0,0985	284,8826	65,91259	0,810382	1,233986	284,8826	61,20039	0,9558	10,55282	21,55988	21,58495	1,2803	3,4963	0,983447	1,076996
811	LPS+SZR10	46	1,043	0,1094	266,0832	65,62624	0,754269	1,325786	266,0832	59,69895	0,938196	10,21853	20,08248	20,1908	1,0761	1,8304	0,971526	1,099286
812	LPS+SZR10	47	1,061	0,1147	299,2493	83,63262	0,480989	2,079048	299,2493	71,86996	0,728027	12,39211	30,19395	30,19395	2,3669	2,6214	0,894632	1,163666
813	LPS+SZR10	48	1,062	0,0868	478,7548	98,1477	0,597805	1,672787	478,7548	84,2039	0,848513	13,6877	34,43963	34,44489	1,8448	2,1008	0,957187	1,165596
814	LPS+SZR10	49	1,0697	0,2012	715,6016	127,1871	0,506225	1,975405	715,6016	107,3876	0,779781	19,35415	44,29537	44,29537	1,9646	1,9137	0,910644	1,184374
815	LPS+SZR10	50	1,1256	0,2279	748,4847	151,158	0,370814	2,696773	748,4847	100,3191	0,934598	17,61761	36,49426	36,5977	1,4035	2,1321	0,900794	1,506771
816	LPS+SZR10	51	1,0796	0,207	604,1572	109,5562	0,580631	1,722263	604,1572	89,4977	0,947842	14,65004	31,05723	31,07186	1,1585	1,449	0,917942	1,224123
817	LPS+SZR10	52	1,0597	0,104	229,459	60,67216	0,766357	1,304874	229,459	54,7906	0,960511	8,970301	18,79768	19,03257	1,2171	1,7118	0,978353	1,107346
818	LPS+SZR10	53	1,0888	0,0981	390,1652	98,26206	0,467164	2,140575	390,1652	77,01007	0,826729	13,15098	27,70613	28,40645	1,5479	1,7655	0,91999	1,275964
819	LPS+SZR10	54	1,0847	0,1833	778,0356	128,1113	0,553201	1,807662	778,0356	100,7339	0,963514	16,11355	33,9561	34,25853	1,1067	1,2327	0,928644	1,27178
820	LPS+SZR10	55	1,1027	0,2623	488,5003	114,295	0,409766	2,440418	488,5003	80,96277	0,936491	13,74246	27,59699	27,83619	1,1223	1,5548	0,871999	1,411699
821	LPS+SZR10	56	1,0519	0,1011	213,0489	57,39468	0,795459	1,257135	213,0489	52,32408	0,97788	9,763032	17,4814	17,5031	1,0662	4,2014	0,978752	1,096908
822	LPS+SZR10	57	1,1185	0,2097	2182,391	333,3761	0,169764	5,890514	2182,391	202,4139	0,669363	37,22101	84,51106	84,51106	2,4277	2,3819	0,687976	1,647002
823	LPS+SZR10	58	1,026	0,0949	327,6998	71,67979	0,7891	1,267266	327,6998	65,48284	0,960353	11,35076	23,29183	23,29183	1,2723	2,282	0,984555	1,094635
824	LPS+SZR10	59	1,0563	0,1205	149,7347	48,4789	0,77591	1,288809	149,7347	44,55651	0,947784	7,128511	15,42654	15,43434	1,1494	1,5676	0,969137	1,088032
825	LPS+SZR10	60	1,04	0,1066	219,242	58,11117	0,801117	1,248258	219,242	53,87071	0,949353	8,702979	19,34656	19,34656	1,2625	2,1597	0,981933	1,078716
826	LPS+SZR10	61	1,0803	0,2146	577,1214	111,0679	0,544566	1,836324	577,1214	91,31106	0,869821	16,52456	35,51812	35,52096	1,5329	2,7241	0,926299	1,216369
827	LPS+SZR10	62	1,0508	0,1386	299,5951	86,18759	0,464701	2,15192	299,5951	75,36573	0,662821	13,75161	33,8095	33,8095	2,6884	5,3862	0,916894	1,143591
828	LPS+SZR10	63	1,0833	0,1986	969,5501	160,6938	0,426358	2,345447	969,5501	122,0309	0,818163	20,16472	49,17777	49,18372	1,8994	2,2368	0,903635	1,316829
829	LPS+SZR10	64	1,0726	0,1143	245,1461	69,4305	0,607088	1,647207	245,1461	57,56908	0,929514	9,245372	20,21977	20,46282	1,1711	2,16	0,949987	1,206038
830	LPS+SZR10	65	1,0392	0,1012	345,5246	76,98848	0,701224	1,426077	345,5246	67,17016	0,962356	11,33489	23,15849	23,18404	1,106	3,6199	0,957238	1,146171
831	LPS+SZR10	66	1,0331	0,1323	146,088	46,99965	0,808173	1,237358	146,088	44,31603	0,934765	7,317057	16,40814	16,40814	1,3017	1,7559	0,972455	1,060556
832	LPS+SZR10	67	1,0289	0,1065	237,6012	60,51489	0,797207	1,254379	237,6012	55,50833	0,96904	9,02078	18,34486	18,57394	1,0872	1,3547	0,977772	1,090195
833	LPS+SZR10	68	1,0512	0,0881	395,258	83,26773	0,692268	1,444528	395,258	73,52546	0,918787	12,16278	27,22945	27,23466	1,4528	1,9514	0,966356	1,132502
834	LPS+SZR10	69	1,0367	0,1055	317,1684	72,85496	0,726561	1,376348	317,1684	65,39785	0,931907	10,81848	22,49817	22,97037	1,1812	2,0873	0,967588	1,114027
835	LPS+SZR10	70	1,1103	0,1834	943,4259	160,3213	0,420734	2,376801	943,4259	112,0776	0,943799	18,28342	37,84869	38,85073	1,0976	1,3216	0,912163	1,430449
836	LPS+SZR10	71	1,0277	0,0817	440,6846	85,33759	0,749251	1,334667	440,6846	78,82504	0,891269	14,40622	29,99809	29,99809	1,6369	3,9496	0,985305	1,08262
837	LPS+SZR10	72	1,0976	0,1851	723,618	132,6457	0,491148	2,036045	723,618	101,2552	0,886919	16,09697	37,90844	37,97184	1,4561	1,7813	0,950343	1,310014
838	LPS+SZR10	73	1,0511	0,2218	521,635	93,77376	0,696743	1,43525	521,635	84,46512	0,918801	13,66681	29,00711	29,87507	1,1825	1,3234	0,934671	1,110207
839	LPS+SZR10	74	1,1049	0,2548	603,4027	128,0002	0,404283	2,473515	603,4027	90,16837	0,932628	17,20154	31,475	32,05294	1,1814	2,7322	0,873554	1,419569
840	LPS+SZR10	75	1,0775	0,1044	357,8165	87,3656	0,544175	1,837645	357,8165	71,14285	0,888397	11,96963	25,78248	25,79422	1,2284	1,6457	0,923739	1,228031
841	LPS+SZR10	76	1,0576	0,2241	486,1111	94,21454	0,654501	1,527883	486,1111	80,09402	0,952236	13,0705	27,40207	28,03082	1,1645	1,7108	0,951044	1,176299
842	LPS+SZR10	77	1,0367	0,1146	221,2854	59,04592	0,784678	1,274409	221,2854	53,86587	0,958374	9,115195	18,95257	18,95257	1,1983	2,3867	0,983805	1,096166
843	LPS+SZR10	78	1,0385	0,1111	206,1327	56,04433	0,806961	1,239218	206,1327	52,05821	0,955824	9,899894	18,31741	18,31757	1,1409	3,6556	0,978496	1,07657
844	LPS+SZR10	79	1,068	0,2251	495,3536	95,0172	0,660423	1,51418	495,3536	81,32491	0,941191	13,23791	28,88819	29,04129	1,2598	1,8137	0,95786	1,168365
845	LPS+SZR10	80	1,0325	0,099	286,1715	66,53582	0,795004	1,257855	286,1715	61,22144	0,959464	10,01684	20,34145	20,92207	1,0793	2,2958	0,978688	1,086806
846	LPS+SZR10	81	1,0585	0,0909	520,4404	100,906	0,575035	1,739023	520,4404	83,46759	0,938739	13,57259	29,49131	29,58649	1,2637	1,4787	0,895258	1,208925
847	LPS+SZR10	82	1,0328	0,0946	328,8315	73,33121	0,751682	1,33035	328,8315	65,29098	0,969341	10,39977	22,61732	22,61732	1,1117	1,7086	0,978203	1,123145

848	LPS+SZR10	83	1,0558	0,1069	394,7865	96,07358	0,491172	2,035945	394,7865	80,63183	0,76306	13,96989	32,87608	32,91957	2,2103	2,553	0,91384	1,191509
849	LPS+SZR10	84	1,0494	0,0916	459,0438	93,85248	0,597309	1,674176	459,0438	78,85768	0,927632	12,81043	28,73216	28,82878	1,2939	1,4283	0,912067	1,19015
850	LPS+SZR10	85	1,0935	0,2405	534,9957	109,6495	0,473875	2,110262	534,9957	85,70787	0,915205	14,02927	29,38773	29,87913	1,1025	1,4428	0,847456	1,27934
851	LPS+SZR10	86	1,0361	0,1035	287,9948	72,92624	0,662818	1,508709	287,9948	65,54027	0,842515	10,75931	25,87073	25,87073	1,7595	2,7659	0,97402	1,112694
852	LPS+SZR10	87	1,0367	0,1094	221,8513	58,77748	0,78946	1,266689	221,8513	53,66174	0,968148	8,743245	17,8797	18,11963	1,0466	1,6135	0,978319	1,095333
853	LPS+SZR10	88	1,022	0,1053	261,9964	64,93493	0,764044	1,308825	261,9964	60,30977	0,905169	10,06778	23,1456	23,1456	1,529	3,2341	0,978522	1,07669
854	LPS+SZR10	89	1,0565	0,1036	331,0007	80,10142	0,591197	1,691482	331,0007	68,61863	0,883394	12,81754	26,73133	26,73145	1,5566	2,6905	0,911957	1,167342
855	LPS+SZR10	90	1,0692	0,1249	227,1327	69,20798	0,523571	1,909959	227,1327	55,54709	0,925053	10,03934	21,04559	21,04559	1,2876	3,4618	0,878616	1,245933
856	LPS+SZR10	91	1,03	0,1	267,7494	65,69752	0,762244	1,311916	267,7494	59,48284	0,950943	10,45642	20,21587	20,34165	1,0542	3,9201	0,977809	1,104479
857	LPS+SZR10	92	1,05	0,1146	260,4874	69,29397	0,640829	1,560479	260,4874	60,31367	0,899839	9,656418	22,36293	22,36293	1,3922	1,8757	0,940019	1,148893
858	LPS+SZR10	93	1,0428	0,1162	197,4876	56,00142	0,777588	1,286028	197,4876	51,97771	0,918576	9,309734	17,95601	18,53133	1,1202	4,4877	0,982649	1,077412
859	LPS+SZR10	94	1,0893	0,1211	235,4635	72,00922	0,528274	1,892958	235,4635	59,00406	0,849903	10,39885	22,2205	22,30046	1,7153	2,0478	0,925768	1,220411
860	LPS+SZR10	95	1,0557	0,1093	395,1637	96,41028	0,484092	2,065721	395,1637	77,91809	0,817918	12,86135	30,80466	30,95305	1,6319	2,3742	0,906126	1,237329
861	LPS+SZR10	96	1,1228	0,2857	523,0496	122,4732	0,327292	3,055377	523,0496	88,81071	0,833339	16,03569	31,41502	32,50332	1,2887	1,7765	0,746905	1,379037
862	LPS+SZR10	97	1,0357	0,1456	160,9577	56,99699	0,604979	1,652949	160,9577	50,93661	0,779581	8,744592	21,24406	21,24406	1,9258	4,31	0,97168	1,118979
863	LPS+SZR10	98	1,0439	0,1154	293,0247	75,39805	0,609092	1,641788	293,0247	64,49913	0,885128	10,64433	24,0567	24,27332	1,5007	2,267	0,94035	1,168978
864	LPS+SZR10	99	1,1118	0,224	723,4923	144,8002	0,361133	2,769062	723,4923	103,0472	0,856191	17,98395	40,42709	40,42709	1,5338	2,2596	0,832841	1,405183
865	LPS+SZR10	100	1,0723	0,0883	478,3776	99,89557	0,562816	1,776778	478,3776	80,41683	0,929579	13,06096	27,41413	27,89504	1,0729	1,6458	0,934284	1,242222
866	LPS+SZR10	101	1,1074	0,2838	523,2068	111,1718	0,396418	2,522588	523,2068	91,24351	0,789731	16,54961	33,96305	34,21551	1,4006	1,9789	0,745178	1,218408
867	LPS+SZR10	102	1,0232	0,1343	269,2898	73,71099	0,553386	1,807056	269,2898	68,08757	0,729951	12,08785	26,08252	26,23917	1,4712	1,9361	0,888513	1,082591
868	LPS+SZR10	103	1,0763	0,089	609,0614	123,9629	0,435904	2,294083	609,0614	97,90672	0,798446	16,11021	39,72977	39,72977	1,9957	2,2264	0,875194	1,266133
869	LPS+SZR10	104	1,0702	0,1127	193,778	57,56667	0,712989	1,402546	193,778	50,15303	0,968098	9,387376	17,0148	17,21844	1,0869	5,096	0,970311	1,14782
870	LPS+SZR10	105	1,0377	0,1383	234,3003	71,89486	0,528351	1,892683	234,3003	63,91615	0,720711	10,67606	27,80525	27,80525	2,0406	2,9864	0,927546	1,124831
871	LPS+SZR10	106	1,1433	0,1034	495,888	126,4066	0,341408	2,929043	495,888	80,97984	0,950253	13,59301	27,78941	27,78941	1,0996	1,7803	0,875428	1,560963
872	LPS+SZR10	107	1,0435	0,0896	371,303	81,5984	0,677036	1,477027	371,303	73,72748	0,858379	13,44328	28,84573	28,84794	1,6978	3,2519	0,966133	1,106757
873	LPS+SZR10	108	1,0573	0,0953	455,0199	93,52305	0,609067	1,641856	455,0199	79,12495	0,913199	13,84548	29,36901	29,36998	1,4809	3,2185	0,931671	1,181967
874	LPS+SZR10	109	1,0314	0,1044	294,9424	68,60993	0,776281	1,288193	294,9424	62,13138	0,960118	10,78454	22,32706	22,32706	1,2132	3,8399	0,985931	1,104272
875	LPS+SZR10	110	1,0439	0,1227	200,9142	55,56809	0,81164	1,232073	200,9142	51,51241	0,951473	8,171294	18,39363	18,39863	1,2241	2,4981	0,992646	1,078732
876	LPS+SZR10	111	1,0603	0,2248	512,9584	98,4594	0,643905	1,553025	512,9584	85,75934	0,876453	14,39387	33,5083	33,5083	1,7411	2,186	0,968377	1,148089
877	LPS+SZR10	112	1,0741	0,1192	356,0875	92,82128	0,462599	2,161701	356,0875	74,86202	0,798441	12,46977	28,03998	28,57324	1,4426	2,4434	0,890704	1,239898
878	LPS+SZR10	113	1,04	0,0982	407,4242	87,79894	0,630396	1,586305	407,4242	78,77656	0,825016	12,29574	29,55466	29,83521	1,3256	1,9996	0,949151	1,114531
879	LPS+SZR10	114	1,0367	0,1108	233,7659	61,47482	0,752748	1,328466	233,7659	56,81816	0,909948	9,388121	20,77723	20,77739	1,3568	1,8458	0,968397	1,081957
880	LPS+SZR10	115	1,1175	0,1228	288,2149	85,47837	0,457576	2,18543	288,2149	61,97897	0,942838	10,08617	21,77317	21,77317	1,188	1,2611	0,923102	1,379151
881	LPS+SZR10	116	1,0696	0,2229	562,0002	102,6285	0,633572	1,578352	562,0002	85,6172	0,963438	13,75617	28,94202	29,25888	1,1264	1,2778	0,944901	1,198691
882	LPS+SZR10	117	1,0382	0,1037	265,423	64,45124	0,790391	1,265197	265,423	59,36805	0,94633	9,946968	21,73559	21,73559	1,2319	2,4471	0,984366	1,085622
883	LPS+SZR10	118	1,0432	0,1862	768,2272	119,3562	0,659964	1,515235	768,2272	104,8336	0,878412	19,60539	39,80803	39,80954	1,422	5,8155	0,973892	1,13853
884	LPS+SZR10	119	1,0543	0,194	850,9695	131,5032	0,596694	1,6759	850,9695	111,2276	0,864367	18,43418	37,26991	40,76816	1,0855	2,258	0,964941	1,182289
885	LPS+SZR10	120	1,0632	0,1586	727,9249	110,939	0,722149	1,384756	727,9249	96,75422	0,977139	17,36892	32,50537	32,5175	1,1037	3,0233	0,971626	1,146606
886	LPS+SZR10	121	1,0209	0,0938	329,9004	70,23617	0,831962	1,201978	329,9004	65,31484	0,971781	10,54059	23,02576	23,02576	1,2246	2,2274	0,989994	1,075348
887	LPS+SZR10	122	1,0623	0,0926	400,4137	90,77535	0,583645	1,713371	400,4137	78,50782	0,81638	14,49248	30,59218	30,68486	1,8573	3,2166	0,955798	1,156259
888	LPS+SZR10	123	1,0417	0,1109	188,5594	54,76543	0,767772	1,30247	188,5594	50,42594	0,931858	9,219752	18,2908	18,31128	1,3459	6,3249	0,971824	1,086057
889	LPS+SZR10	124	1,0444	0,1068	229,742	59,63351	0,801728	1,247305	229,742	54,53624	0,970687	9,292394	18,89525	18,89525	1,1562	3,7973	0,987548	1,093466
890	LPS+SZR10	125	1,0744	0,1085	232,0054	66,63972	0,621016	1,610265	232,0054	56,3184	0,919194	9,607039	20,29736	20,29736	1,1269	2,5821	0,945935	1,183267
891	LPS+SZR10	126	1,0405	0,2375	494,5677	92,05124	0,708656	1,411122	494,5677	81,63287	0,932671	14,04312	28,83592	28,83677	1,2896	2,3982	0,966184	1,127625
892	LPS+SZR10	127	1,081	0,098	398,3074	89,94131	0,57479	1,739765	398,3074	72,99863	0,939287	12,36152	25,40477	25,43129	1,2125	2,0449	0,928966	1,232096
893	LPS+SZR10	128	1,0441	0,0827	504,7218	95,85106	0,667558	1,497997	504,7218	82,74424	0,926373	13,25099	29,96663	29,96665	1,293	1,6292	0,966988	1,158402
894	LPS+SZR10	129	1,0392	0,0825	436,5663	82,76613	0,787015	1,270623	436,5663	75,50183	0,962376	12,48241	25,65598	25,95695	1,1549	2,6508	0,982718	1,096214
895	LPS+SZR10	130	1,0228	0,095	277,5263	66,21684	0,783941	1,275606	277,5263	61,35766	0,926352	10,09727	21,64718	21,72496	1,1387	2,531	0,985614	1,079194
896	LPS+SZR10	131	1,1446	0,1252	362,6893	110,3128	0,327852	3,050155	362,6893	69,56567	0,94179	11,51638	24,74468	24,74468	1,1852	1,7536	0,875358	1,585736
897	LPS+SZR10	132	1,1765	0,1226	373,9437	120,6972	0,285634	3,500989	373,9437	72,55853	0,892563	12,76099	27,78941	27,8158	1,4606	3,1337	0,885498	1,663446
898	LPS+SZR10	133	1,1646	0,0996	641,8188	165,6479	0,253377	3,946685	641,8188	100,628	0,796497	16,97686	39,53741	39,73186	1,91	2,1421	0,86202	1,64614
899	LPS+SZR10	134	1,1763	0,2196	799,0669	179,4567	0,25856	3,867576	799,0669	104,2734	0,923519	17,29543	35,6577	36,33245	1,2229	1,4712	0,829255	1,721022
900	LPS+SZR10	135	1,0719	0,1199	229,0503	67,76135	0,590389	1,6938	229,0503	56,78961	0,892489	9,13227	20,14188	20,21832	1,1798	1,7514	0,941806	1,1932
901	LPS+SZR10	136	1,0335	0,118	260,7389	66,94823	0,705737	1,416958	260,7389	59,48351	0,926024	10,00316	21,83805	21,95761	1,4238	3,101	0,965397	1,125492
902	LPS+SZR10	137	1,044	0,0961	353,6039	75,58794	0,757735	1,319723	353,6039	67,65066	0,970918	11,18168	23,25129	23,37355	1,1585	2,1917	0,974307	1,117328
903	LPS+SZR10	138	1,0758	0,1163	215,6896	62,10532	0,679161	1,472405	215,6896	54,30681	0,919031	9,173387	20,0127	20,0127	1,3643	2,6843	0,966477	1,143601
904	LPS+SZR10	139	1,1782	0,2811	649,961	186,144	0,182694	5,473634	649,961	106,5739	0,71911	17,20254	44,16638	44,16638	1,8361	2,3245	0,775042	1,746619
905	LPS+SZR10	140	1,0791	0,1092	350,8375	86,98582	0,536772	1,862989	350,8375	67,68576	0,962323	10,99853	22,91697	22,91812	1,1183	1,4917	0,921237	1,285142
906	LPS+SZR10	141	1,0641	0,1176	338,6399	84,82252	0,551488	1,813278	338,6399	67,11225	0,944809	10,6609	23,85394	23,85404	1,2163	1,4304	0,932417	1,26389
907	LPS+SZR10	142	1,1126	0,2422	585,9866	124,1426	0,423775	2,359743	585,9866	89,65069	0,916199	14,33771	33,29229	33,29229	1,3396	1,7886	0,88691	1,384736
908	LPS+SZR10	143	1,1073	0,108	455,5229	113,0739	0,38514	2,596459	455,5229	80,83805	0,875968	14,91507	29,58603	29,86574	1,4149	2,6588	0,860248	1,398771
909	LPS+SZR10	144	1,1077	0,0971	578,9447	128,38	0,399785	2,501343	578,9447	89,61904	0,905829	14,72766	33,3847	33,40426	1,494	2,3345	0,90568	1,432508
910	LPS+SZR10	145	1,0563	0,1089	258,7269	67,69309	0,673653	1,484444	258,7269	58,41298	0,952867	9,436312	19,36199	20,00794	1,0126	1,4553	0,949453	1,158871
911	LPS+SZR10	146	1,1796	0,2001	1433,75	273,0555	0,207609	4,816736	1433,75	140,1346	0,917471	23,68206	48,18032	48,79603	1,1354	1,5221	0,859144	1,948524
912	LPS+SZR10	147	1,122	0,2697	554,3924	131,4215	0,333851	2,995349	554,3924	88,80699	0,883349	14,69021	33,23746	33,23746	1,4755	1,481	0,827672	1,479855
913	LPS+SZR10	148	1,0974	0,0973	478,189	109,1376	0,46042	2,171928	478,189	81,41794	0,906502	14,11037	30,433	30,50681	1,4427	2,0759	0,912629	1,340461
914	LPS+SZR10	149	1,1119	0,1167	364,2611	98,33759	0,43062	2,322234	364,2611	70,33303	0,925345	11,37649	25,27576	25,27817	1,2941	1,8306	0,909726	1,398171
915	LPS+SZR10	150	1,0524	0,0829	403,1173	82,99167	0,710647	1,407169	403,1173	73,74277	0,931541	12,09837	24,95722	25,31034	1,1729	2,4048	0,966233	1,125421
916	LPS+SZR10	151	1,0724	0,1004	226,8183	66,90816	0,599364	1,668434	226,8183	58,04649	0,845932	9,94883	21,36285	21,38021	1,5962	2,7134	0,941372	1,152665
917	LPS+SZR10	152	1,0496	0,1144	151,0236	47,24309	0,855444	1,168984	151,0236	44,18106	0,972259	8,786348	15,00821	15,03768	1,0992	9,1842	1,006037	1,069306
918	LPS+SZR10	153	1,0289	0,1231	207,8303	57,27287	0,784274	1,275065	207,8303	52,70066	0,940343	8,884025	18,30367	18,33394	1,1996	2,1887	0,985025	1,086758
919	LPS+SZR10	154	1,2216	0,2311	1110,734	269,183	0,155747	6,420654	1110,734	123,2872	0,918298	19,50496	43,9845	43,9845	1,1913	1,3635	0,808531	2,183381
920	LPS+SZR10	155	1,0554	0,1347	272,7164	80,49894	0,482954	2,07059	272,7164	67,66177	0,748573	11,8431	28,64066	28,65245	2,3752	3,5212	0,913199	1,189725
921	LPS+SZR10	156	1,1118	0,1884	1261,82	192,3445	0,374233	2,672129	1261,82	130,5991	0,929665	22,04582	46,03275	46,36294	1,2901	2,0057	0,873163	1,472785
922	LPS+SZR10	157	1,0305	0,0995	232,8228	58,37234	0,860398	1,162253	232,8228	55,09665	0,963795	9,463369	19,50677	19,51445	1,2861	4,7735	1,002025	1,059454
923	LPS+SZR10	158	1,0187	0,1013	274,3826	63,53422	0,851931	1,173803	274,3826	59,81032	0,96386	11,23665	20,80521	20,80521	1,1481	8,62	0,997365	1,062262
924	LPS+SZR10	159	1,1183	0,109	391,9571	103,0085	0,421753	2,371055	391,9571	72,63894	0,933489	11,66459	26,58392	26,67525	1,3561	2,2736	0,908566	1,418089
925	LPS+SZR10	160	1,0923	0,1091	312,9244	82,85213	0,521516	1,917485	312,9244	63,67881	0,969748	10,85145	21,81644	21,81644	1,1137	2,3529	0,910388	1,301094
926	LPS+SZR10	161	1,0526	0,1141	224,6492	63,80284	0,630402	1,58629	224,6492	56,81498	0,874558	9,646064	20,18865	20,46312	1,1875	1,7193	0,90904	1,122993
927	LPS+SZR10	162	1,111	0,2507	488,5003	119,2996	0,359065	2,785012	488,5003	90,73273	0,74567	14,81482	38,77429	38,77429	2,2114	2,7247	0,832486	1,314847
928	LPS+SZR10	163	1,0505	0,1361	150,4577	50,58883	0,705591	1,417251	150,4577	45,747	0,903439	7,732695	15,54328	16,21546	1,1944	3,4447	0,955077	1,105839
929	LPS+SZR10	164	1,1108	0,1071	402,1427	102,2562	0,441545	2,264775	402,1427	73,53333	0,93459	11,65025	26,63413	26,64358	1,3866	1,9215	0,913618	1,39061
930	LPS+SZR10	165	1,0517	0,0913	426,9466	87,78848	0,661917	1,510764	426,9466	75,70748	0,936064	12,51206	27,17456	27,17456	1,259	2,3156	0,950814	1,159575
931	LPS+SZR10	166	1,0909	0,0975	420,4077	101,2993	0,476683	2,097832	420,4077	80,87598	0,807683	14,21333	31,44853	31,46133	1,6436	2,9575	0,925895	1,252526
932	LPS+SZR10	167	1,0971	0,1417	494,5991	131,9617	0,243102	4,113503	494,5991	87,7744	0,806728	15,90949	32,79567	32,81294	1,8319	1,7836	0,681116	1,503419
933	LPS+SZR10	168	1,1341	0,1309	282,0532	89,4828	0,404415	2,472708	282,0532	61,05165	0,950924	9,884255	20,40239	20,88628	1,036	1,9126	0,91362	1,46569
934	LPS+SZR10	169	1,0477	0,1427	165,7047	55,17039	0,660888	1,513117	165,7047	49,54539	0,848277	8,521082	17,977	18,27606	1,4829	3,3138	0,966041	1,113532
935	LPS+SZR10	170	1,0291	0,1318	214,0549	60,5578	0,698265	1,432122	214,0549	55,79918	0,863931	9,730585	22,52051	22,52051	1,4617	3,5219	0,951975	1,085281
936	LPS+SZR10	171	1,037	0,1524	136,2482	49,22074	0,684864	1,460144	136,2482	44,95401	0,847234	7,393865	17,4481	17,50158	1,587	3,8945	0,969082	1,094913
937	LPS+SZR10	172	1,0483	0,1112	232,3512	62,0727	0,727039	1,375442	232,3512	54,65966	0,977284	9,320177	18,49165	18,49165	1,0684	2,5073	0,95941	1,135622
938	LPS+SZR10	173	1,1274	0,113	443,0109	117,2181	0,343553	2,91076	443,0109	78,88463	0,894621	13,91773	29,0764	29,22158	1,4782	2,1341	0,847928	1,485943
939	LPS+SZR10	174	1,1447	0,2458	649,6466	154,4282	0,294928	3,39066	649,6466	99,69387	0,821391	17,0823	40,87793	40,87793	1,7106	2,185	0,861553	1,549024
940	LPS+SZR10	175	1,0386	0,1241	266,1461	74,82837	0,533173	1,875564	266,1461	65,44768	0,780802	11,64883	25,62348	25,62406	1,7774	1,5981	0,892629	1,143331
941	LPS+SZR10	176	1,1158	0,2581	612,8653	139,4307	0,345611	2,893424	612,8653	96,35378	0,829539	16,60101	34,3903	35,37011	1,5309	1,4392	0,872429	1,44707
942	LPS+SZR10	177	1,1098	0,1485	403,3059	126,1736	0,259441	3,854442	403,3059	79,97647	0,792355	13,1117	32,57638	32,57638	1,9488	3,5175	0,814951	1,577634
943	LPS+SZR10	178	1,0397	0,1109	205,3783	55,78635	0,812029	1,231482	205,3783	51,31771	0,980007	8,343032	16,74007	16,9584	1,0325	2,7266	0,979183	1,087078
944	LPS+SZR10	179	1,1046	0,2177	894,3841	167,6066	0,352398	2,837703	894,3841	120,3055	0,776538	22,87936	48,19369	48,19369	1,8456	2,3205	0,880808	1,393174
945	LPS+SZR10	180	1,0635	0,1423	221,254	69,57004	0,528012	1,893895	221,254	56,88667	0,859171	10,00557	20,61622	21,19874	1,3442	5,5283	0,919153	1,222959
946	LPS+SZR10	181	1,0803	0,1196	472,3102	114,206	0,376876	2,653394	472,3102	81,88268	0,885223	15,32009	29,2123	29,96874	1,3918	1,8187	0,828208	1,394752
947	LPS+SZR10	182	1,0901	0,1717	255,4889	82,40106	0,361191	2,768615	255,4889	61,82647	0,83991	11,34523	23,89477	23,89477	1,3899	1,9895	0,763874	1,33278
948	LPS+SZR10	183	1,0311	0,1106	216,004	57,43759	0,816185	1,225213	216,004	53,16344	0,960384	8,712606	18,90191	18,90301	1,2884	2,4894	0,991995	1,080396
949	LPS+SZR10	184	1,1006	0,2564	663,3218	145,8819	0,342135	2,922824	663,3218	105,729	0,745668	18,06273	44,64215	44,64215	2,2565	2,2262	0,873507	1,379772
950	LPS+SZR10	185	1,2269	0,2351	813,025	208,1089	0,182632	5,475483	813,025	103,8905	0,946589	16,95206	34,75358	35,17661	1,0863	1,2745	0,774186	2,003155
951	LPS+SZR10	186	1,0447	0,1021	237,3497	60,71525	0,798277	1,252698	237,3497	56,11232	0,947287	8,733848	19,00144	19,26261	1,1883	2,8119	0,986623	1,082031
952	LPS+SZR10	187	1,0399	0,0864	407,1727	80,69929	0,761724	1,312811	407,1727	73,61863	0,944088	12,50612	25,85798	25,88122	1,2584	2,6918	0,969503	1,09618
953	LPS+SZR10	188	1,0906	0,1062	481,8671	114,0266	0,420813	2,376355	481,8671	81,87167	0,903378	13,97539	28,78736	29,73032	1,2234	2,6984	0,903575	1,392748

954	LPS+SZR10	189	1,0554	0,1105	219,0219	59,49397	0,754822	1,324815	219,0219	53,87032	0,948414	8,832411	19,17846	19,19677	1,2142	1,6722	0,970719	1,104392
955	LPS+SZR10	190	1,1204	0,1125	247,8811	81,57323	0,442058	2,262148	247,8811	61,25683	0,830126	10,11066	25,25585	25,25585	1,8884	2,6383	0,944325	1,331659
956	LPS+SZR10	191	1,1409	0,2539	749,9308	161,317	0,302824	3,302248	749,9308	100,8626	0,926339	17,25807	36,76759	36,76759	1,1711	1,9149	0,836219	1,599373
957	LPS+SZR10	192	1,1124	0,1481	257,8781	89,32128	0,319128	3,133543	257,8781	72,97234	0,608566	11,15894	33,20908	33,20908	2,721	3,2981	0,785688	1,224043
958	LPS+SZR10	193	1,0207	0,1047	334,2073	74,43812	0,73042	1,369076	334,2073	68,39422	0,897814	11,4052	26,06443	26,06443	1,5226	2,8661	0,963691	1,088369
959	LPS+SZR10	194	1,1271	0,2373	523,0182	118,6333	0,418126	2,391622	523,0182	83,58615	0,940714	14,37589	30,09906	30,20949	1,2153	2,0676	0,895354	1,419294
960	LPS+SZR10	195	1,0631	0,0996	351,0261	81,41596	0,631382	1,583827	351,0261	69,28459	0,918916	10,97883	25,10729	25,29631	1,3804	1,8806	0,948773	1,175095
961	LPS+SZR10	196	1,1283	0,2383	979,3899	185,9466	0,293841	3,403205	979,3899	119,489	0,862004	18,8331	47,01926	47,01926	1,4915	1,8829	0,825512	1,556182
962	LPS+SZR10	197	1,0281	0,0952	454,3597	92,10461	0,640498	1,561286	454,3597	81,98277	0,849502	13,52965	32,50683	32,50722	1,732	2,8529	0,951636	1,123463
963	LPS+SZR10	198	1,1605	0,2467	701,2977	175,0798	0,24425	4,094168	701,2977	100,9463	0,86483	16,86546	38,28514	38,28516	1,6336	2,0868	0,849561	1,734385
964	LPS+SZR10	199	1,1671	0,2224	1180,084	274,1771	0,165097	6,057038	1180,084	139,6211	0,760712	25,35888	58,03915	58,03915	2,2676	1,6963	0,836912	1,963722
965	LPS+SZR10	200	1,1422	0,2227	796,2062	172,3468	0,300123	3,331966	796,2062	101,9527	0,962582	16,66035	35,04768	35,04768	1,1803	1,62	0,890986	1,690459
966	LPS+SZR10	201	1,1612	0,2674	1033,336	219,6654	0,189015	5,290596	1033,336	120,7554	0,890509	20,11284	43,8302	43,83069	1,1679	1,3799	0,702373	1,819095
967	LPS+SZR10	202	1,2274	0,249	801,0475	235,0113	0,150151	6,659974	801,0475	102,8172	0,952218	16,58059	34,34504	35,10819	1,032	1,5347	0,82383	2,28572
968	LPS+SZR10	203	1,1358	0,2247	539,5227	131,2924	0,34929	2,862953	539,5227	84,66887	0,94574	14,25301	29,19131	29,19223	1,1211	1,9583	0,888067	1,550657
969	LPS+SZR10	204	1,0817	0,0932	366,6818	86,41312	0,580414	1,722907	366,6818	70,39819	0,929771	11,46904	24,42693	24,43287	1,1808	1,4499	0,940586	1,227491
970	LPS+SZR10	205	1,075	0,1111	366,2416	98,88528	0,426509	2,344615	366,2416	78,9945	0,737536	14,70676	32,92387	32,92387	2,4249	3,3094	0,90618	1,2518
971	LPS+SZR10	206	1,0635	0,092	475,454	98,53067	0,578273	1,729287	475,454	80,58475	0,920052	13,02131	29,98184	29,98184	1,4002	1,6997	0,939632	1,222696
972	LPS+SZR10	207	1,04	0,1147	201,9516	57,29805	0,743996	1,344092	201,9516	51,62176	0,952338	8,859273	17,5281	17,64005	1,1578	2,2809	0,962484	1,109959
973	LPS+SZR10	208	1,0549	0,114	223,4546	60,88723	0,735378	1,359844	223,4546	54,07472	0,960307	8,807855	19,37498	19,37498	1,1843	1,7209	0,970878	1,125983
974	LPS+SZR10	209	1,0555	0,1223	188,025	56,76401	0,702277	1,42394	188,025	51,15245	0,903009	9,210301	17,80569	18,10222	1,1294	2,3382	0,957699	1,109703

Supplementary Table S2

Mann-Whitney U Test Post-hoc analysis of multiple comparisons

	n-1	n-2	N	Median-1	Median-2	Q1-1	Q1-2	Q3-1	Q3-2	Mean-1	Mean-2	SD-1	SD-2	SEM-1	SEM-2	Z-score	p-value	adjusted p-value	U1-value	U2-value	Kruskal-Wallis H-test
Area																					Kruskal-Wallis H statistic=175,78900983519304, p-value=4,242269272588465e-36
SZR104-LPS+SZR104	164	209	373	581,91552	384,60088	397,9695	257,8781	904,5225	523,0496	710,7885	443,1239	445,7885	265,4401	34,91685	18,40496	-7,03774	1,95E-12	2,93E-11	24412,5	9863,5	
LPS+KYNA-SZR104	215	164	379	318,58307	581,91552	239,1259	397,9695	456,8589	904,5225	365,5611	710,7885	197,133	445,7885	13,47573	34,91685	-9,64628	5,10E-22	7,65E-21	7436,5	27823,5	
LPS+KYNA-LPS+SZR104	215	209	424	318,58307	384,60088	239,1259	257,8781	456,8589	523,0496	365,5611	443,1239	197,133	265,4401	13,47573	18,40496	-3,11409	1,85E-03	2,77E-02	18538,5	26396,5	
KYNA-LPS+KYNA	107	215	322	231,34525	318,58307	193,4793	239,1259	328,973	456,8589	290,9235	365,5611	186,1807	197,133	18,08347	13,47573	-4,81952	1,44E-06	2,16E-05	7709,5	15295,5	
KYNA-SZR104	107	164	271	231,34525	581,91552	193,4793	397,9695	328,973	904,5225	290,9235	710,7885	186,1807	445,7885	18,08347	34,91685	-10,4134	2,15E-25	3,23E-24	2206	15342	
KYNA-LPS+SZR104	107	209	316	231,34525	384,60088	193,4793	257,8781	328,973	523,0496	290,9235	443,1239	186,1807	265,4401	18,08347	18,40496	-6,85266	7,25E-12	1,09E-10	5914	16449	
LPS-KYNA	131	107	238	339,52015	231,34525	232,2412	193,4793	459,2953	328,973	373,6214	290,9235	191,3276	186,1807	16,78054	18,08347	-4,60475	4,13E-06	6,19E-05	9442	4575	
LPS-LPS+KYNA	131	215	346	339,52015	318,58307	232,2412	239,1259	459,2953	456,8589	373,6214	365,5611	191,3276	197,133	16,78054	13,47573	-0,47481	6,35E-01	1,00E+00	14511,5	13653,5	
LPS-SZR104	131	164	295	339,52015	581,91552	232,2412	397,9695	459,2953	904,5225	373,6214	710,7885	191,3276	445,7885	16,78054	34,91685	-8,22975	1,88E-16	2,81E-15	4750,5	16733,5	
LPS-LPS+SZR104	131	209	340	339,52015	384,60088	232,2412	257,8781	459,2953	523,0496	373,6214	443,1239	191,3276	265,4401	16,78054	18,40496	-2,37627	1,75E-02	2,62E-01	11593	15786	
CONTROL-LPS	148	131	279	288,16772	339,52015	199,2559	232,2412	448,7718	459,2953	356,0807	373,6214	219,567	191,3276	18,10958	16,78054	-1,89415	5,82E-02	8,73E-01	8419,5	10968,5	
CONTROL-KYNA	148	107	255	288,16772	231,34525	199,2559	193,4793	448,7718	328,973	356,0807	290,9235	219,567	186,1807	18,10958	18,08347	-2,46716	1,36E-02	2,04E-01	9352,5	6483,5	
CONTROL-LPS+KYNA	148	215	363	288,16772	318,58307	199,2559	239,1259	448,7718	456,8589	356,0807	365,5611	219,567	197,133	18,10958	13,47573	-1,67337	9,43E-02	1,00E+00	14265,5	17554,5	
CONTROL-SZR104	148	164	312	288,16772	581,91552	199,2559	397,9695	448,7718	904,5225	356,0807	710,7885	219,567	445,7885	18,10958	34,91685	-9,10488	8,64E-20	1,30E-18	4891	19381	
CONTROL-LPS+SZR104	148	209	357	288,16772	384,60088	199,2559	257,8781	448,7718	523,0496	356,0807	443,1239	219,567	265,4401	18,10958	18,40496	-4,07963	4,51E-05	6,77E-04	11546,5	19385,5	
Perimeter																					Kruskal-Wallis H statistic=153,03597366611822, p-value=3,0140820327179816e-31
SZR104-LPS+SZR104	164	209	373	116,67562	89,321277	90,88387	67,76135	164,184	118,6333	134,1879	100,8863	60,35826	45,79642	4,727623	3,175411	-6,21244	5,22E-10	7,83E-09	23559,5	10716,5	
LPS+KYNA-SZR104	215	164	379	79,27039	116,67562	64,21667	90,88387	101,3481	164,184	86,91958	134,1879	32,54659	60,35826	2,224839	4,727623	-9,01931	1,89E-19	2,84E-18	8099	27161	
LPS+KYNA-LPS+SZR104	215	209	424	79,27039	89,321277	64,21667	67,76135	101,3481	118,6333	86,91958	100,8863	32,54659	45,79642	2,224839	3,175411	-2,98448	2,84E-03	4,26E-02	18702	26233	
KYNA-LPS+KYNA	107	215	322	65,253901	79,27039	55,10381	64,21667	83,45754	101,3481	73,40954	86,91958	27,5927	32,54659	2,68004	2,224839	-4,66004	3,16E-06	4,74E-05	7835	15170	
KYNA-SZR104	107	164	271	65,253901	116,67562	55,10381	90,88387	83,45754	164,184	73,40954	134,1879	27,5927	60,35826	2,68004	4,727623	-10,1343	3,89E-24	5,84E-23	2382	15166	
KYNA-LPS+SZR104	107	209	316	65,253901	89,321277	55,10381	67,76135	83,45754	118,6333	73,40954	100,8863	27,5927	45,79642	2,68004	3,175411	-6,60546	3,96E-11	5,94E-10	6104	16259	
LPS-KYNA	131	107	238	83,586525	65,253901	65,31472	55,10381	100,2844	83,45754	86,48892	73,40954	29,37002	27,5927	2,575921	2,68004	-4,4505	8,57E-06	1,29E-04	9360,5	4656,5	
LPS-LPS+KYNA	131	215	346	83,586525	79,27039	65,31472	64,21667	100,2844	101,3481	86,48892	86,91958	29,37002	32,54659	2,575921	2,224839	-0,14073	8,88E-01	1,00E+00	14210	13955	
LPS-SZR104	131	164	295	83,586525	116,67562	65,31472	90,88387	100,2844	164,184	86,48892	134,1879	29,37002	60,35826	2,575921	4,727623	-8,11436	4,88E-16	7,33E-15	4834,5	16649,5	
LPS-LPS+SZR104	131	209	340	83,586525	89,321277	65,31472	67,76135	100,2844	118,6333	86,48892	100,8863	29,37002	45,79642	2,575921	3,175411	-2,52819	1,15E-02	1,72E-01	11459	15920	
CONTROL-LPS	148	131	279	76,877216	83,586525	59,62726	65,31472	107,7949	100,2844	89,71943	86,48892	41,56886	29,37002	3,428541	2,575921	-0,86828	3,85E-01	1,00E+00	9109,5	10278,5	
CONTROL-KYNA	148	107	255	76,877216	65,253901	59,62726	55,10381	107,7949	83,45754	89,71943	73,40954	41,56886	27,5927	3,428541	2,68004	-3,26976	1,08E-03	1,62E-02	9819	6017	
CONTROL-LPS+KYNA	148	215	363	76,877216	79,27039	59,62726	64,21667	107,7949	101,3481	89,71943	86,91958	41,56886	32,54659	3,428541	2,224839	-0,74202	4,58E-01	1,00E+00	15180,5	16639,5	
CONTROL-SZR104	148	164	312	76,877216	116,67562	59,62726	90,88387	107,7949	164,184	89,71943	134,1879	41,56886	60,35826	3,428541	4,727623	-7,8066	5,87E-15	8,81E-14	5924	18348	
CONTROL-LPS+SZR104	148	209	357	76,877216	89,321277	59,62726	67,76135	107,7949	118,6333	89,71943	100,8863	41,56886	45,79642	3,428541	3,175411	-3,10214	1,92E-03	2,88E-02	12485,5	18446,5	
Circularity																					Kruskal-Wallis H statistic=74,32603925194775, p-value=1,2860102034675554e-14
SZR104-LPS+SZR104	164	209	373	0,472714	0,57479	0,35269	0,409766	0,600867	0,712989	0,47365	0,551494	0,174232	0,185485	0,013647	0,012861	-3,95861	7,54E-05	1,13E-03	13046	21230	
LPS+KYNA-SZR104	215	164	379	0,601394	0,472714	0,467334	0,35269	0,721002	0,600867	0,591441	0,47365	0,159867	0,174232	0,010928	0,013647	-6,20577	5,44E-10	8,16E-09	24188	11072	
LPS+KYNA-LPS+SZR104	215	209	424	0,601394	0,57479	0,467334	0,409766	0,721002	0,712989	0,591441	0,551494	0,159867	0,185485	0,010928	0,012861	-2,01343	4,41E-02	6,61E-01	25008	19927	
KYNA-LPS+KYNA	107	215	322	0,708405	0,601394	0,542238	0,467334	0,792865	0,721002	0,654411	0,591441	0,163423	0,159867	0,015873	0,010928	-3,49598	4,72E-04	7,09E-03	14254	8751	
KYNA-SZR104	107	164	271	0,708405	0,472714	0,542238	0,35269	0,792865	0,600867	0,654411	0,47365	0,163423	0,174232	0,015873	0,013647	-7,55616	4,15E-14	6,23E-13	13540	4008	
KYNA-LPS+SZR104	107	209	316	0,708405	0,57479	0,542238	0,409766	0,792865	0,712989	0,654411	0,551494	0,163423	0,185485	0,015873	0,012861	-4,70202	2,58E-06	3,86E-05	14796	7567	
LPS-KYNA	131	107	238	0,612114	0,708405	0,490102	0,542238	0,689722	0,792865	0,594926	0,654411	0,137639	0,163423	0,012072	0,015873	-3,39347	6,90E-04	1,04E-02	5215	8802	
LPS-LPS+KYNA	131	215	346	0,612114	0,601394	0,490102	0,467334	0,689722	0,721002	0,594926	0,591441	0,137639	0,159867	0,012072	0,010928	-0,041	9,67E-01	1,00E+00	14045	14120	
LPS-SZR104	131	164	295	0,612114	0,472714	0,490102	0,35269	0,689722	0,600867	0,594926	0,47365	0,137639	0,174232	0,012072	0,013647	-5,89654	3,71E-09	5,57E-08	15035	6449	
LPS-LPS+SZR104	131	209	340	0,612114	0,57479	0,490102	0,409766	0,689722	0,712989	0,594926	0,551494	0,137639	0,185485	0,012072	0,012861	-1,85816	6,31E-02	9,47E-01	15329	12050	
CONTROL-LPS	148	131	279	0,579778	0,612114	0,367577	0,490102	0,728114	0,689722	0,550972	0,594926	0,201349	0,137639	0,016607	0,012072	-1,2645	2,06E-01	1,00E+00	8843	10545	
CONTROL-KYNA	148	107	255	0,579778	0,708405	0,367577	0,542238	0,728114	0,792865	0,550972	0,654411	0,201349	0,163423	0,016607	0,015873	-4,15752	3,22E-05	4,83E-04	5501	10335	
CONTROL-LPS+KYNA	148	215	363	0,579778	0,601394	0,367577	0,467334	0,728114	0,721002	0,550972	0,591441	0,201349	0,159867	0,016607	0,010928	-1,48863	1,37E-01	1,00E+00	14447	17373	
CONTROL-SZR104	148	164	312	0,579778	0,472714	0,367577	0,35269	0,728114	0,600867	0,550972	0,47365	0,201349	0,174232	0,016607	0,013647	-3,65414	2,58E-04	3,87E-03	15044	9228	
CONTROL-LPS+SZR104	148	209	357	0,579778	0,57479	0,367577	0,409766	0,728114	0,712989	0,550972	0,551494	0,201349	0,185485	0,016607	0,012861	-0,10878	9,13E-01	1,00E+00	15571	15361	
TI																					Kruskal-Wallis H statistic=74,32603925194684, p-value=1,28601020346812e-14
SZR104-LPS+SZR104	164	209	373	2,115457	1,739765	1,664265	1,402546	2,835391	2,440418	2,538939	2,131877	1,298298	1,070127	0,101691	0,0742	-3,95861	7,54E-05	1,13E-03	21230	13046	
LPS+KYNA-SZR104	215	164	379	1,662803	2,115457	1,386959	1,664265	2,139797	2,835391	1,863021	2,538939	0,696934	1,298298	0,047641	0,101691	-6,20577	5,44E-10	8,16E-09	11072	24188	
LPS+KYNA-LPS+SZR104	215	209	424	1,662803	1,739765	1,386959	1,402546	2,139797	2,440418	1,863021	2,131877	0,696934	1,070127	0,047641	0,0742	-2,01343	4,41E-02	6,61E-01	19927	25008	
KYNA-LPS+KYNA	107	215	322	1,411622	1,662803	1,261255	1,386959	1,844245	2,139797	1,660913	1,863021	0,554219	0,696934	0,053831	0,047641	-3,49598	4,72E-04	7,09E-03	8751	14254	
KYNA-SZR104	107	164	271	1,411622	2,115457	1,261255	1,664265	1,844245	2,835391	1,660913	2,538939	0,554219	1,298298	0,053831	0,101691	-7,55616	4,15E-14	6,23E-13	4008	13540	
KYNA-LPS+SZR104	107	209	316	1,411622	1,739765	1,261255	1,402546	1,844245	2,440418	1,660913	2,131877	0,554219	1,070127	0,053831	0,0742	-4,70202	2,58E-06	3,86E-05	7567	14796	
LPS-KYNA	131	107	238	1,633683	1,411622	1,449866	1,261255	2,04042	1,844245	1,799395	1,660913	0,551593	0,554219	0,048378	0,053831	-3,39347	6,90E-04	1,04E-02	8802	5215	
LPS-LPS+KYNA	131	215	346	1,633683	1,662803	1,449866	1,386959	2,04042	2,139797	1,799395	1,863021	0,551593	0,696934	0,048378	0,047641	-0,041	9,67E-01	1,00E+00	14120	14045	
LPS-SZR104	131	164	295	1,633683	2,115457	1,449866	1,664265	2,04042	2,835391	1,799395	2,538939	0,551593	1,298298	0,048378	0,101691	-5,89654	3,71E-09	5,57E-08	6449	15035	
LPS-LPS+SZR104	131	209	340	1,633683	1,739765	1,449866	1,402546	2,04042	2,440418	1,799395	2,131877	0,551593	1,070127	0,048378	0,0742	-1,85816	6,31E-02	9,47E-01	12050	15329	
CONTROL-LPS	148	131	279	1,724816	1,633683	1,373426	1,449866	2,72052	2,04042	2,230989	1,799395	1,308515	0,551593	0,107924	0,048378	-1,2645	2,06E-01	1,00E+00	10545	8843	
CONTROL-KYNA	148	107	255	1,724816	1,411622	1,373426	1,261255	2,72052	1,844245	2,230989	1,660913	1,308515	0,554219	0,107924	0,053831	-4,15752	3,22E-05	4,83E-04	10335	5501	
CONTROL-LPS+KYNA	148	215	363	1,724816	1,662803	1,373426	1,386959	2,72052	2,139797	2,230989	1,863021	1,308515	0,696934	0,107924	0,047641	-1,48863	1,37E-01	1,00E+00	17373	14447	
CONTROL-SZR104	148	164	312	1,724816	2,115457	1,373426	1,664265	2,72052	2,835391	2,230989	2,538939	1,308515	1,298298	0,107924	0,101691	-3,65414	2,58E-04	3,87E-03	9228	15044	
CONTROL-LPS+SZR104	148	209	357	1,724816	1,739765	1,373426	1,402546	2,72052	2,440418	2,230989	2,131877	1,308515	1,070127	0,107924	0,0742	-0,10878	9,13E-01	1,00E+00	15361	15571	
Convex hull area																					Kruskal-Wallis H statistic=175,78900983519304, p-value=4,242269272588465e-36
SZR104-LPS+SZR104	164	209	373	581,91552	384,60088	397,9695	257,8781	904,5225	523,0496	710,7885	443,1239	445,7885	265,4401	34,91685	18,40496	-7,03774	1,95E-12	2,93E-11	24412,5	9863,5	
LPS+KYNA-SZR104	215	164	379	318,58307	581,91552	239,1259	397,9695	456,8589	904,5225	365,5611	710,7885	197,133	445,7885	13,47573	34,91685	-9,64628	5,10E-22	7,65E-21	7436,5	27823,5	
LPS+KYNA-LPS+SZR104	215	209	424	318,58307	384,60088	239,1259	257,8781	456,8589	523,0496	365,5611	443,1239	197,133	265,4401	13,47573	18,40496	-3,11409	1,85E-03	2,77E-02	18538,5	26396,5	
KYNA-LPS+KYNA	107	215	322	231,34525	318,58307	193,4793	239,1259	328,973	456,8589	290,9235	365,5611	186,1807	197,133	18,08347	13,47573	-4,81952	1,44E-06	2,16E-05	7709,5	15295,5	
KYNA-SZR104	107	164	271	231,34525	581,91552	193,4793	397,9695	328,973	904,5225	290,9235	710,7885	186,1807	445,7885	18,08347	34,91685	-10,4134	2,15E-25	3,23E-24	2206	15342	
KYNA-LPS+SZR104	107	209	316	231,34525	384,60088	193,4793	257,8781	328,973	523,0496	290,9235	443,1239	186,1807	265,4401	18,08347	18,40496	-6,85266	7,25E-12	1,09E-10	5914	16449	
LPS-KYNA	131	107	238	339,52015	231,34525	232,2412	193,4793	459,2953	328,973	373,6214	290,9235	191,3276	186,1807	16,78054	18,08347	-4,60475	4,13E-06	6,19E-05	9442	4575	
LPS-LPS+KYNA	131	215	346	339,52015	318,58307	232,2412	239,1259	459,2953	456,8589	373,6214	365,5611	191,3276	197,133	16,78054	13,47573	-0,47481	6,35E-01	1,00E+00	14511,5	13653,5	

LPS-SZR104	131	164	295	339,52015	581,91552	232,2412	397,9695	459,2953	904,5225	373,6214	710,7885	191,3276	445,7885	16,78054	34,91685	-8,22975	1,88E-16	2,81E-15	4750,5	16733,5	
LPS-LPS+SZR104	131	209	340	339,52015	384,60088	232,2412	257,8781	459,2953	523,0496	373,6214	443,1239	191,3276	265,4401	16,78054	18,40496	-2,37627	1,75E-02	2,62E-01	11593	15786	
CONTROL-LPS	148	131	279	288,16772	339,52015	199,2559	232,2412	448,7718	459,2953	356,0807	373,6214	219,567	191,3276	18,10958	16,78054	-1,89415	5,82E-02	8,73E-01	8419,5	10968,5	
CONTROL-KYNA	148	107	255	288,16772	231,34525	199,2559	193,4793	448,7718	328,973	356,0807	290,9235	219,567	186,1807	18,10958	18,08347	-2,46716	1,36E-02	2,04E-01	9352,5	6483,5	
CONTROL-LPS+KYNA	148	215	363	288,16772	318,58307	199,2559	239,1259	448,7718	456,8589	356,0807	365,5611	219,567	197,133	18,10958	13,47573	-1,67337	9,43E-02	1,00E+00	14265,5	17554,5	
CONTROL-SZR104	148	164	312	288,16772	581,91552	199,2559	397,9695	448,7718	904,5225	356,0807	710,7885	219,567	445,7885	18,10958	34,91685	-9,10488	8,64E-20	1,30E-18	4891	19381	
CONTROL-LPS+SZR104	148	209	357	288,16772	384,60088	199,2559	257,8781	448,7718	523,0496	356,0807	443,1239	219,567	265,4401	18,10958	18,40496	-4,07963	4,51E-05	6,77E-04	11546,5	19385,5	
Convex hull perimeter																					Kruskal-Wallis H statistic=162,49175417850043, p-value=2,9135527636475045e-33
SZR104-LPS+SZR104	164	209	373	89,813076	73,525461	76,04883	59,70067	115,5874	88,81071	97,73038	76,77258	31,87926	22,12158	2,496976	1,533856	-6,8631	6,74E-12	1,01E-10	24232	10044	
LPS+KYNA-SZR104	215	164	379	66,880603	89,813076	56,97566	76,04883	81,6496	115,5874	69,73218	97,73038	17,28979	31,87926	1,181906	2,496976	-9,5171	1,78E-21	2,67E-20	7573	27687	
LPS+KYNA-LPS+SZR104	215	209	424	66,880603	73,525461	56,97566	59,70067	81,6496	88,81071	69,73218	76,77258	17,28979	22,12158	1,181906	1,533856	-3,16997	1,52E-03	2,29E-02	18468	26467	
KYNA-LPS+KYNA	107	215	322	55,99656	66,880603	50,3602	56,97566	70,11787	81,6496	62,29892	69,73218	18,20522	17,28979	1,768247	1,181906	-4,54439	5,51E-06	8,26E-05	7926	15079	
KYNA-SZR104	107	164	271	55,99656	89,813076	50,3602	76,04883	70,11787	115,5874	62,29892	97,73038	18,20522	31,87926	1,768247	2,496976	-10,1248	4,29E-24	6,43E-23	2388	15160	
KYNA-LPS+SZR104	107	209	316	55,99656	73,525461	50,3602	59,70067	70,11787	88,81071	62,29892	76,77258	18,20522	22,12158	1,768247	1,533856	-6,61717	3,66E-11	5,49E-10	6095	16268	
LPS-KYNA	131	107	238	68,195213	55,99656	56,93755	50,3602	79,35671	70,11787	70,49491	62,29892	17,82474	18,20522	1,563333	1,768247	-4,37953	1,19E-05	1,78E-04	9323	4694	
LPS-LPS+KYNA	131	215	346	68,195213	66,880603	56,93755	56,97566	79,35671	81,6496	70,49491	69,73218	17,82474	17,28979	1,563333	1,181906	-0,29475	7,68E-01	1,00E+00	14349	13816	
LPS-SZR104	131	164	295	68,195213	89,813076	56,93755	76,04883	79,35671	115,5874	70,49491	97,73038	17,82474	31,87926	1,563333	2,496976	-8,29637	1,07E-16	1,61E-15	4702	16782	
LPS-LPS+SZR104	131	209	340	68,195213	73,525461	56,93755	59,70067	79,35671	88,81071	70,49491	76,77258	17,82474	22,12158	1,563333	1,533856	-2,53953	1,11E-02	1,67E-01	11449	15930	
CONTROL-LPS	148	131	279	64,02633	68,195213	53,69099	56,93755	83,53313	79,35671	70,71755	70,49491	23,30125	17,82474	1,921855	1,563333	-1,02067	3,07E-01	1,00E+00	9007	10381	
CONTROL-KYNA	148	107	255	64,02633	55,99656	53,69099	50,3602	83,53313	70,11787	70,71755	62,29892	23,30125	18,20522	1,921855	1,768247	-3,00825	2,63E-03	3,94E-02	9667	6169	
CONTROL-LPS+KYNA	148	215	363	64,02633	66,880603	53,69099	56,97566	83,53313	81,6496	70,71755	69,73218	23,30125	17,28979	1,921855	1,181906	-0,76085	4,47E-01	1,00E+00	15162	16658	
CONTROL-SZR104	148	164	312	64,02633	89,813076	53,69099	76,04883	83,53313	115,5874	70,71755	97,73038	23,30125	31,87926	1,921855	2,496976	-8,21381	2,14E-16	3,21E-15	5600	18672	
CONTROL-LPS+SZR104	148	209	357	64,02633	73,525461	53,69099	59,70067	83,53313	88,81071	70,71755	76,77258	23,30125	22,12158	1,921855	1,533856	-3,18386	1,45E-03	2,18E-02	12407	18525	
Mean radius of the convex hull																					Kruskal-Wallis H statistic=146,6833156143014, p-value=6,782100501488875e-30
SZR104-LPS+SZR104	164	209	373	15,352207	12,482411	12,67488	10,07606	19,93019	14,91507	16,74358	13,10396	5,826713	3,919799	0,456383	0,271789	-6,62025	3,59E-11	5,38E-10	23981	10295	
LPS+KYNA-SZR104	215	164	379	11,305709	15,352207	9,769672	12,67488	13,77025	19,93019	11,86537	16,74358	2,932915	5,826713	0,20049	0,456383	-9,27199	1,83E-20	2,74E-19	7832	27428	
LPS+KYNA-LPS+SZR104	215	209	424	11,305709	12,482411	9,769672	10,07606	13,77025	14,91507	11,86537	13,10396	2,932915	3,919799	0,20049	0,271789	-3,09863	1,94E-03	2,92E-02	18558	26377	
KYNA-LPS+KYNA	107	215	322	9,754521	11,305709	8,787261	9,769672	12,20578	13,77025	10,76799	11,86537	3,265157	2,932915	0,31714	0,20049	-4,11486	3,87E-05	5,81E-04	8264	14741	
KYNA-SZR104	107	164	271	9,754521	15,352207	8,787261	12,67488	12,20578	19,93019	10,76799	16,74358	3,265157	5,826713	0,31714	0,456383	-9,74111	2,01E-22	3,02E-21	2630	14918	
KYNA-LPS+SZR104	107	209	316	9,754521	12,482411	8,787261	10,07606	12,20578	14,91507	10,76799	13,10396	3,265157	3,919799	0,31714	0,271789	-6,07853	1,21E-09	1,82E-08	6509	15854	
LPS-KYNA	131	107	238	11,410035	9,754521	9,541924	8,787261	13,60335	12,20578	11,98624	10,76799	3,152782	3,265157	0,276517	0,31714	-3,76064	1,69E-04	2,54E-03	8996	5021	
LPS-LPS+KYNA	131	215	346	11,410035	11,305709	9,541924	9,769672	13,60335	13,77025	11,98624	11,86537	3,152782	2,932915	0,276517	0,20049	-0,14738	8,83E-01	1,00E+00	14216	13949	
LPS-SZR104	131	164	295	11,410035	15,352207	9,541924	12,67488	13,60335	19,93019	11,98624	16,74358	3,152782	5,826713	0,276517	0,456383	-8,13016	4,29E-16	6,43E-15	4823	16661	
LPS-LPS+SZR104	131	209	340	11,410035	12,482411	9,541924	10,07606	13,60335	14,91507	11,98624	13,10396	3,152782	3,919799	0,276517	0,271789	-2,5452	1,09E-02	1,64E-01	11444	15935	
CONTROL-LPS	148	131	279	11,040789	11,410035	9,265137	9,541924	14,3615	13,60335	12,36874	11,98624	4,277379	3,152782	0,352792	0,276517	-0,19997	8,42E-01	1,00E+00	9559	9829	
CONTROL-KYNA	148	107	255	11,040789	9,754521	9,265137	8,787261	14,3615	12,20578	12,36874	10,76799	4,277379	3,265157	0,352792	0,31714	-3,20954	1,33E-03	1,99E-02	9784	6052	
CONTROL-LPS+KYNA	148	215	363	11,040789	11,305709	9,265137	9,769672	14,3615	13,77025	12,36874	11,86537	4,277379	2,932915	0,352792	0,20049	-0,1023	9,19E-01	1,00E+00	15809	16011	
CONTROL-SZR104	148	164	312	11,040789	15,352207	9,265137	12,67488	14,3615	19,93019	12,36874	16,74358	4,277379	5,826713	0,352792	0,456383	-7,51377	5,74E-14	8,62E-13	6157	18115	
CONTROL-LPS+SZR104	148	209	357	11,040789	12,482411	9,265137	10,07606	14,3615	14,91507	12,36874	13,10396	4,277379	3,919799	0,352792	0,271789	-2,45517	1,41E-02	2,11E-01	13107	17825	
Maximum span across the convex hull																					Kruskal-Wallis H statistic=142,1787357539573, p-value=6,158594797292046e-29
SZR104-LPS+SZR104	164	209	373	33,193848	27,22945	27,74474	21,55988	45,07949	33,23746	36,56048	28,37468	13,4454	9,158768	1,053125	0,635046	-6,48141	9,09E-11	1,36E-09	23837,5	10438,5	
LPS+KYNA-SZR104	215	164	379	24,88594	33,193848	20,33796	27,74474	29,46746	45,07949	25,64416	36,56048	7,053306	13,4454	0,482154	1,053125	-9,03493	1,64E-19	2,46E-18	8082,5	27177,5	
LPS+KYNA-LPS+SZR104	215	209	424	24,88594	27,22945	20,33796	21,55988	29,46746	33,23746	25,64416	28,37468	7,053306	9,158768	0,482154	0,635046	-2,98805	2,81E-03	4,21E-02	18697,5	26237,5	
KYNA-LPS+KYNA	107	215	322	20,711312	24,88594	17,49262	20,33796	26,39787	29,46746	22,98943	25,64416	8,029291	7,053306	0,779874	0,482154	-4,2591	2,05E-05	3,08E-04	8150,5	14854,5	
KYNA-SZR104	107	164	271	20,711312	33,193848	17,49262	27,74474	26,39787	45,07949	22,98943	36,56048	8,029291	13,4454	0,779874	1,053125	-9,50168	2,07E-21	3,10E-20	2781	14767	
KYNA-LPS+SZR104	107	209	316	20,711312	27,22945	17,49262	21,55988	26,39787	33,23746	22,98943	28,37468	8,029291	9,158768	0,779874	0,635046	-6,07788	1,22E-09	1,83E-08	6509,5	15853,5	
LPS-KYNA	131	107	238	24,695727	20,711312	20,31738	17,49262	29,04574	26,39787	25,60593	22,98943	7,277575	8,029291	0,638286	0,779874	-3,78998	1,51E-04	2,26E-03	9011,5	5005,5	
LPS-LPS+KYNA	131	215	346	24,695727	24,88594	20,31738	20,33796	29,04574	29,46746	25,60593	25,64416	7,277575	7,053306	0,638286	0,482154	-0,19059	8,49E-01	1,00E+00	13910	14255	
LPS-SZR104	131	164	295	24,695727	33,193848	20,31738	27,74474	29,04574	45,07949	25,60593	36,56048	7,277575	13,4454	0,638286	1,053125	-8,14939	3,66E-16	5,49E-15	4809	16675	
LPS-LPS+SZR104	131	209	340	24,695727	27,22945	20,31738	21,55988	29,04574	33,23746	25,60593	28,37468	7,277575	9,158768	0,638286	0,635046	-2,87681	4,02E-03	6,03E-02	11151,5	16227,5	
CONTROL-LPS	148	131	279	23,779043	24,695727	19,88169	20,31738	30,82951	29,04574	27,03559	25,60593	10,47948	7,277575	0,864333	0,638286	-0,08103	9,35E-01	1,00E+00	9749	9639	
CONTROL-KYNA	148	107	255	23,779043	20,711312	19,88169	17,49262	30,82951	26,39787	27,03559	22,98943	10,47948	8,029291	0,864333	0,779874	-3,47966	5,02E-04	7,53E-03	9941	5895	
CONTROL-LPS+KYNA	148	215	363	23,779043	24,88594	19,88169	20,33796	30,82951	29,46746	27,03559	25,64416	10,47948	7,053306	0,864333	0,482154	-0,11044	9,12E-01	1,00E+00	16019	15801	
CONTROL-SZR104	148	164	312	23,779043	33,193848	19,88169	27,74474	30,82951	45,07949	27,03559	36,56048	10,47948	13,4454	0,864333	1,053125	-7,17318	7,33E-13	1,10E-11	6428	17844	
CONTROL-LPS+SZR104	148	209	357	23,779043	27,22945	19,88169	21,55988	30,82951	33,23746	27,03559	28,37468	10,47948	9,158768	0,864333	0,635046	-2,20897	2,72E-02	4,08E-01	13343,5	17588,5	
Diameter of the bounding circle																					Kruskal-Wallis H statistic=142,68617585179265, p-value=4,803755926029199e-29
SZR104-LPS+SZR104	164	209	373	33,497544	27,253103	27,96823	21,58495	45,22266	33,23746	36,7487	28,53666	13,44484	9,163669	1,053081	0,635386	-6,48528	8,86E-11	1,33E-09	23841,5	10434,5	
LPS+KYNA-SZR104	215	164	379	25,040355	33,497544	20,39372	27,96823	29,62975	45,22266	25,78413	36,7487	7,071032	13,44484	0,483366	1,053081	-9,05007	1,43E-19	2,14E-18	8066,5	27193,5	
LPS+KYNA-LPS+SZR104	215	209	424	25,040355	27,253103	20,39372	21,58495	29,62975	33,23746	25,78413	28,53666	7,071032	9,163669	0,483366	0,635386	-2,98805	2,81E-03	4,21E-02	18697,5	26237,5	
KYNA-LPS+KYNA	107	215	322	20,711312	25,040355	17,54542	20,39372	26,49492	29,62975	23,08906	25,78413	8,016708	7,071032	0,778652	0,483366	-4,26418	2,01E-05	3,01E-04	8146,5	14858,5	
KYNA-SZR104	107	164	271	20,711312	33,497544	17,54542	27,96823	26,49492	45,22266	23,08906	36,7487	8,016708	13,44484	0,778652	1,053081	-9,53656	1,48E-21	2,22E-20	2759	14789	
KYNA-LPS+SZR104	107	209	316	20,711312	27,253103	17,54542	21,58495	26,49492	33,23746	23,08906	28,53666	8,016708	9,163669	0,778652	0,635386	-6,12212	9,23E-10	1,39E-08	6475,5	15887,5	
LPS-KYNA	131	107	238	24,763706	20,711312	20,39887	17,54542	29,08973	26,49492	25,78682	23,08906	7,326205	8,016708	0,642551	0,778652	-3,84203	1,22E-04	1,83E-03	9039	4978	
LPS-LPS+KYNA	131	215	346	24,763706	25,040355	20,39887	20,39372	29,08973	29,62975	25,78682	25,78413	7,326205	7,071032	0,642551	0,483366	-0,10693	9,15E-01	1,00E+00	13985,5	14179,5	
LPS-SZR104	131	164	295	24,763706	33,497544	20,39887	27,96823	29,08973	45,22266	25,78682	36,7487	7,326205	13,44484	0,642551	1,053081	-8,14664	3,74E-16	5,61E-15	4811	16673	
LPS-LPS+SZR104	131	209	340	24,763706	27,253103	20,39887	21,58495	29,08973	33,23746	25,78682	28,53666	7,326205	9,163669	0,642551	0,635386	-2,85357	4,32E-03	6,49E-02	11172	16207	
CONTROL-LPS	148	131	279	23,787668	24,763706	19,94684	20,39887	31,48334	29,08973	27,18792	25,78682	10,47205	7,326205	0,86372	0,642551	-0,09887	9,21E-01	1,00E+00	9761	9627	
CONTROL-KYNA	148	107	255	23,787668	20,711312	19,94684	17,54542	31,48334	26,49492	27,18792	23,08906	10,47205	8,016708	0,86372	0,778652	-3,52009	4,31E-04	6,47E-03	9964,5	5871,5	
CONTROL-LPS+KYNA	148	215	363	23,787668	25,040355	19,94684	20,39372	31,48334	29,62975	27,18792	25,78413	10,47205	7,071032	0,86372	0,483366	-0,15166	8,79E-01	1,00E+00	16059,5	15760,5	
CONTROL-SZR104	148	164	312	23,787668	33,497544	19,94684	27,96823	31,48334	45,22266	27,18792	36,7487	10,47205	13,44484	0,86372	1,053081	-7,15307	8,49E-13	1,27E-11	6444	17828	
CONTROL-LPS+SZR104	148	209	357	23,787668	27,253103	19,94684	21,58495	31,48334	33,23746	27,18792	28,53666	10,47205	9,163669	0,86372	0,635386	-2,19908	2,79E-02	4,18E-01	13353	17579	
The ratio maximum/minimum convex hull radii																					Kruskal-Wallis H statistic=27,830879453912114, p-value=3,927678321352313e-05
SZR104-LPS+SZR104	164	209	373	2,0562	2,2262	1,67945	1,7655	2,517975	2,8529	2,186927	2,480052	0,655597	1,110152	0,05135	0,076975	-2,08017	0,03751	5,63E-01	14987,5	19288,5	
LPS+KYNA-SZR104	215	164	379	2,1908	2,0562	1,76575	1,67945	2,78585	2,517975	2,439013	2,186927	0,947196	0,655597	0,064749	0,05135	-2,31764	0,020469	3,07E-01	20079,5	15180,5	

LPS+KYNA-LPS+SZR104	215	209	424	2,1908	2,2262	1,76575	1,7655	2,78585	2,8529	2,439013	2,480052	0,947196	1,110152	0,064749	0,076975	-0,02061	0,983557	1,00E+00	22494	22441	
KYNA-LPS+KYNA	107	215	322	2,5775	2,1908	1,8488	1,76575	3,177	2,78585	2,708796	2,439013	1,189976	0,947196	0,115581	0,064749	-2,04917	0,040445	6,07E-01	13115,5	9889,5	
KYNA-SZR104	107	164	271	2,5775	2,0562	1,8488	1,67945	3,177	2,517975	2,708796	2,186927	1,189976	0,655597	0,115581	0,05135	-3,69999	0,000216	3,24E-03	11108	6440	
KYNA-LPS+SZR104	107	209	316	2,5775	2,2262	1,8488	1,7655	3,177	2,8529	2,708796	2,480052	1,189976	1,110152	0,115581	0,076975	-1,91906	0,054977	8,25E-01	12657	9706	
LPS-KYNA	131	107	238	2,0721	2,5775	1,682	1,8488	2,6434	3,177	2,312964	2,708796	0,969367	1,189976	0,085019	0,115581	-3,03671	0,002392	3,59E-02	5403,5	8613,5	
LPS-LPS+KYNA	131	215	346	2,0721	2,1908	1,682	1,76575	2,6434	2,78585	2,312964	2,439013	0,969367	0,947196	0,085019	0,064749	-1,62001	0,105229	1,00E+00	12620	15545	
LPS-SZR104	131	164	295	2,0721	2,0562	1,682	1,67945	2,6434	2,517975	2,312964	2,186927	0,969367	0,655597	0,085019	0,05135	-0,3757	0,707138	1,00E+00	11016	10468	
LPS-LPS+SZR104	131	209	340	2,0721	2,2262	1,682	1,7655	2,6434	2,8529	2,312964	2,480052	0,969367	1,110152	0,085019	0,076975	-1,48857	0,1366	1,00E+00	12376	15003	
CONTROL-LPS	148	131	279	2,43455	2,0721	1,9289	1,682	2,998075	2,6434	2,599627	2,312964	0,914128	0,969367	0,075396	0,085019	-3,54373	0,000395	5,93E-03	12078	7310	
CONTROL-KYNA	148	107	255	2,43455	2,5775	1,9289	1,8488	2,998075	3,177	2,599627	2,708796	0,914128	1,189976	0,075396	0,115581	-0,15398	0,877624	1,00E+00	7828	8008	
CONTROL-LPS+KYNA	148	215	363	2,43455	2,1908	1,9289	1,76575	2,998075	2,78585	2,599627	2,439013	0,914128	0,947196	0,075396	0,064749	-2,34923	0,018812	2,82E-01	18218,5	13601,5	
CONTROL-SZR104	148	164	312	2,43455	2,0562	1,9289	1,67945	2,998075	2,517975	2,599627	2,186927	0,914128	0,655597	0,075396	0,05135	-4,34664	0,000014	2,10E-04	15595	8677	
CONTROL-LPS+SZR104	148	209	357	2,43455	2,2262	1,9289	1,7655	2,998075	2,8529	2,599627	2,480052	0,914128	1,110152	0,075396	0,076975	-2,30318	0,021269	3,19E-01	17679	13253	
Convex hull circularity																					Kruskal-Wallis H statistic=35,915502735983736, p-value=9,87500430584392e-07
SZR104-LPS+SZR104	164	209	373	0,902669	0,918298	0,845733	0,847277	0,937594	0,947842	0,874552	0,889598	0,087759	0,077608	0,006874	0,005381	-1,92295	0,054487	8,17E-01	15150	19126	
LPS+KYNA-SZR104	215	164	379	0,914743	0,902669	0,864835	0,845733	0,949328	0,937594	0,900945	0,874552	0,06726	0,087759	0,004598	0,006874	-2,97868	0,002895	4,34E-02	20778	14482	
LPS+KYNA-LPS+SZR104	215	209	424	0,914743	0,918298	0,864835	0,847277	0,949328	0,947842	0,900945	0,889598	0,06726	0,077608	0,004598	0,005381	-1,10263	0,270187	1,00E+00	23859	21076	
KYNA-LPS+KYNA	107	215	322	0,936686	0,914743	0,874595	0,864835	0,960115	0,949328	0,902639	0,900945	0,084544	0,06726	0,008212	0,004598	-1,59232	0,111314	1,00E+00	12756	10249	
KYNA-SZR104	107	164	271	0,936686	0,902669	0,874595	0,845733	0,960115	0,937594	0,902639	0,874552	0,084544	0,087759	0,008212	0,006874	-4,03455	0,000055	8,25E-04	11319	6229	
KYNA-LPS+SZR104	107	209	316	0,936686	0,918298	0,874595	0,847277	0,960115	0,947842	0,902639	0,889598	0,084544	0,077608	0,008212	0,005381	-2,41216	0,015858	2,38E-01	13036	9327	
LPS-KYNA	131	107	238	0,921307	0,936686	0,879463	0,874595	0,949242	0,960115	0,900798	0,902639	0,072915	0,084544	0,006395	0,008212	-1,55006	0,121128	1,00E+00	6189	7828	
LPS-LPS+KYNA	131	215	346	0,921307	0,914743	0,879463	0,864835	0,949242	0,949328	0,900798	0,900945	0,072915	0,06726	0,006395	0,004598	-0,40334	0,686697	1,00E+00	14447	13718	
LPS-SZR104	131	164	295	0,921307	0,902669	0,879463	0,845733	0,949242	0,937594	0,900798	0,874552	0,072915	0,087759	0,006395	0,006874	-3,05439	0,002255	3,38E-02	12966	8518	
LPS-LPS+SZR104	131	209	340	0,921307	0,918298	0,879463	0,847277	0,949242	0,947842	0,900798	0,889598	0,072915	0,077608	0,006395	0,005381	-1,15639	0,247521	1,00E+00	14710	12669	
CONTROL-LPS	148	131	279	0,884521	0,921307	0,81198	0,879463	0,937839	0,949242	0,85356	0,900798	0,107286	0,072915	0,008849	0,006395	-3,8277	0,000129	1,94E-03	7119	12269	
CONTROL-KYNA	148	107	255	0,884521	0,936686	0,81198	0,874595	0,937839	0,960115	0,85356	0,902639	0,107286	0,084544	0,008849	0,008212	-4,40527	0,000011	1,65E-04	5357	10479	
CONTROL-LPS+KYNA	148	215	363	0,884521	0,914743	0,81198	0,864835	0,937839	0,949328	0,85356	0,900945	0,107286	0,06726	0,008849	0,004598	-4,06077	0,000049	7,35E-04	11920	19900	
CONTROL-SZR104	148	164	312	0,884521	0,902669	0,81198	0,845733	0,937839	0,937594	0,85356	0,874552	0,107286	0,087759	0,008849	0,006874	-1,34792	0,177685	1,00E+00	11063	13209	
CONTROL-LPS+SZR104	148	209	357	0,884521	0,918298	0,81198	0,847277	0,937839	0,947842	0,85356	0,889598	0,107286	0,077608	0,008849	0,005381	-3,02355	0,002498	3,75E-02	12561	18371	
Span ratio of the convex hull																					Kruskal-Wallis H statistic=18,711094609125098, p-value=0,002175377626330435
SZR104-LPS+SZR104	164	209	373	1,30035	1,2901	1,187475	1,1679	1,589325	1,5479	1,470377	1,430428	0,459771	0,380788	0,036012	0,026403	-0,77547	0,438064	1,00E+00	17940	16336	
LPS+KYNA-SZR104	215	164	379	1,3201	1,30035	1,16415	1,187475	1,5434	1,589325	1,39912	1,470377	0,342365	0,459771	0,023404	0,036012	-0,95725	0,338443	1,00E+00	16618	18642	
LPS+KYNA-LPS+SZR104	215	209	424	1,3201	1,2901	1,16415	1,1679	1,5434	1,5479	1,39912	1,430428	0,342365	0,380788	0,023404	0,026403	-0,27665	0,782049	1,00E+00	22118	22817	
KYNA-LPS+KYNA	107	215	322	1,24	1,3201	1,14235	1,16415	1,4277	1,5434	1,3791	1,39912	0,414893	0,342365	0,040298	0,023404	-1,98881	0,046722	7,01E-01	9937	13068	
KYNA-SZR104	107	164	271	1,24	1,30035	1,14235	1,187475	1,4277	1,589325	1,3791	1,470377	0,414893	0,459771	0,040298	0,036012	-2,64874	0,008079	1,21E-01	7103	10445	
KYNA-LPS+SZR104	107	209	316	1,24	1,2901	1,14235	1,1679	1,4277	1,5479	1,3791	1,430428	0,414893	0,380788	0,040298	0,026403	-1,99972	0,04553	6,83E-01	9644	12719	
LPS-KYNA	131	107	238	1,2535	1,24	1,143	1,14235	1,40775	1,4277	1,367149	1,3791	0,374569	0,414893	0,032852	0,040298	-0,41165	0,680599	1,00E+00	7226,5	6790,5	
LPS-LPS+KYNA	131	215	346	1,2535	1,3201	1,143	1,16415	1,40775	1,5434	1,367149	1,39912	0,374569	0,342365	0,032852	0,023404	-1,73027	0,083582	1,00E+00	12520,5	15644,5	
LPS-SZR104	131	164	295	1,2535	1,30035	1,143	1,187475	1,40775	1,589325	1,367149	1,470377	0,374569	0,459771	0,032852	0,036012	-2,35793	0,018377	2,76E-01	9025	12459	
LPS-LPS+SZR104	131	209	340	1,2535	1,2901	1,143	1,1679	1,40775	1,5479	1,367149	1,430428	0,374569	0,380788	0,032852	0,026403	-1,81338	0,069773	1,00E+00	12089,5	15289,5	
CONTROL-LPS	148	131	279	1,40735	1,2535	1,1601	1,143	1,685375	1,40775	1,547539	1,367149	0,508331	0,374569	0,041926	0,032852	-3,38464	0,000713	1,07E-02	11971	7417	
CONTROL-KYNA	148	107	255	1,40735	1,24	1,1601	1,14235	1,685375	1,4277	1,547539	1,3791	0,508331	0,414893	0,041926	0,040298	-3,2147	0,001306	1,96E-02	9787	6049	
CONTROL-LPS+KYNA	148	215	363	1,40735	1,3201	1,1601	1,16415	1,685375	1,5434	1,547539	1,39912	0,508331	0,342365	0,041926	0,023404	-2,21691	0,026629	3,99E-01	18088,5	13731,5	
CONTROL-SZR104	148	164	312	1,40735	1,30035	1,1601	1,187475	1,685375	1,589325	1,547539	1,470377	0,508331	0,459771	0,041926	0,036012	-1,21784	0,223285	1,00E+00	13105,5	11166,5	
CONTROL-LPS+SZR104	148	209	357	1,40735	1,2901	1,1601	1,1679	1,685375	1,5479	1,547539	1,430428	0,508331	0,380788	0,041926	0,026403	-1,88054	0,060034	9,01E-01	17273	13659	
Density																					Kruskal-Wallis H statistic=68,9878401100331, p-value=1,6641930555954293e-13
SZR104-LPS+SZR104	164	209	373	0,90147	0,928966	0,856215	0,890568	0,940043	0,967588	0,878695	0,918235	0,094619	0,064602	0,007411	0,004479	-4,93096	8,18E-07	1,23E-05	12041	22235	
LPS+KYNA-SZR104	215	164	379	0,939061	0,90147	0,908073	0,856215	0,971077	0,940043	0,929989	0,878695	0,053334	0,094619	0,003646	0,007411	-6,62217	3,54E-11	5,31E-10	24628	10632	
LPS+KYNA-LPS+SZR104	215	209	424	0,939061	0,928966	0,908073	0,890568	0,971077	0,967588	0,929989	0,918235	0,053334	0,064602	0,003646	0,004479	-1,71142	8,70E-02	1,00E+00	24627	20308	
KYNA-LPS+KYNA	107	215	322	0,958879	0,939061	0,908425	0,908073	0,981775	0,971077	0,938015	0,929989	0,060643	0,053334	0,00589	0,003646	-2,2722	2,31E-02	3,46E-01	13291	9714	
KYNA-SZR104	107	164	271	0,958879	0,90147	0,908425	0,856215	0,981775	0,940043	0,938015	0,878695	0,060643	0,094619	0,00589	0,007411	-6,71896	1,83E-11	2,75E-10	13012	4536	
KYNA-LPS+SZR104	107	209	316	0,958879	0,928966	0,908425	0,890568	0,981775	0,967588	0,938015	0,918235	0,060643	0,064602	0,00589	0,004479	-3,46211	5,36E-04	8,04E-03	13843	8520	
LPS-KYNA	131	107	238	0,944896	0,958879	0,913345	0,908425	0,964234	0,981775	0,931144	0,938015	0,05372	0,060643	0,004712	0,00589	-2,26926	2,33E-02	3,49E-01	5809	8208	
LPS-LPS+KYNA	131	215	346	0,944896	0,939061	0,913345	0,908073	0,964234	0,971077	0,931144	0,929989	0,05372	0,053334	0,004712	0,003646	-0,08975	9,28E-01	1,00E+00	14164	14001	
LPS-SZR104	131	164	295	0,944896	0,90147	0,913345	0,856215	0,964234	0,940043	0,931144	0,878695	0,05372	0,094619	0,004712	0,007411	-6,13007	8,78E-10	1,32E-08	15205	6279	
LPS-LPS+SZR104	131	209	340	0,944896	0,928966	0,913345	0,890568	0,964234	0,967588	0,931144	0,918235	0,05372	0,064602	0,004712	0,004479	-1,54186	1,23E-01	1,00E+00	15050	12329	
CONTROL-LPS	148	131	279	0,933456	0,944896	0,846785	0,913345	0,969016	0,964234	0,892557	0,931144	0,103637	0,05372	0,008548	0,004712	-1,97072	4,88E-02	7,31E-01	8368	11020	
CONTROL-KYNA	148	107	255	0,933456	0,958879	0,846785	0,908425	0,969016	0,981775	0,892557	0,938015	0,103637	0,060643	0,008548	0,00589	-3,79794	1,46E-04	2,19E-03	5710	10126	
CONTROL-LPS+KYNA	148	215	363	0,933456	0,939061	0,846785	0,908073	0,969016	0,971077	0,892557	0,929989	0,103637	0,053334	0,008548	0,003646	-2,3019	2,13E-02	3,20E-01	13648	18172	
CONTROL-SZR104	148	164	312	0,933456	0,90147	0,846785	0,856215	0,969016	0,940043	0,892557	0,878695	0,103637	0,094619	0,008548	0,007411	-2,86236	4,20E-03	6,31E-02	14414	9858	
CONTROL-LPS+SZR104	148	209	357	0,933456	0,928966	0,846785	0,890568	0,969016	0,967588	0,892557	0,918235	0,103637	0,064602	0,008548	0,004479	-1,14248	2,53E-01	1,00E+00	14368	16564	
Roughness																					Kruskal-Wallis H statistic=83,66798054694024, p-value=1,431738025405068e-16
SZR104-LPS+SZR104	164	209	373	1,27068	1,189725	1,186345	1,109959	1,434234	1,39061	1,334733	1,27302	0,220447	0,222469	0,017267	0,015425	-3,95957	7,51E-05	1,13E-03	21231	13045	
LPS+KYNA-SZR104	215	164	379	1,163027	1,27068	1,113219	1,186345	1,282123	1,434234	1,222701	1,334733	0,161914	0,220447	0,011068	0,017267	-6,01272	1,82E-09	2,74E-08	11276	23984	
LPS+KYNA-LPS+SZR104	215	209	424	1,163027	1,189725	1,113219	1,109959	1,282123	1,39061	1,222701	1,27302	0,161914	0,222469	0,011068	0,015425	-1,53703	1,24E-01	1,00E+00	20528	24407	
KYNA-LPS+KYNA	107	215	322	1,118405	1,163027	1,081688	1,113219	1,194705	1,282123	1,15977	1,222701	0,111648	0,161914	0,010844	0,011068	-4,38681	1,15E-05	1,73E-04	8050	14955	
KYNA-SZR104	107	164	271	1,118405	1,27068	1,081688	1,186345	1,194705	1,434234	1,15977	1,334733	0,111648	0,220447	0,010844	0,017267	-8,39335	4,72E-17	7,09E-16	3480	14068	
KYNA-LPS+SZR104	107	209	316	1,118405	1,189725	1,081688	1,109959	1,194705	1,39061	1,15977	1,27302	0,111648	0,222469	0,010844	0,015425	-5,14307	2,70E-07	4,05E-06	7228	15135	
LPS-KYNA	131	107	238	1,175531	1,118405	1,119892	1,081688	1,259101	1,194705	1,208841	1,15977	0,130697	0,111648	0,011463	0,010844	-4,06914	4,72E-05	7,08E-04	9159	4858	
LPS-LPS+KYNA	131	215	346	1,175531	1,163027	1,119892	1,113219	1,259101	1,282123	1,208841	1,222701	0,130697	0,161914	0,011463	0,011068	-0,01995	9,84E-01	1,00E+00	14101	14064	
LPS-SZR104	131	164	295	1,175531	1,27068	1,119892	1,186345	1,259101	1,434234	1,208841	1,334733	0,130697	0,220447	0,011463	0,017267	-5,59296	2,23E-08	3,35E-07	6670	14814	
LPS-LPS+SZR104	131	209	340	1,175531	1,189725	1,119892	1,109959	1,259101	1,39061	1,208841	1,27302	0,130697	0,222469	0,011463	0,015425	-1,3888	1,65E-01	1,00E+00	12464	14915	
CONTROL-LPS	148	131	279	1,157228	1,175531	1,09821	1,119892	1,333607	1,259101	1,229795	1,208841	0,17752	0,130697	0,014642	0,011463	-0,6802	4,96E-01	1,00E+00	9236	10152	
CONTROL-KYNA	148	107	255	1,157228	1,118405	1,09821	1,081688	1,333607	1,194705	1,229795	1,15977	0,17752	0,111648	0,014642	0,010844	-3,04782	2,31E-03	3,46E-02	9690	6146	
CONTROL-LPS+KYNA	148	215	363	1,157228	1,163027	1,09821	1,113219	1,333607	1,282123	1,229795	1,222701	0,17752	0,161914	0,014642	0,011068	-0,74559	4,56E-01	1,00E+00	15177	16643	
CONTROL-SZR104	148	164	312	1,157228	1,27068	1,09821	1,186345	1,333607	1,434234	1,229795	1,334733	0,17752	0,220447	0,014642	0,017267	-5,49284	3,96E-08	5,93E-07	7765	16507	

CONTROL-LPS+SZR104	148	209	357	1,157228	1,189725	1,09821	1,109959	1,333607	1,39061	1,229795	1,27302	0,17752	0,222469	0,014642	0,015425	-2,01275	4,41E-02	6,62E-01	13532	17400

Fractal dimension

Kruskal-Wallis H statistic=61,76089993261856, p-value=5,255330840669825e-12

SZR104-LPS+SZR104	164	209	373	1,0767	1,067	1,05765	1,0423	1,106425	1,1073	1,087849	1,077493	0,04026	0,044046	0,003153	0,003054	-3,16282	1,56E-03	2,34E-02	20407,5	13868,5
LPS+KYNA-SZR104	215	164	379	1,0616	1,0767	1,04405	1,05765	1,0879	1,106425	1,069682	1,087849	0,034995	0,04026	0,002392	0,003153	-4,87708	1,08E-06	1,62E-05	12476	22784
LPS+KYNA-LPS+SZR104	215	209	424	1,0616	1,067	1,04405	1,0423	1,0879	1,1073	1,069682	1,077493	0,034995	0,044046	0,002392	0,003054	-1,21401	2,25E-01	1,00E+00	20935,5	23999,5
KYNA-LPS+KYNA	107	215	322	1,0493	1,0616	1,03685	1,04405	1,0647	1,0879	1,054827	1,069682	0,028332	0,034995	0,002752	0,002392	-3,81114	1,38E-04	2,08E-03	8503	14502
KYNA-SZR104	107	164	271	1,0493	1,0767	1,03685	1,05765	1,0647	1,106425	1,054827	1,087849	0,028332	0,04026	0,002752	0,003153	-7,36033	1,83E-13	2,75E-12	4131,5	13416,5
KYNA-LPS+SZR104	107	209	316	1,0493	1,067	1,03685	1,0423	1,0647	1,1073	1,054827	1,077493	0,028332	0,044046	0,002752	0,003054	-4,31886	1,57E-05	2,35E-04	7861,5	14501,5
LPS-KYNA	131	107	238	1,0611	1,0493	1,04425	1,03685	1,08015	1,0647	1,064819	1,054827	0,028907	0,028332	0,002535	0,002752	-3,04334	2,34E-03	3,51E-02	8617	5400
LPS-LPS+KYNA	131	215	346	1,0611	1,0616	1,04425	1,04405	1,08015	1,0879	1,064819	1,069682	0,028907	0,034995	0,002535	0,002392	-0,75682	4,49E-01	1,00E+00	13399	14766
LPS-SZR104	131	164	295	1,0611	1,0767	1,04425	1,05765	1,08015	1,106425	1,064819	1,087849	0,028907	0,04026	0,002535	0,003153	-5,22206	1,77E-07	2,65E-06	6940	14544
LPS-LPS+SZR104	131	209	340	1,0611	1,067	1,04425	1,0423	1,08015	1,1073	1,064819	1,077493	0,028907	0,044046	0,002535	0,003054	-1,82528	6,80E-02	1,00E+00	12079	15300
CONTROL-LPS	148	131	279	1,05605	1,0611	1,039975	1,04425	1,091425	1,08015	1,069746	1,064819	0,040748	0,028907	0,003361	0,002535	-0,12415	9,01E-01	1,00E+00	9610	9778
CONTROL-KYNA	148	107	255	1,05605	1,0493	1,039975	1,03685	1,091425	1,0647	1,069746	1,054827	0,040748	0,028332	0,003361	0,002752	-2,58759	9,66E-03	1,45E-01	9422,5	6413,5
CONTROL-LPS+KYNA	148	215	363	1,05605	1,0616	1,039975	1,04405	1,091425	1,0879	1,069746	1,069682	0,040748	0,034995	0,003361	0,002392	-0,86569	3,87E-01	1,00E+00	15059	16761
CONTROL-SZR104	148	164	312	1,05605	1,0767	1,039975	1,05765	1,091425	1,106425	1,069746	1,087849	0,040748	0,04026	0,003361	0,003153	-4,73813	2,16E-06	3,24E-05	8365,5	15906,5
CONTROL-LPS+SZR104	148	209	357	1,05605	1,067	1,039975	1,0423	1,091425	1,1073	1,069746	1,077493	0,040748	0,044046	0,003361	0,003054	-1,69941	8,92E-02	1,00E+00	13833	17099

Lacunarity

Kruskal-Wallis H statistic=67,44662752146029, p-value=3,480008346745085e-13

SZR104-LPS+SZR104	164	209	373	0,17855	0,1157	0,107975	0,1024	0,226025	0,1834	0,171496	0,142256	0,064462	0,056705	0,005049	0,003932	-3,86766	1,10E-04	1,65E-03	21136	13140
LPS+KYNA-SZR104	215	164	379	0,1131	0,17855	0,09985	0,107975	0,12595	0,226025	0,125109	0,171496	0,044318	0,064462	0,003029	0,005049	-6,22139	4,93E-10	7,39E-09	11055,5	24204,5
LPS+KYNA-LPS+SZR104	215	209	424	0,1131	0,1157	0,09985	0,1024	0,12595	0,1834	0,125109	0,142256	0,044318	0,056705	0,003029	0,003932	-2,35191	1,87E-02	2,80E-01	19500	25435
KYNA-LPS+KYNA	107	215	322	0,118	0,1131	0,1104	0,09985	0,1303	0,12595	0,124479	0,125109	0,029211	0,044318	0,002837	0,003029	-2,52254	1,17E-02	1,75E-01	13488	9517
KYNA-SZR104	107	164	271	0,118	0,17855	0,1104	0,107975	0,1303	0,226025	0,124479	0,171496	0,029211	0,064462	0,002837	0,005049	-4,50309	6,70E-06	1,00E-04	5933,5	11614,5
KYNA-LPS+SZR104	107	209	316	0,118	0,1157	0,1104	0,1024	0,1303	0,1834	0,124479	0,142256	0,029211	0,056705	0,002837	0,003932	-0,02082	9,83E-01	1,00E+00	11198	11165
LPS-KYNA	131	107	238	0,1121	0,118	0,09805	0,1104	0,12985	0,1303	0,127583	0,124479	0,048866	0,029211	0,004286	0,002837	-2,38187	1,72E-02	2,58E-01	5749,5	8267,5
LPS-LPS+KYNA	131	215	346	0,1121	0,1131	0,09805	0,09985	0,12985	0,12595	0,127583	0,125109	0,048866	0,044318	0,004286	0,003029	-0,21608	8,29E-01	1,00E+00	13887	14278
LPS-SZR104	131	164	295	0,1121	0,17855	0,09805	0,107975	0,12985	0,226025	0,127583	0,171496	0,048866	0,064462	0,004286	0,005049	-5,33402	9,60E-08	1,44E-06	6858,5	14625,5
LPS-LPS+SZR104	131	209	340	0,1121	0,1157	0,09805	0,1024	0,12985	0,1834	0,127583	0,142256	0,048866	0,056705	0,004286	0,003932	-2,20055	2,78E-02	4,17E-01	11748	15631
CONTROL-LPS	148	131	279	0,12835	0,1121	0,1093	0,09805	0,152225	0,12985	0,154234	0,127583	0,067546	0,048866	0,005571	0,004286	-4,93907	7,85E-07	1,18E-05	13016,5	6371,5
CONTROL-KYNA	148	107	255	0,12835	0,118	0,1093	0,1104	0,152225	0,1303	0,154234	0,124479	0,067546	0,029211	0,005571	0,002837	-3,24051	1,19E-03	1,79E-02	9802	6034
CONTROL-LPS+KYNA	148	215	363	0,12835	0,1131	0,1093	0,09985	0,152225	0,12595	0,154234	0,125109	0,067546	0,044318	0,005571	0,003029	-5,62065	1,90E-08	2,85E-07	21432,5	10387,5
CONTROL-SZR104	148	164	312	0,12835	0,17855	0,1093	0,107975	0,152225	0,226025	0,154234	0,171496	0,067546	0,064462	0,005571	0,005049	-1,64075	1,01E-01	1,00E+00	10830	13442
CONTROL-LPS+SZR104	148	209	357	0,12835	0,1157	0,1093	0,1024	0,152225	0,1834	0,154234	0,142256	0,067546	0,056705	0,005571	0,003932	-2,92153	3,48E-03	5,23E-02	18273	12659

Supplementary Information

Supplementary Figure S1. Fluorescent immunocytochemistry on control and treated microglial cells. CD11b/c-immunopositive microglia with DAPI-labeled nuclei used to quantitatively analyze cytomorphological heterogeneity in secondary microglial cultures (DIV7). A total of 974 cells were analyzed (see Table S1, for details). (A) Control, unchallenged microglia (n = 148), (B) LPS-challenged microglia (n = 131), (C) KYNA-treated microglia (n = 107), (D) LPS-challenged, KYNA-treated microglia (n = 215), (E) SZR104-treated microglia (n = 164), and (F) LPS-challenged and SZR104-treated microglia (n = 209). Some images contain > 1 microglia; consequently, some cells are shown multiple times. Scale bar: 100 µm.

Supplementary Figure S2. Digital image processing and measurement pipeline (Part 1) of two-channel digital images of individual microglial cells. (**A**) CD11b/c-labeled cells and DAPI-labeled nuclei of selected individual cells were cropped to 400 × 400-pixel two-channel (CD11b/c-red and DAPI-blue) images from the original 1920 × 1440-pixel fluorescence microscopy images. (**B**) Individual DAPI-labeled nuclei observed using only the blue color channel. Each color channel can be displayed separately in ImageJ allowing for clear identification of individual cells during cell sorting (Image→Color→Channels Tool…→Convert to multi-channel composite image?→OK). (**C**) Visualization of CD11b/c-positive cytoplasm in individual cells using only the red color channel. (**D**) Microglial cell nuclei visualized in grayscale. After splitting the images of DAPI-labeled microglial cell nuclei, the image type was set to 8-bit grayscale (Image→Color→Split channels; Image→Type→8-bit). (**E**) Grayscale representation of the cytoplasm of microglial cells. After splitting the images of CD11b/c-positive microglial cytoplasms, the image type was set to 8-bit grayscale. (Image→Color→Split channels; Image→Type→8-bit). (**F**) Segmented images of individual DAPI-labeled cell nuclei. The 8-bit grayscale images of cell nuclei were transformed into binary images using the same threshold for all individual cell/cell nuclei images during segmentation (Image→Adjust→Threshold...). Scale bar: 100 µm.

Supplementary Figure S3. Digital image processing and measurement pipeline (Part 2). (**G**) Segmented images of CD11b/c-positive cytoplasm of individual cells. The 8-bit grayscale cytoplasm images were transformed into binary images using the same thresholding procedure for all individual cell images during segmentation. (Image→Adjust→Threshold...). (**H, I**) Threshold and clean binary silhouettes of individual DAPI-labeled nuclei and CD11b/c-positive cytoplasm processed to clear the background and separate cells from each other so the silhouettes would be formed by a continuous set of foreground pixels. Basic ImageJ commands were used to clean the images and, if necessary, the cells were manually separated from each other, carefully considering the original image. These figures show the silhouettes before applying the fill holes command to visualize staining complementarity (Analyze→Analyze Particles: Size (depends on the circumstances), Show: Masks, Exclude on edges; Process→Binary: Fill holes, Watershed; Freehand selections (when necessary), Edit→Cut; Wand tool, Edit→Selection→Make Inverse). Note that not all commands were necessarily applied in each case. (**J**) Complementarity of the silhouettes obtained by nuclear and cytoplasmic staining by superimposing the threshold and clear silhouettes of the nuclei and cytoplasm in their original position. These figures show the silhouettes before applying the fill holes command to visualize staining complementarity. (**K**) Merged silhouettes of corresponding microglial cytoplasm and cell nuclei showing all foreground pixels of the threshold and clean microglial cytoplasm and nucleus silhouettes. The figure shows the silhouettes after applying the fill holes command. These final silhouettes and their outlined versions were used for measurement and analysis. (**L**) Visualization of the outlined versions of the silhouettes (black contour lines enclosing the dark gray areas), the convex hull (black edges of convex polygons enclosing the dark gray areas and white concavities) and the bounding circle (black bounding circles enclosing the light gray parts as well) of these shapes. The original convex hull figures generated by FracLac for ImageJ were used to create this image; coloring is used to enhance visibility (Process→Binary→Outline). Scale bar: 100 μm.

Supplementary Figure S4. Determination of fractal dimension and lacunarity by the box counting method. The figure shows the outlined silhouettes with a series of

successive grid calibers by superimposing the different sized grids from the FracLac plugin measurements of one case for each cell silhouette from the 12 averaged different and random grid placements used to calculate the slope: Plugins→Fractal Analysis→FracLac→Box Counting (BC). The box size scale was determined as "Power series" with base 2 and exponent 2 in "Grid design" to create the successive sizes. The "Use binary" option for binary scans was selected and white was locked as background to obtain the number of boxes containing any foreground pixels of the outlined binary silhouettes. To calculate the results, "Db" (box counting fractal dimension) in the "Colour Coding" window was then selected as the type of fractal dimension. To illustrate the method, representative traced microglial cells from control (**A**) and SZR104 treated (**B**) cultures are shown. Scale bar: 100 µm.

Supplementary Table S1. Quantitative parameter values of individual microglial cells from control and treated cultures. Values for area and convex hull area are in μm^2, while the values for perimeter, convex hull perimeter, mean radius of the convex hull, maximum span across the convex hull, and diameter of the bounding circle are provided in µm. Circularity, transformation index, the ratio maximum/minimum convex hull radii, convex hull circularity, span ratio of the convex hull, density, roughness, fractal dimension, and lacunarity are dimensionless numbers. See the main text for abbreviations.

Supplementary Table S2. Statistical analysis on selected quantitative parameters of control and treated secondary microglia. The non-parametric Kruskal–Wallis H-test followed by Mann–Whitney U post-hoc analysis of multiple pairwise comparisons between groups with Bonferroni correction was performed to avoid false significancy values. See the main text for abbreviations.
